# Mitochondria-Targeted COUPY Photocages: Synthesis and Visible-Light Photoactivation in Living Cells

**DOI:** 10.1021/acs.joc.3c00387

**Published:** 2023-05-20

**Authors:** Marta López-Corrales, Anna Rovira, Albert Gandioso, Santi Nonell, Manel Bosch, Vicente Marchán

**Affiliations:** † Departament de Química Inorgànica i Orgànica, Secció de Química Orgànica, Institut de Biomedicina de la Universitat de Barcelona (IBUB), Universitat de Barcelona (UB), Martí i Franqués 1−11, E-08028 Barcelona, Spain; ‡ Institut Químic de Sarrià, Universitat Ramon Llull, Vía Augusta 390, E-08017 Barcelona, Spain; § Unitat de Microscòpia Òptica Avançada, Centres Científics i Tecnològics (CCiTUB), Universitat de Barcelona (UB), Av. Diagonal 643, E-08028 Barcelona, Spain

## Abstract

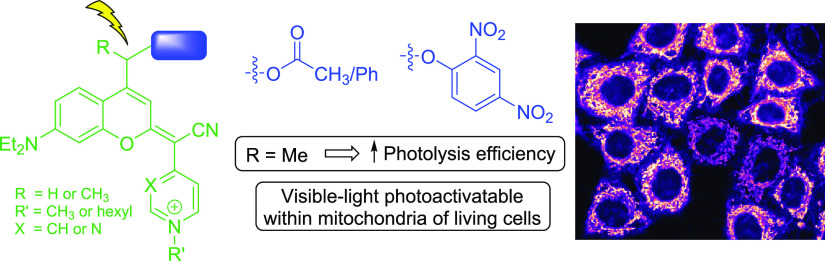

Releasing bioactive molecules in specific subcellular locations from the corresponding caged precursors offers great potential in photopharmacology, especially when using biologically compatible visible light. By taking advantage of the intrinsic preference of COUPY coumarins for mitochondria and their long wavelength absorption in the visible region, we have synthesized and fully characterized a series of COUPY-caged model compounds to investigate how the structure of the coumarin caging group affects the rate and efficiency of the photolysis process. Uncaging studies using yellow (560 nm) and red light (620 nm) in phosphate-buffered saline medium have demonstrated that the incorporation of a methyl group in a position adjacent to the photocleavable bond is particularly important to fine-tune the photochemical properties of the caging group. Additionally, the use of a COUPY-caged version of the protonophore 2,4-dinitrophenol allowed us to confirm by confocal microscopy that photoactivation can occur within mitochondria of living HeLa cells upon irradiation with low doses of yellow light. The new photolabile protecting groups presented here complement the photochemical toolbox in therapeutic applications since they will facilitate the delivery of photocages of biologically active compounds into mitochondria.

## Introduction

The development of novel photolabile protecting groups (PPGs) or caging groups that can be photoactivated with biologically compatible visible light has raised in recent years a growing interest in photopharmacology owing to the extraordinary properties of light.^1^ This noninvasive external stimulus can be delivered to living organisms with a high spatiotemporal resolution, allowing the manipulation of cellular processes by phototriggering the release of bioactive molecules from photocaged inactive precursors without using potentially toxic chemical reagents.^2^ Moreover, light of long wavelengths (e.g., far-red and near-infrared (NIR)) is non-phototoxic and offers higher tissue penetration than UV or blue light (300–400 nm), which facilitates *in vivo* applications and clinical translation.^3^ Among visible-light-sensitive PPGs based on organic chromophores, *o*-nitrobenzyl,^4^ quinone,^5^ coumarin,^6^ naphthalene,^7^ BODIPY,^8^ xanthenium,^9^ cyanine,^10^ and porphyrin^11^ derivatives have been widely used in chemical, biological, and materials science applications. Transition metal complexes with absorption in the visible region of the electromagnetic spectrum, such as ruthenium(II) polypyridyl complexes, have also been explored as caging groups.^12^ Although many efforts have been dedicated to the design of caging groups with optimal photophysical and photochemical properties (e.g., operability at long wavelengths and high photolytic efficiency),^13^ the molecular size and structural complexity of the PPG and its ease of synthesis are also important parameters sometimes underestimated when developing new caging groups for therapeutic applications. Aqueous solubility and dark stability to spontaneous hydrolysis are also important factors to be considered for newly synthesized caging groups.

Among subcellular organelles, mitochondria are one of the most relevant targets in drug design and development for combating human pathologies since they are involved in many key cellular processes.^14^ Mitochondrial dysfunction has been associated with cancer disease, aging, and neurodegenerative, cardiovascular, and metabolic diseases.^15^ In addition, mitochondria are the major sources of endogenous reactive oxygen species.^16^ A common strategy for developing mitochondria-targeted diagnostic and therapeutic tools consists of attaching lipophilic positively charged chemical motifs (e.g., triphenylphosphonium) to the compound of interest to induce mitochondria accumulation by exploiting the negative potential across the external and internal membrane of this organelle.^17^ However, this strategy implies several limitations since bulky hydrophobic groups can modify the physicochemical and pharmacological properties of the molecule of interest and, in addition, they do not provide cell or tissue specificity. The latter is especially important in anticancer therapies since toxic side effects of conventional chemotherapeutic agents are usually associated with their poor ability to discriminate between normal and cancer cells. In such a context, organelle-specific photocages offer a powerful method for delivering and releasing bioactive compounds in specific subcellular compartments by using light of suitable wavelengths, as recently described by different research groups in the case of mitochondria.^18^

**Scheme 1:**
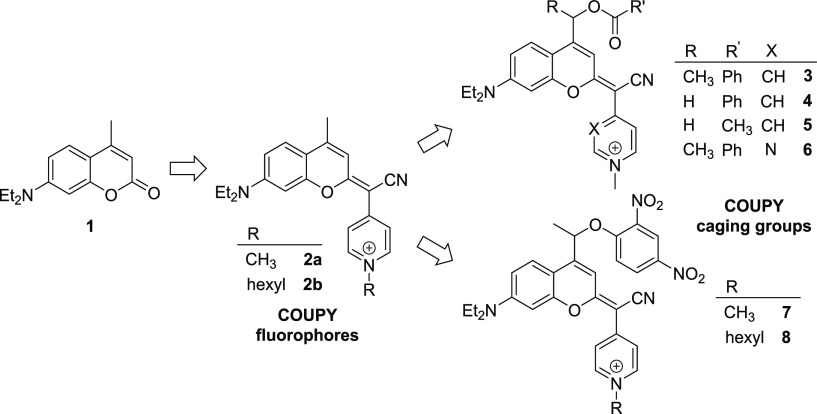
Schematic Representation of the Transformation of COUPY Fluorophores into Mitochondria-Targeted COUPY Photocages and Structures of Compounds **3–8** Investigated in This Work

Our group has developed a new class of coumarin-based fluorophores (COUPY) through the replacement of the carbonyl group of the lactone in the conventional coumarin scaffold (e.g., compound **1** in Scheme 1) by cyano(*N*-alkyl-4-pyridinium/pyrimidinium)-methylene moieties (e.g., compounds **2a** and **2b**), which exhibit promising photophysical and photochemical properties for bioimaging and therapeutic applications owing to the π-extended system.^19^ Recently, we have initiated the transformation of such coumarin derivatives into a novel class of visible-light-sensitive PPGs. As a proof of concept, COUPY photocage **3**, in which benzoic acid was caged through the formation of an ester bond through position 4 of the coumarin skeleton, was synthesized and fully characterized.^20^ Compound **3** was efficiently photoactivated with biologically compatible yellow (560 nm) and red light (620 nm) under physiological-like conditions but remained stable to spontaneous hydrolysis when incubated in the dark. Importantly, COUPY photocage **3** was found to accumulate selectively in the mitochondria of living HeLa cells according to confocal microscopy studies owing to the presence of the *N*-methylpyridinium moiety, which would facilitate the delivery of caged analogues of bioactive molecules to this organelle. Here, we synthesized three new COUPY-caged model compounds (**4–6**) to assess how the structure of the coumarin caging group influences the uncaging process, particularly how the incorporation of a methyl group in a position adjacent to the photocleavable bond in the coumarin skeleton influences the photodeprotection rate (Scheme 1). This is an important factor since the rate of the overall photolysis process in coumarin-based caging groups, including that of nonconventional dicyanocoumarin derivatives,^22^ depends on the rate constant of the initial heterolytic cleavage of the C–O bond.^13b,21^ Benzoic acid and acetic acid were selected as model compounds to be caged with COUPY coumarins through the formation of an ester bond to investigate the effect of the basicity of the leaving group, and a pyridine heterocycle was replaced by pyrimidine to further red-shift the absorption maximum of the compound.^23^ In addition, by taking advantage of the intrinsic preference of COUPY scaffolds for mitochondria, we have synthesized two COUPY-caged versions of the protonophore 2,4-dinitrophenol (DNP) (**7** and **8**) to investigate photoactivation in living cells by confocal microscopy.

## Results and Discussion

### Synthesis and Characterization of COUPY-Caged Model Compounds

**Scheme 2:**
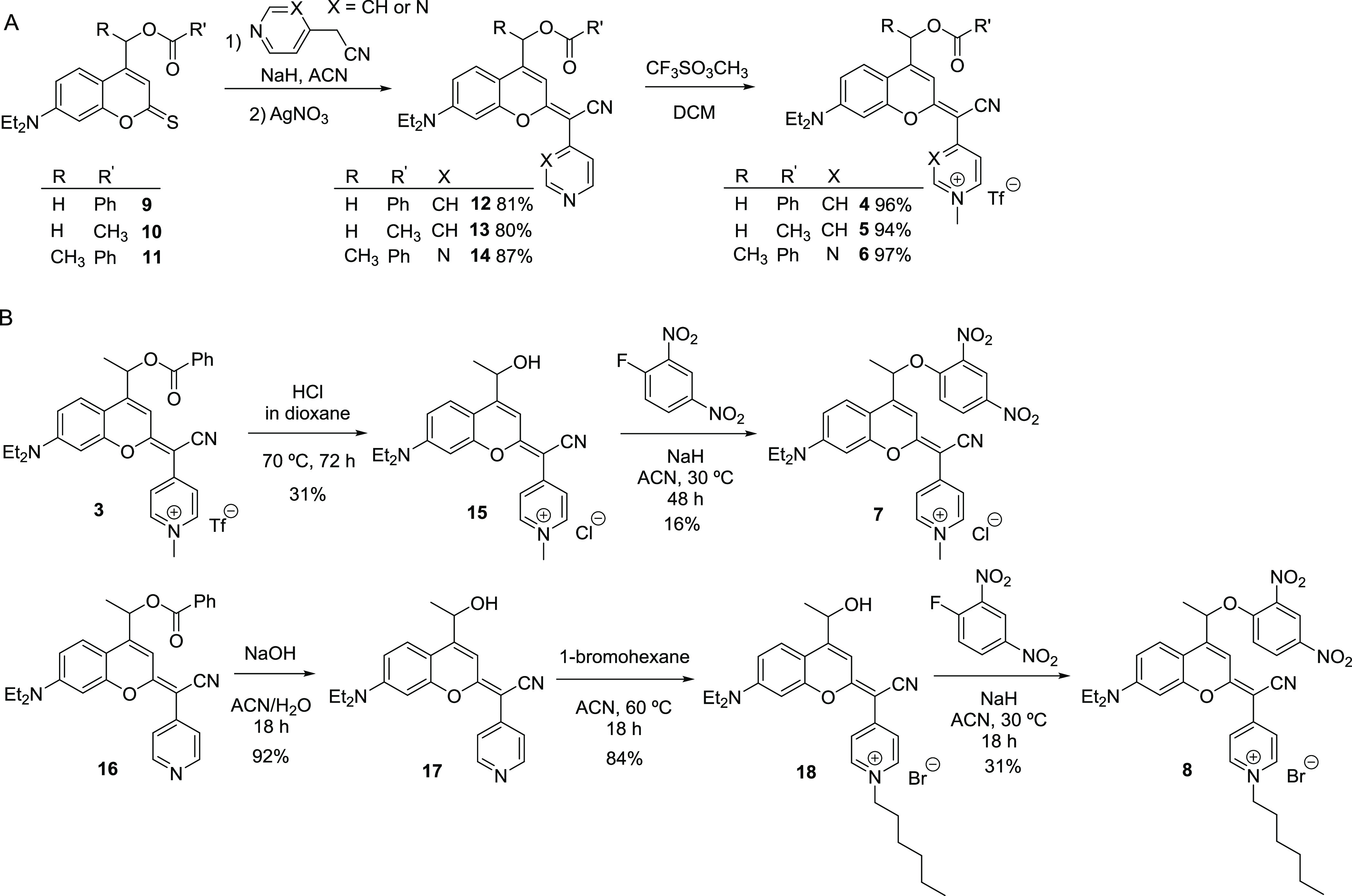
Synthesis of COUPY-Caged Model Compounds **4–6** (A) and **7** and **8** (B)

COUPY photocages **4–6** were synthesized in two steps from thiocoumarins **9–11** (Scheme 2), which were prepared from coumarin **1** following previously published procedures developed in our group.^19,22^ First, condensation of **9–11** with 4-pyridylacetonitrile or 2-(pyrimidin-4-yl)acetonitrile,^23^ mediated by the deprotonation of the acidic methylene protons with a strong base, followed by silver nitrate treatment afforded neutral COUPY scaffolds **12–14** with high yields (80–87%) after purification by silica column chromatography. After *N*-methylation of the pyridine or pyrimidine heterocycles, COUPY-caged model compounds **4–6** were isolated as pink/purple solids with excellent yields (94–97%). The compounds were fully characterized by HR ESI-MS and NMR (^1^H, ^13^C, and ^19^F), and their purity was assessed by reversed-phase HPLC-MS (Figure S1). As shown in Figures S2–S4, the ^1^H NMR spectra of coumarins **12–14** showed two sets of proton signals in ∼90/80:10/20 ratios, which reproduces the behavior previously found in COUPY derivatives^19a,19,20,23^ and demonstrates the existence of two exchangeable rotamers around the exocyclic carbon–carbon double bond. Full NMR characterization by using ^1^H,^1^H 2D-NOESY experiments confirmed that the *E* rotamer (as usually drawn in this manuscript) was the major species in solution in the case of compounds **12** and **13**. By contrast, the *Z* rotamer was preferred in the pyrimidine-containing derivative (**14**), which parallels the behavior of some pyrimidine-containing COUPY fluorophores.^23^ As previously found with *N*-methylated COUPY dyes^19a,19b^ and COUPY photocage **3**,^20^ the 1D and 2D NMR spectra revealed that only the *E* rotamer was found in solution for compounds **4–6** (Figures S7–S9).

As shown in Scheme 2, compounds **7** and **8** were synthesized by nucleophilic aromatic substitution from *N*-alkylated alcohol precursors **15** and **18**, respectively, using 1-fluoro-2,4-dinitrobenzene in the presence of a strong base (NaH) and fully characterized by HR ESI-MS and 1D and 2D NMR (Figures S10 and S11).

### Absorption and Emission Properties of COUPY Derivatives

**Figure 1:**
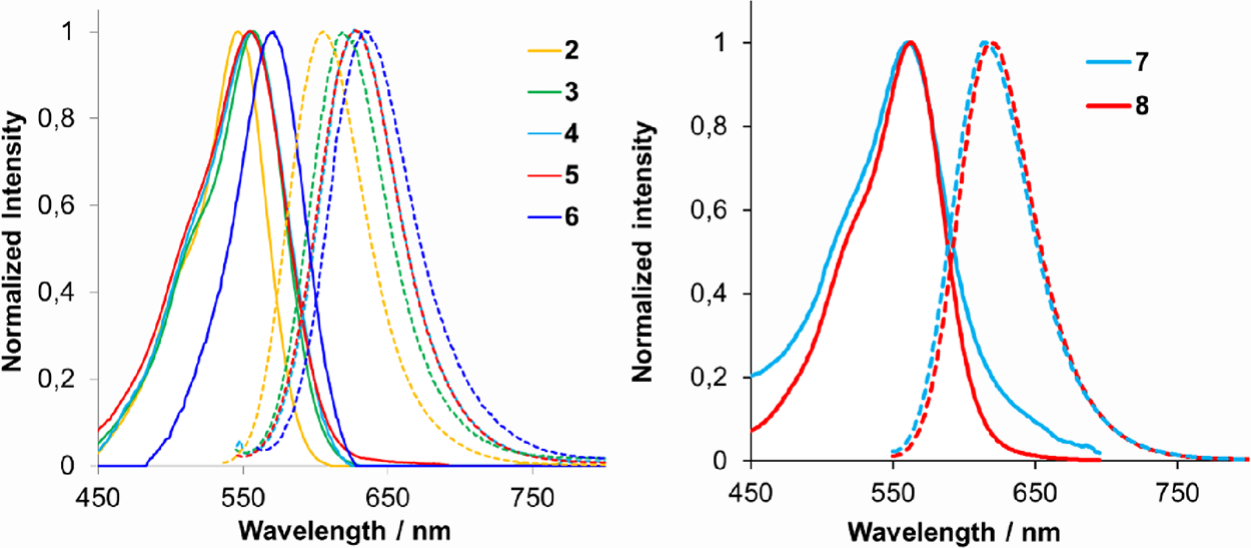
Comparison of the absorption (solid lines) and fluorescence (dotted lines) spectra of COUPY photocages **3–6** with those of COUPY dye **2a** (left) and of DNP-containing photocages **7** and **8** (right).

The photophysical properties of COUPY-caged model compounds (**4–8**) are shown in Table 1 and compared with those of the parent fluorophore (**2a**)^19a^ and COUPY photocage (**3**).^20^ As shown in Figure 1, the visible spectrum of all the compounds exhibited an intense absorption band, with absorption maxima ranging from 555 nm (**5**) to 570 nm (**6**). Esterification with both carboxylic acids caused a slight red-shift in compounds **3–5** (about 9–11 nm) relative to coumarin **2a**. Very interestingly, the replacement of pyridine with the more electron-deficient pyrimidine heterocycle caused a 13 nm red-shift in the absorption maximum of **6** with respect to **3** (24 nm when compared with **2a**) and an increase in the value of the molar absorption coefficient (ε = 59 mΜ^–1^ cm^–1^ for **6** vs 35–38 mΜ^–1^ cm^–1^ for **3–5**). Such bathochromic effects were even more pronounced for the emission wavelength of all the model caged compounds (λ_em_ = 619–634 nm) when compared with **2a** (λ_em_ = 603 nm). However, the incorporation of the methyl group on the coumarin structure caused a remarkable blue-shift in the emission maximum (10 nm) with respect the non-methylated analogues (e.g., compare **3** and **4**), which was partially compensated for in the pyrimidine-containing coumarin (e.g., compare **4** and **6**). As a result, the Stokes shifts were slightly larger in the non-methylated COUPY-caged compounds than in the methylated analogues (e.g., 73 nm for **4** vs 62 nm for **3**) but always larger than the value of the original fluorophore (57 nm in **2a**). On the contrary, fluorescent quantum yields were reduced by more than 50% in the caged compounds (e.g., Φ_F_ = 0.08–0.10 in **3–6** vs Φ_F_ = 0.22 in **2a**).

**Table 1:** Photophysical Parameters for COUPY-Caged Model Compounds (**3–8**) and COUPY Dye **2a**^a^

	absorption		emission		
compound	λ_max_ (nm)^b^	ε(λ_max_) (Μ^–1^ cm^–1^)^c^	λ_em_ (nm)^d^	Stokes shift (nm)^e^	ϕ_Em_^f^
**2a**	546	46,300	603	57	0.22
**3**	557	38,000	619	62	0.10
**4**	556	35,200	629	73	0.08
**5**	555	35,000	630	75	0.08
**6**	570	59,400	634	64	0.08
**7**	560	27,600	613	53	0.06
**8**	563	42,900	619	56	0.10

a Absorption and emission spectra were recorded in a 1:1 (v/v) mixture of PBS buffer and ACN at 25 °C.

b Wavelength of the absorption maximum.

c Molar absorption coefficient at λ_max_.

d Wavelength of the emission maximum upon excitation at 20 nm below λ_max_.

e Stokes shift.

f Fluorescence quantum yield.

Compared to COUPY photocage **3**, the incorporation of the 2,4-nitrophenol moiety in **7** and **8** caused a slight red-shift in the absorption maxima (3 and 6 nm, respectively). Interestingly, the emission properties in the case of **8** were not modified with respect to the parent compound **3**, and the same emission maximum (619 nm) and fluorescence quantum yield (Φ_F_ = 0.10) were obtained. By contrast, as indicated in Table 1, the emission maximum was slightly blue-shifted in the case of **7**, and Φ_F_ slightly reduced. Overall, these results indicate that *N*-alkylation of COUPY derivatives with a long alkyl chain (e.g., hexyl in **8** vs methyl in **7**) seems to be positive for the photophysical properties of the compound.

### Photolysis Studies of COUPY-Caged Compounds

**Figure 2:**
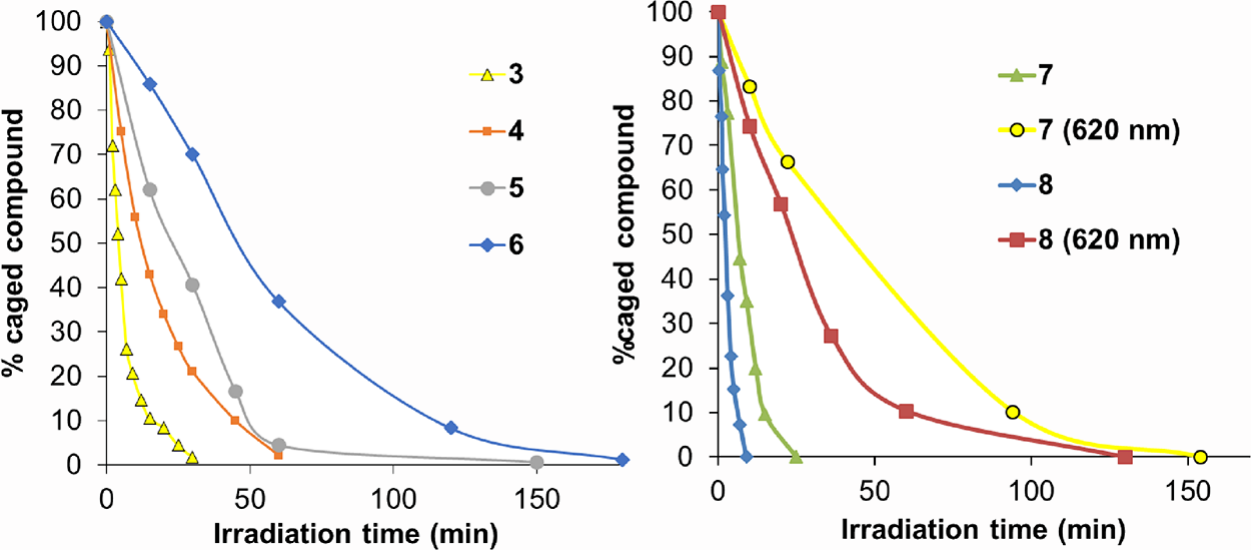
Plot of the temporal evolution of the amounts of COUPY photocages **3–6** (left) and **7** and **8** (right) after irradiation with visible LED light (470–750 nm range, centered at 530 nm; 150 mW cm^–2^; compounds **3–8**) or with red LED light (620 nm; 130 mW cm^–2^; compounds **7** and **8**). The lines connecting the experimental points are meant to aid the reader in visualizing the data. All the experiments were performed in a 1:1 (v/v) mixture of PBS buffer and ACN at 37 °C.

Photoactivation of COUPY-caged model compounds **4–6** was evaluated first in a 1:1 (v/v) mixture of PBS buffer and ACN at 37 °C after irradiation with visible LED light (Figure S12) and compared with that of the parent COUPY photocage **3**.^20^ The progress of the photolysis process was followed by HPLC-MS analysis by monitoring the disappearance of the compounds with time (Figures S13–S15). As shown in Figure 2, the concentration of all the compounds decreased gradually with irradiation time with visible light. The initial quantum yields of photolysis are collected in Table 2.

**Table 2:** Photochemical Parameters for COUPY-Caged Model Compounds^a^

compound	source (nm)^a^	solvent^a^	*k*_u_ (min^–1^)^b^	ϕ_Phot_ [× 10^–5^]^c^	ε(λ_irrad_) (Μ^–1^ cm^–1^)^d^	ε*x*ϕ_Phot_ [Μ^–1^ cm^–1^]^e^
**3**	530 nm	A	0.172			
	560 nm	A	0.031	5.4	38,000	2.1
	560 nm	B	0.099	13	48,900	6.4
**4**	530 nm	A	0.052			
	560 nm	A	0.008	1.8	35,000	0.63
	560 nm	B	0.027	15	43,100	6.5
**5**	530 nm	A	0.036			
	560 nm	A	0.004	0.75	35,000	0.26
**6**	530 nm	A	0.013			
	560 nm	A	0.003	0.66	59,000	0.39
**7**	530 nm	A	0.118			
	620 nm	A	0.019	5.1	5500	0.28
**8**	530 nm	A	0.355			
	620 nm	A	0.036	17	2600	0.44

a Irradiation was performed with visible (470–750 nm range, centered at 530 nm; 150 mW cm^–2^), yellow (560 nm; 40 mW cm^–2^), or red (620 nm; 130 mW cm^–2^) LED light in a 1:1 (v/v) (solvent A) or 4:1 (v/v) (solvent B) mixture of PBS buffer and ACN.

b Uncaging first-order rate constant.

c Photolysis quantum yields were determined from the degradation of the compounds.

d Molar absorption coefficient at the irradiation wavelength (560 or 620 nm).

e Photolytic efficiency at the irradiation wavelength.

**Scheme 3:**
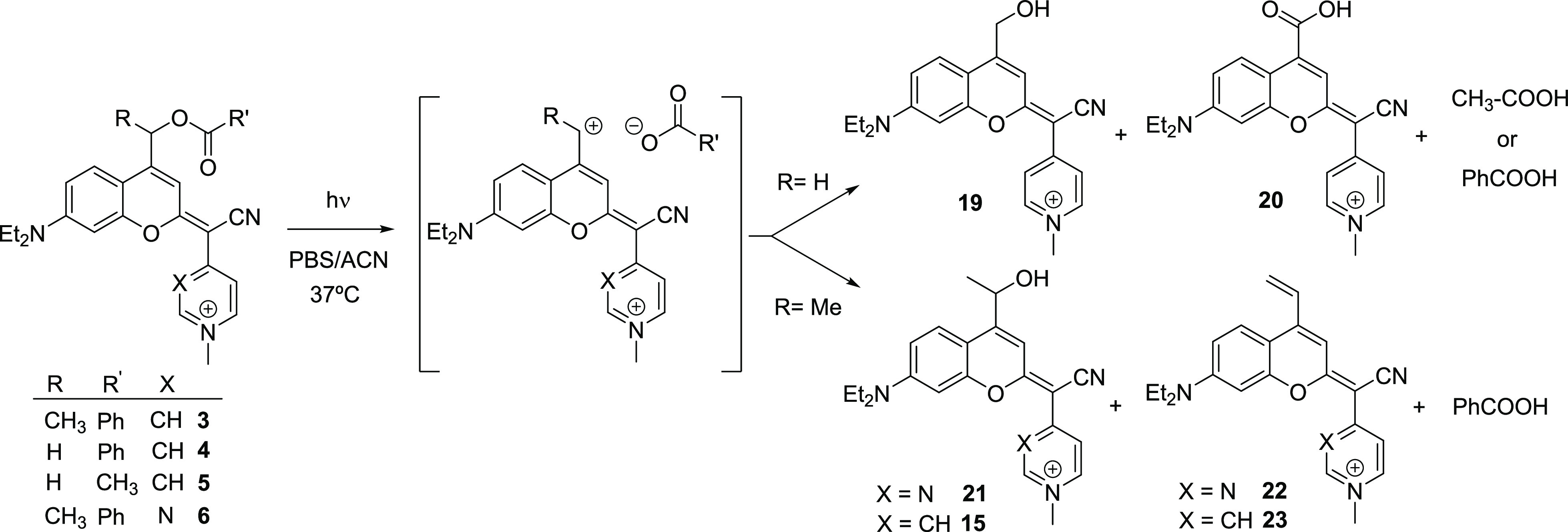
Mechanistic Interpretation of the Photolysis of COUPY-Caged Model Compounds **3–6** under Visible-Light Irradiation

In the case of compounds **4** and **5**, two main photolytic coumarins were released and identified by MS: the expected coumarin alcohol **19** and its oxidized byproduct **20** in a 3:1 relative ratio (Scheme 3). Conversely, photoactivation of compound **6** gave the coumarin alcohol **21** as the main photolytic product, as well as a minor vinyl coumarin derivative (**22**), which reproduced the results previously found for **3** where compounds **15** and **23** were also identified.^20^ In the case of compounds **3** and **6**, vinyl coumarin photoproducts are expected to be formed via a β-elimination reaction from the secondary carbocation intermediate generated upon heterolytic cleavage of the C–O bond (Scheme 3). Although the same trend was obtained when a 560 nm bandpass filter (yellow light, 40 mW cm^–2^; Figure S16) was incorporated in the LED source, the overall process was slower due to the reduced irradiance of the lamp employed in the photolysis studies. The photolytic process of compounds **3–6** was also monitored by UV–vis and fluorescence spectroscopy. As shown in Figure S17, a decrease of the absorbance of the band attributed to the coumarin core was observed in all cases, which parallels the progress of the photolysis monitored by HPLC-MS and confirmed that the phototrigger underwent photocleavage upon visible-light irradiation. The emission intensity of COUPY photocages **4–6** also decreased upon irradiation, whereas that of coumarin **3** increased, which could be attributed to a higher fluorescence quantum yield of coumarin alcohol **15** compared with **19** and **21**. The stability of the compounds to spontaneous hydrolysis was also studied in a 1:1 (v/v) mixture of PBS buffer and ACN at 37 °C (Figures S18–S21). Importantly, compounds **3–5** remained stable after incubation in the dark for 5 h at 37 °C, whereas a slight stability reduction was observed for COUPY photocage **6**.

Overall, the results from the photolysis experiments with COUPY-caged model compounds **4–6** revealed that the structure of the coumarin caging group as well as the nature of the leaving group (i.e., the carboxylic acid in our models) had a strong influence on the photoactivation process. As expected, the photolysis of compound **3** was much faster than that of **4**: the release of benzoic acid from **3** was almost complete (ca. 90%) after 15 min of irradiation with visible light, whereas it was required more than 60 min to completely uncage **4** (*k*_u_ = 0.172 min^–1^ for **3** vs *k*_u_ = 0.052 min^–1^ for **4**; see Table 2). Similar results were obtained with yellow light irradiation: compound **3** was completely uncaged after 90 min of irradiation, whereas only half of **4** was photoactivated at this time (Figure S16). As previously found in other coumarin-based caging groups,^20,22^ the higher stability of the secondary carbocation intermediate generated upon photolysis of **3** might account for this result. Hence, considering that the rate of the overall photolysis depends on the rate constant of the initial heterolytic C–O bond cleavage, the incorporation of a methyl group in a position adjacent to the photocleavable bond in the coumarin skeleton seems to be a key parameter for modulating the photoactivation process. As expected, the photocleavage process was slightly faster with **4** than with **5**, owing to the presence of a better-leaving group in the former compound (benzoate vs acetate; see Table 2).

To our surprise, the replacement of pyridine by pyrimidine (compare **3** with **6**) had a negative effect on the photosensitivity of the COUPY caging group since about 70% of the starting caged compound **6** was still present in the reaction mixture after irradiation with visible light for 30 min, while about 98% of the pyridine analogue (**3**) was uncaged at this time. Hence, the introduction of the pyrimidine heterocycle in COUPY coumarins has its *pros* and *cons* since it improves the photophysical properties of the chromophore (i.e., red-shifts absorption and emission maxima and increases the molar extinction coefficient) but slows down the uncaging process. This drawback can be likely attributed to the higher electron-withdrawing character of pyrimidine compared with pyridine, which might destabilize the carbocation component of the carbocation–carboxylate ion pair (Scheme 3) and, consequently, would lead to a decrease of the rate constant of the first bond cleavage.

Since the photoheterolysis mechanism for coumarins requires the presence of a nucleophilic solvent to avoid recombination of the ion pair by trapping of the coumarinylmethyl carbocation intermediate (e.g., via hydroxylation in aqueous media),^21^ we decided to investigate the photoactivation of COUPY-caged model compounds **3** and **4** in a 4:1 (v/v) mixture of PBS buffer and ACN to assess the effect of increasing the amount of water in the photolysis rate. As expected, reduction of the non-nucleophilic ACN co-solvent from 50 to 20% led to a 3-fold increase of the photolysis rate for both compounds when irradiated with yellow light (Figure S22 and Table 2).

**Scheme 4:**
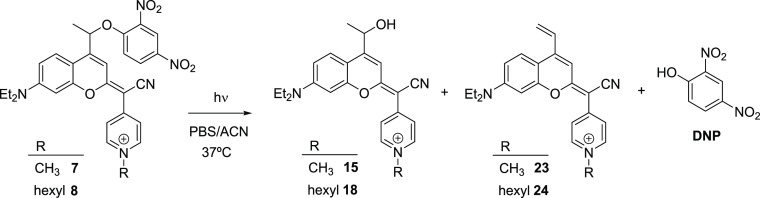
Photolysis of COUPY-Caged DNP Derivatives **7** and **8** under Visible-Light Irradiation

Next, we evaluated the photoactivation of COUPY-caged DNP derivatives **7** and **8** using visible LED light (Figures S23 and S24). To our delight, DNP was efficiently photoreleased from both compounds and a main photolytic coumarin alcohol product (**15** or **18**) was formed in both cases (Scheme 4), which demonstrates that COUPY caging groups can also be used for the protection of aromatic alcohols in addition to carboxylic acids. It is worth noting that some other minor coumarin photoproducts were also generated according to MS characterization data, including vinyl coumarins **23** and **24** (see Tables S1 and S2), which reproduced the behavior of COUPY photocages **3** and **6**. As shown in Figure 2, photolysis of the *N*-hexylpyridinium COUPY photocage (**8**) was slightly faster than that of the *N*-methylated analogue (**7**): the release of DNP from **8** was almost complete (ca. 95%) after 7 min of irradiation with visible light, whereas it required more than 20 min to completely uncage **7** (*k*_u_ = 0.118 min^–1^ for **7** vs *k*_u_ = 0.355 min^–1^ for **8**; see Table 2). Similar results were obtained by UV–vis and fluorescence spectroscopy (Figure S25). It is worth noting that both DNP-caged derivatives underwent photochemical cleavage with almost quantitative chemical yield upon visible-light irradiation when completed photolysis was achieved (94% for **7** after 25 min and 97% for **8** after 9 min), which agrees with the full consumption of the starting material according to HPLC analysis (see Figures S23, S24, and S26). Encouraged by these results and considering that our previously reported COUPY photocage **3** could be photoactivated with red light, we investigated the photosensitivity of DNP-caged derivatives **7** and **8** upon irradiation with red LED light (620 nm, 130 mW cm^–2^; Figure S12). As shown in Figure 2 and Figures S27 and S28, the concentration of both compounds decreased gradually with irradiation time, uncaging of the *N*-hexyl derivative being slightly faster than that of the *N*-methyl counterpart (*k*_u_ = 0.019 min^–1^ for **7** vs *k*_u_ = 0.036 min^–1^ for **8**; see Table 2), which parallels the results obtained with visible light. Moreover, as previously found with the benzoic acid-caged derivative **3**,^20^ DNP-caged derivatives took longer to be uncaged on irradiation with red light as compared to visible light, which is a consequence of the lower rate of light absorption.

The photolytic efficiency of the uncaging process using visible light (560 ± 40 nm; 40 mW cm^–2^) was determined as the product of the absorption coefficient at the irradiation wavelength and the photolysis quantum yield (ϕ_Phot_) calculated from the disappearance of COUPY photocages **3–8** upon irradiation (Table 2).^20^ In good agreement with the results from the photoactivation experiments, the ϕ_Phot_ for compound **3** was higher than that of the analogue lacking the methyl group adjacent to the photolabile bond (ϕ_Phot_ = 5.4 × 10^–5^ for **3** vs ϕ_Phot_ = 1.8 × 10^–5^ for **4**) under yellow light, which led to higher product ε*x*ϕ_Phot_ (2.1 for **3** vs 0.63 for **4**) since both compounds have similar molar absorption coefficients. A similar photolysis quantum yield was obtained for COUPY photocage **7** with red light (ϕ_Phot_ = 5.1 × 10^–5^). Interestingly, increasing the water percentage of the uncaging medium from 50 to 80% resulted in a remarkable enhancement in the uncaging efficiencies of COUPY photocages **3** and **4** (6.4 in PBS/ACN 4:1 vs 2.1 in PBS/ACN 1:1 for compound **3** and 6.5 vs 0.63, respectively, for compound **4**).

### Photoactivation Studies in Living HeLa Cells

**Figure 3:**
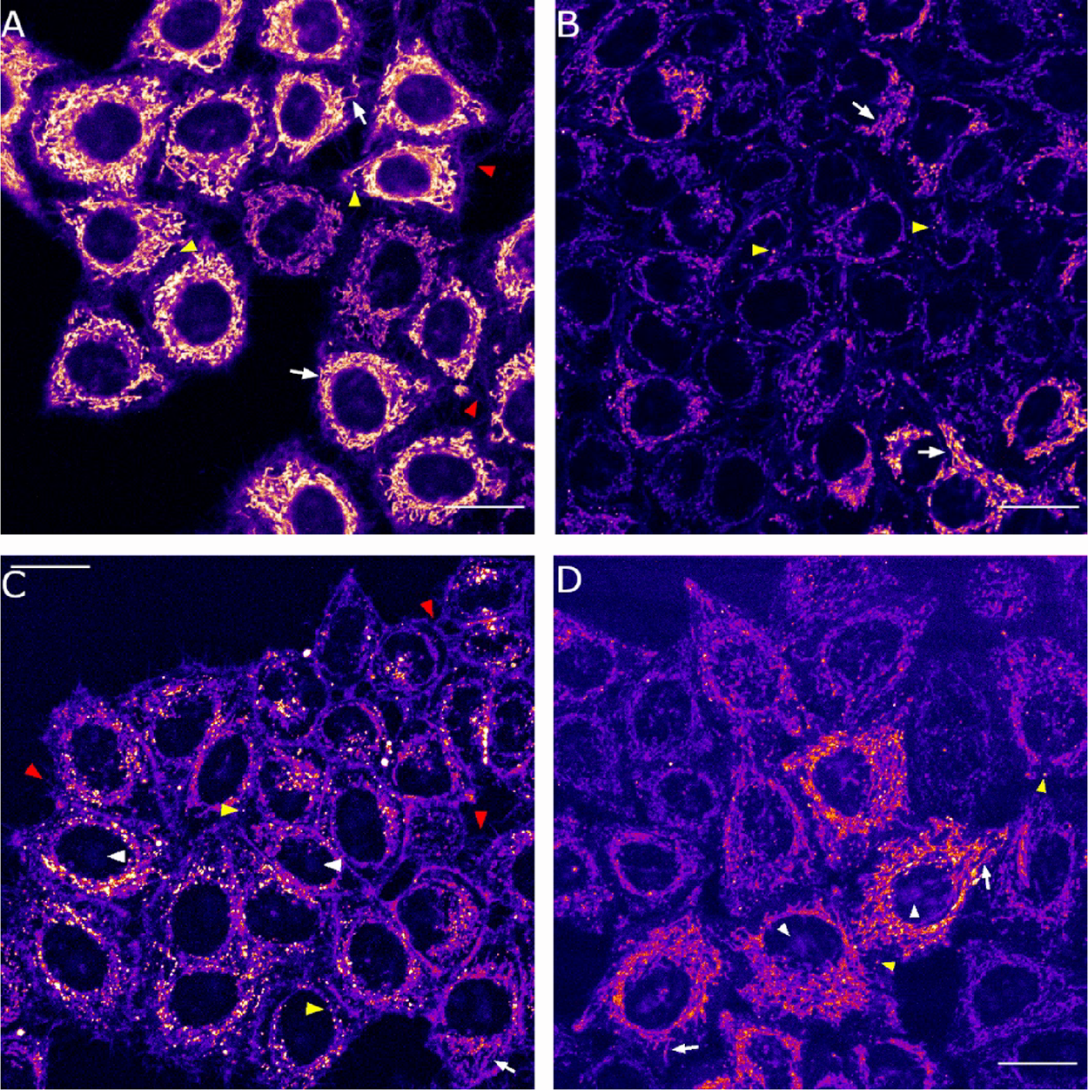
Cellular uptake of COUPY-caged DNP derivatives **8** (A) and **7** (C) and the alcohol photoproducts **18** (B) and **15** (D). Single confocal planes of HeLa cells incubated with the compounds (2 μM, 30 min, 37 °C). White arrows point out some mitochondria, white arrowheads nucleoli, yellow arrowheads vesicles, and red arrowheads membrane protrusions staining. Scale bar: 20 μm.

Once demonstrated that both COUPY-caged DNP derivatives can be efficiently photoactivated with visible light, we focused on investigating their photoactivation in living cells. First, the stability of COUPY photocages **7** and **8** in complete cell culture medium (Dulbecco’s modified Eagle’s medium (DMEM) containing high glucose and supplemented with 10% fetal bovine serum (FBS) and 50 U/mL penicillin–streptomycin) was studied. As shown in Figures S29 and S30, both compounds exhibited high dark stability upon incubation in the cell culture medium for 1 h at 37 °C. Next, the cellular uptake of compounds **7** and **8** was studied in HeLa cells (2 μM, 30 min incubation) by confocal microscopy and compared with that of the corresponding coumarin alcohol photoproducts (compounds **15** and **18**, respectively; see Scheme 4). As shown in Figure 3, the fluorescence emission signal was observed inside the cell for all the compounds after excitation at 561 nm, which confirmed an excellent cellular uptake. In the case of compound **8**, the staining pattern was similar to that previously found for the parent *N*-alkylated COUPY fluorophores (e.g., **2a** and **2b**)^19a,19e^ and COUPY photocage **3**,^20^ which suggested accumulation mainly in mitochondria. Hence, the incorporation of the DNP cargo does not alter the subcellular localization of the resulting COUPY photocage. Similarly, the photoreleased alcohol derivative (**18**) accumulated mainly in mitochondria.

Subsequent co-localization experiments with MitoTracker Green FM (MTG) confirmed the localization of both compounds in the mitochondria (Figures S31 and S32) since relatively high Pearson’s coefficients (*r* = 0.70 for **8** and *r* = 0.77 for **18**) were obtained, which indicates a clear correlation between the compounds’ signals and MTG. Similarly, the Manders’ coefficients confirmed that **8** and **18** were mainly located in the mitochondria. The degree of co-localization of **8** over MTG (M1 coefficient) was 0.57, whereas that of MTG over **8** (M2 coefficient) was 0.85. The localization of COUPY photocage **8** in other organelles (e.g., nucleoli and intracellular vesicles) could explain why there is more MTG signal co-localizing with **8** than **8** co-localizing with MTG. A similar result was obtained for coumarin alcohol **18** (M1 = 0.74 and M2 = 0.85).

To our surprise, the pattern of staining for COUPY photocage **7** was different from that of **8** and the reference compound **3** since the fluorescence signal was dispersed in different cellular compartments (nucleoli, intracellular vesicles, and cell membranes) rather than located mainly in the mitochondria (Figure 3). By contrast, coumarin alcohol **15** was located mainly in mitochondria and, to a lesser extent, in nucleoli and intracellular vesicles. Hence, the replacement of the benzoic acid cargo in our previously reported *N*-methyl COUPY photocage (**3**) by DNP (**7**) seems to alter the subcellular localization of the compound. Thus, *N*-alkylation of the pyridine heterocycle in the COUPY caging group with a long alkyl chain (e.g., hexyl) seems to be an important factor to retain mitochondria specificity in COUPY photocages, as found with compound **8**.

To investigate the photoactivation of COUPY photocage **8** within mitochondria of living HeLa cells, we followed an indirect approach described recently by Weinstain and collaborators with BODIPY photocages incorporating triphenylphosphonium as a mitochondria-targeting moiety,^18b^ which is based on the use of rhodamine 123 (Rho123), a lipophilic cationic dye that accumulates selectively in mitochondria.^24^ Since this probe is highly sensitive to changes in the mitochondrial membrane potential (Δψ_m_), the light-mediated release of DNP from **8** should induce the exit of Rho123 from mitochondria and its redistribution to the cytoplasm. This phenomenon is a consequence of the well-known ability of DNP to decrease Δψ_m_ by disrupting the proton gradient across the mitochondrial membrane.^25^ As expected, a strong mitochondria-localized fluorescence signal was observed after excitation of HeLa cells incubated with Rho123 (26 μM, 15 min) with a green light laser (λ_ex_ = 488 nm). However, as shown in Figure S33, addition of DNP caused a decrease of the overall mitochondrial fluorescence signal, which was redistributed along the cytoplasm and nucleus, thereby indicating that Rho123 was released from mitochondria due to DNP-induced modification of Δψ_m_. It is worth noting that mitochondria localization of Rho123 was not modified upon irradiation of the cells (BP 545/25 filter, 1.4 mW/cm^2^, 15 s) in the absence of DNP.

**Figure 4:**
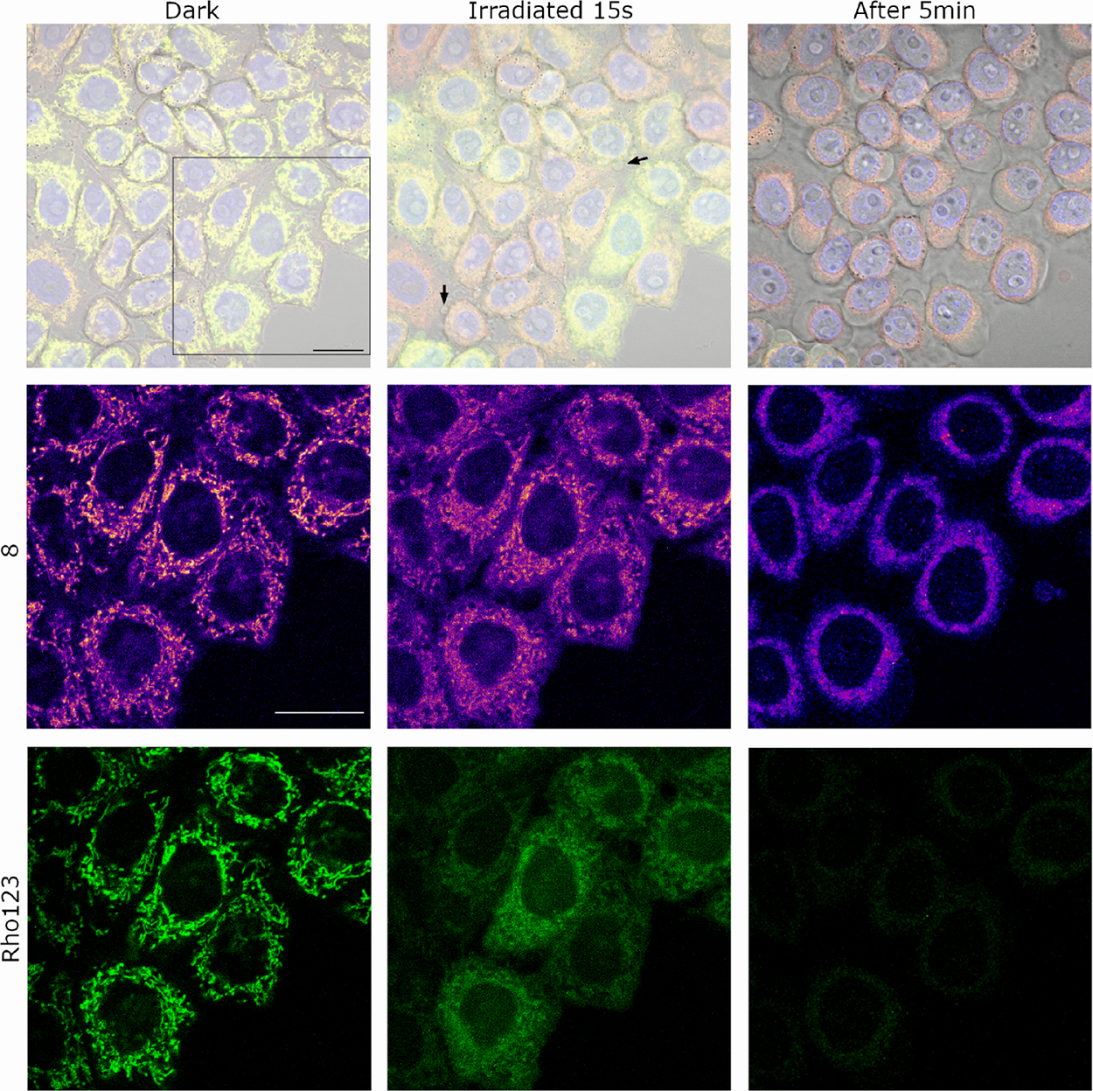
Intracellular photoactivation of COUPY photocage **8** (2 μM) in the presence of Rho123 (26 μM). Single confocal planes of HeLa cells incubated with the compounds (15 min, 37 °C) before and after irradiation (BP 545/25 filter, 1.4 mW/cm^2^, 15 s) and after standing for 5 min in the dark after irradiation. Top: **8** (red), Rho123 (green), Hoechst (blue), and bright-field merged images. Middle (**8**, Fire LUT) and bottom (Rho123, green): insets from the black square in the top row. Black arrows on top point out cell blebbings. Scale bar: 20 μm.

Once demonstrated the sensitivity of Rho123 to the external addition of DNP in our cell experiment, we focused on investigating if DNP was photoreleased from COUPY photocage **8** in live cells. For this purpose, HeLa cells were incubated with Rho123 (26 μM) and COUPY photocage **8** (2 μM) for 30 min in the dark. As shown in Figure 4, both compounds localized in mitochondria, leading to a perfect correlation between Rho123 and COUPY photocage **8** signals (Figure S32), as inferred by the high Pearson coefficient (*r* = 0.88), which confirms that COUPY photocage does not disrupt the mitochondrial membrane potential by itself. This was supported by the Manders’ coefficients since the degree of co-localization of **8** over Rho123 (M1 coefficient) was 0.80, whereas that of Rho123 over **8** (M2 coefficient) was 0.87. To our delight, the Rho123 mitochondrial fluorescence intensity was clearly reduced (ca. 40%) upon irradiation of the cells with yellow light (BP 545/25 filter, 1.4 mW/cm^2^) for 15 s (Figure 4 and Figure S34), which confirmed the photorelease of DNP from COUPY photocage **8**. By contrast, compound **8** fluorescence intensity remained unaltered. It is worth noting that the photoreleased coumarin alcohol **18** was not sensitive to changes in the Δψ_m_ since no significant changes in the mitochondrial fluorescence intensity were observed upon incubation of HeLa cells with **18** alone and after the addition of DNP (Figure S35).

## Conclusions

In summary, we have synthesized and fully characterized five new COUPY photocages for the protection of carboxylic acids (**4–6**) and 2,4-dinitrophenol (**7** and **8**) to investigate how the structure of the caging group affects the rate and efficiency of the photoactivation process compared with our previously described COUPY photocage **3**,^20^ as well as if uncaging can be triggered in living cells. All COUPY-caged model compounds exhibit attractive photophysical and physicochemical properties for use in biological applications, such as absorption in the visible region (λ_max_ ranging from 555 to 570 nm), large molar extinction coefficients (27.6–59.4 M^–1^ cm^–1^), and moderate aqueous solubility. The newly synthesized COUPY photocages were found stable to spontaneous hydrolysis when incubated in cell culture medium in the dark, and they could be efficiently photoactivated by yellow and red light in phosphate-buffered saline medium. Photolysis studies have demonstrated that the incorporation of a methyl group in the position adjacent to the photocleavable bond in the coumarin structure is particularly important to fine-tune the photochemical properties of the resulting caging group. Additionally, the use of a COUPY-caged version of the protonophore 2,4-dinitrophenol allowed us to confirm that photoactivation can occur within the mitochondria of living HeLa cells upon irradiation with low doses of yellow light. The new PPGs presented here complement the photochemical toolbox since they will facilitate the delivery and release of photocages of bioactive molecules into mitochondria for therapeutic applications. Work is in progress in our laboratory to further improve the photophysical and photochemical properties of COUPY-based caging groups through modification of the coumarin scaffold.

## Experimental Section

### Materials and Methods

Unless otherwise stated, common chemicals and solvents (HPLC grade or reagent grade quality) were purchased from commercial sources and used without further purification. A hot plate magnetic stirrer, together with an aluminum reaction block of the appropriate size, was used as the heating source in all reactions requiring heat. Aluminum plates coated with a 0.2 mm thick layer of silica gel 60 F_254_ were used for thin-layer chromatography (TLC) analyses, whereas column chromatography purification was carried out using silica gel 60 (230–400 mesh). Reversed-phase high-performance liquid chromatography (HPLC) analyses were carried out on Jupiter Proteo C_12_ columns (column 1, 250 × 4.6 mm, 90 Å 4 μm; column 2, 250 × 4.6 mm, 90 Å 4 μm; flow rate, 1 mL/min) using linear gradients of 0.1% formic acid in H_2_O (A) and 0.1% formic acid in ACN (B). The NMR spectra were recorded at 25 or 75 °C in a 400 MHz spectrometer using the deuterated solvent as an internal deuterium lock. The residual protic signal of chloroform or DMSO was used as a reference in the ^1^H and ^13^C NMR spectra recorded in CDCl_3_ or DMSO-*d*_6_, respectively. Chemical shifts are reported in part per million (ppm) in the δ scale, coupling constants in Hz, and multiplicity as follows: s (singlet), d (doublet), t (triplet), q (quartet), qt (quintuplet), m (multiplet), dd (doublet of doublets), dq (doublet of quartets), br (broad signal), etc. The proton signals of the *E* and *Z* rotamers were identified by simple inspection of the ^1^H spectrum, and the rotamer ratio was calculated by peak integration. The 2D-NOESY spectra were acquired in CDCl_3_ with mixing times of 500 ms. The electrospray ionization mass spectra (ESI-MS) were recorded on an instrument equipped with a single quadrupole detector coupled to an HPLC and high-resolution (HR) ESI-MS on an LC/MS-TOF instrument.

#### Synthesis of COUPY Scaffolds (**12**–**18**)

##### Compound **12**

4-Pyridylacetonitrile hydrochloride (400 mg, 2.60 mmol) and NaH (60% dispersion in mineral oil, 210 mg, 5.2 mmol) were dissolved in anhydrous ACN (30 mL) under an argon atmosphere. After stirring for 15 min at room temperature, a solution of thiocoumarin derivative **9**(22) (0.5 g, 1.36 mmol) in a 1:1 mixture of anhydrous ACN and DCM (30 mL) was added dropwise under Ar, and the reaction mixture was stirred at room temperature for 2 h and protected from light. Then, silver nitrate (0.57 mg, 3.41 mmol) was added and the mixture was stirred at room temperature for 2 h. The crude was evaporated under reduced pressure and purified by column chromatography (silica gel, 50–100% DCM in hexanes, and then 0–2.5% MeOH in DCM) to give 500 mg of an orange solid (81% yield). TLC: *R_f_* (5% MeOH in DCM) 0.45; ^1^H NMR (400 MHz, CDCl_3_) δ (ppm): (major rotamer, *E*) 8.61 (2H, m), 8.11 (2H, m), 7.74 (2H, m), 7.60 (1H, m), 7.48 (2H, m), 7.34 (1H, d, *J* = 8.8 Hz), 7.05 (1H, br t), 6.58 (1H, dd, *J* = 8.8, 2.6 Hz), 6.46 (1H, d, *J* = 2.6 Hz), 5.40 (2H, d, *J* = 1.2 Hz), 3.45 (4H, q, *J* = 7.2 Hz), 1.24 (6H, t, *J* = 7.2 Hz); ^13^C{^1^H} NMR (101 MHz, CDCl_3_) δ (ppm): (major rotamer, *E*) 166.1, 162.7, 154.7, 150.9, 150.0, 140.6, 140.4, 133.7, 129.9, 128.8, 124.9, 122.8, 121.1, 119.3, 111.5, 109.4, 107.3, 97.3, 84.2, 62.4, 44.8, 12. 7; HRMS (ESI-TOF) *m*/*z* [M + H]^+^ calcd for C_28_H_26_N_3_O_3_ 452.1969, found 452.1972.

##### Compound **13**

4-Pyridylacetonitrile (822 mg, 5.3 mmol) and NaH (60% dispersion in mineral oil, 426 mg, 10.6 mmol) were dissolved in anhydrous ACN (20 mL) under an argon atmosphere. After stirring for 15 min at room temperature, a solution of thiocoumarin **10**(22) (650 mg, 2.13 mmol) in anhydrous ACN (15 mL) was added, and the reaction mixture was stirred at room temperature for 2 h and protected from light. Then, silver nitrate (905 mg, 5.30 mmol) was added and the mixture was stirred at room temperature for 3 h. The crude was evaporated under reduced pressure and purified by column chromatography (silica gel, 0–4% MeOH in DCM) to give 662 mg of a purple solid (80% yield). TLC: *R_f_* (5% MeOH in DCM) 0.45; ^1^H NMR (400 MHz, CDCl_3_) δ (ppm): (major rotamer, *E*) 8.60 (2H, m), 7.73 (2H, m), 7.24 (1H, d, *J* = 9.2 Hz), 6.89 (1H, br t, *J* = 1.2 Hz), 6.56 (1H, dd, *J* = 9.2, 2.4 Hz), 6.44 (1H, d, *J* = 2.4 Hz), 5.17 (2H, d, *J* = 1.2 Hz), 3.44 (4H, q, *J* = 7.2 Hz), 2.21 (3H, s), 1.23 (6H, t, *J* = 7.2 Hz); ^13^C{^1^H} NMR (101 MHz, CDCl_3_) δ (ppm): (major rotamer, *E*) 170.5, 162.8, 154.6, 150.9, 150.0, 140.6, 140.4, 124.6, 121.1, 119.3, 110.9, 109.4, 107.1, 97.3, 84.0, 61.7, 44.8, 21.0, 12.6; HRMS (ESI-TOF) *m*/*z* [M + H]^+^ calcd for C_23_H_24_N_3_O_3_ 390.1812, found 390.1809.

##### Compound **14**

2-(Pyrimidin-4-yl)acetonitrile^23^ (187 mg, 1.57 mmol) and NaH (60% dispersion in mineral oil, 70 mg, 1.73 mmol) were dissolved in a mixture of anhydrous ACN (30 mL) and DCM (10 mL) under an argon atmosphere. After stirring for 15 min at room temperature, a solution of thiocoumarin derivative **11**(22) (300 g, 0.79 mmol) in anhydrous ACN (10 mL) was added dropwise under Ar, and the reaction mixture was stirred at room temperature for 2 h and protected from light. Then, silver nitrate (334 mg, 1.97 mmol) was added and the mixture was stirred at room temperature for 2 h. The crude was evaporated under reduced pressure and purified by column chromatography (silica gel, 50–100% DCM in hexanes, and then 0–3.5% MeOH in DCM) to give 319 mg of an orange solid (87% yield). TLC: *R_f_* (5% MeOH in DCM) 0.60; ^1^H NMR (400 MHz, CDCl_3_) δ (ppm): (major rotamer, *Z*) 8.84 (1H, br s), 8.78 (1H, d, *J* = 1.2 Hz), 8.52 (1H, d, *J* = 5.6 Hz), 8.22 (2H, m), 7.64 (1H, m), 7.52 (3H, m), 7.45 (1H, d, *J* = 8.8 Hz), 6.65 (1H, dd, *J* = 8.8, 2.4 Hz), 6.62 (1H, d, *J* = 2.4 Hz), 6.38 (1H, q, *J* = 6.8 Hz), 3.46 (4H, q, *J* = 7.2 Hz), 1.76 (3H, d, *J* = 6.8 Hz), 1.23 (6H, t, *J* = 7.2 Hz); ^13^C{^1^H} NMR (101 MHz, CDCl_3_) δ (ppm): (major rotamer, *Z*) 166.5, 165.5, 161.1, 158.9, 157.4, 156.5, 156.4, 155.3, 151.2, 149.4, 133.7, 129.9, 128.7, 124.9, 118.8, 117.7, 109.9, 108.3, 107.7, 97.8, 81.6, 68.5, 44.9, 21.4, 12.7; HRMS (ESI-TOF) *m*/*z* [M + H]^+^ calcd for C_28_H_26_N_4_O_3_ 467.2078, found 467.2074.

##### Compound **15**

To a solution of compound **3**(19) (194 mg, 0.308 mmol) in MeOH (120 mL) under an Ar atmosphere, hydrochloric acid in dioxane 4 M (30 mL, 120 mmol) was added. The mixture was stirred at 70 °C for 72 h. After removal of the solvent under reduced pressure, the product was purified by column chromatography (silica gel, 0–15% MeOH in DCM) to give 51 mg (31% yield) of a pink solid. TLC: *R_f_* (10% MeOH in DCM) 0.26; ^1^H NMR (400 MHz, DMSO-*d*_6_) δ (ppm): 8.63 (2H, d, *J* = 6.8 Hz), 8.16 (2H, d, *J* = 6.8 Hz), 7.74 (1H, d, *J* = 9.2 Hz), 7.17 (1H, s), 7.01 (1H, s), 6.91 (1H, dd, *J* = 9.2, 2.4 Hz), 5.81 (1H, d, *J* = 4.0 Hz), 5.24 (1H, m), 4.19 (3H, s), 3.54 (4H, q, *J* = 7.2 Hz), 1.42 (3H, d, *J* = 6.8 Hz), 1.16 (6H, t, *J* = 7.2 Hz); ^13^C{^1^H} NMR (101 MHz, DMSO-*d*_6_) δ (ppm): 167.2, 159.8, 154.9, 151.5, 148.3, 144.0, 126.1, 120.8, 118.4, 111.6, 107.3, 105.9, 78.9, 79.34, 63.9, 46.1, 44.2, 24.6, 12.4; HRMS (ESI-TOF) *m*/*z* [M + H]^+^ calcd for C_23_H_26_N_3_O_2_^+^ 376.2020, found 376.2026.

##### Compound **16**

4-Pyridylacetonitrile hydrochloride (810 g, 5.24 mmol) and NaH (60% dispersion in mineral oil, 420 mg, 10.5 mmol) were dissolved in anhydrous ACN (50 mL) under an argon atmosphere. After stirring for 15 min at room temperature, a solution of 1-(7-(diethylamino)-2-thioxo-2H-chromen-4-yl)ethyl benzoate^19^ (1.0 g, 2.62 mmol) in a 1:1 mixture of anhydrous ACN and DCM (50 mL) was added dropwise under Ar, and the reaction mixture was stirred at room temperature for 2 h and protected from light. Then, silver nitrate (1.11 g, 6.55 mmol) was added and the mixture was stirred at room temperature for 2 h. The crude was evaporated under reduced pressure and purified by column chromatography (silica gel, 50–100% DCM in hexane, and then 0–2.5% MeOH in DCM) to give 1.07 of an orange solid (87% yield). TLC: *R_f_* (5% MeOH in DCM) 0.45; ^1^H NMR (400 MHz, CDCl_3_) δ (ppm): (major rotamer, *E*) 8.60 (2H, d, *J* = 6 Hz), 8.12 (2H, m), 7.72 (2H, m), 7.60 (1H, m), 7.49 (2H, m), 7.45 (1H, d, *J* = 9.2 Hz), 7.06 (1H, br d, *J* = 0.4 Hz), 6.59 (1H, dd, *J* = 9.2, 2.4 Hz), 6.46 (1H, d, *J* = 2.4 Hz), 6.30 (1H, q, *J* = 6.6 Hz), 3.45 (4H, q, *J* = 7.2 Hz), 1.75 (3H, d, *J* = 6.6 Hz), 1.24 (3H, t, *J* = 7.2 Hz); ^13^C{^1^H} NMR (101 MHz, CDCl_3_) δ (ppm): (major rotamer, *E*) 165.7, 163.2, 155.0, 150.8, 149.9, 146.6, 140.8, 133.6, 129.9, 129.7, 128.8, 125.0, 121.0, 119.5, 109.4, 109.3, 106.8, 97.5, 83.7, 68.3, 44.8, 21.0, 12.7; HRMS (ESI-TOF) *m*/*z* [M + H]^+^ calcd for C_29_H_28_N_3_O_3_ 466.2125, found 466.2122.

##### Compound **17**

To a solution of coumarin **16**(20) (40 mg, 0.09 mmol) in a 2:1 (v/v) mixture of ACN and Milli-Q H_2_O (30 mL), a solution of sodium hydroxide 0.25 M (1.08 mL, 0.27 mmol) was added and the reaction mixture was stirred overnight at room temperature. After removal of the solvent under pressure, the product was purified by column chromatography (silica gel, 50–100% DCM in hexanes, and then 0.25–4% MeOH in DCM) to give 30 mg of an orange solid (92% yield). TLC: *R_f_* (5% MeOH in DCM) 0.38; ^1^H NMR (400 MHz, DMSO-*d*_6_) δ (ppm): (major rotamer, *E*) 8.55 (2H, m), 7.72 (2H, m), 7.52 (1H, d, *J* = 9.2 Hz), 6.96 (1H, s), 6.71 (1H, s), 6.70 (1H, m), 5.61 (1H, d, *J* = 4.0 Hz), 5.06 (1H, m), 3.46 (4H, q, *J* = 7.2 Hz), 1.40 (3H, d, *J* = 6.4 Hz), 1.14 (6H, t, *J* = 7.2 Hz). ^13^C{^1^H} NMR (101 MHz, DMSO-*d*_6_) δ (ppm): (major rotamer, *E*) 163.7, 154.2, 153.6, 150.4, 140.1, 125.4, 120.1, 119.4, 109.5, 106.4, 106.3, 96.9, 80.5, 63.9, 43.8, 24.2, 12.4; HRMS (ESI-TOF) *m*/*z* [M + H]^+^ calcd for C_22_H_23_N_3_O_2_ 362.1869, found 362.1872.

##### Compound **18**

To a solution of coumarin **17** (30 mg, 0.083 mmol) in ACN anhydrous (3 mL), 1-bromohexane (0.6 mL, 4.15 mmol) was added under an Ar atmosphere and the reaction mixture was stirred overnight at 60 °C. After removal of the solvent under reduced pressure, the product was purified by column chromatography (silica gel, 0–7% MeOH in DCM) to give 31 mg of a pink solid (84% yield). TLC: *R_f_* (10% MeOH in DCM) 0.55. ^1^H NMR (400 MHz, DMSO-*d*_6_) δ (ppm): 8.70 (2H, d, *J* = 7.2 Hz), 8.18 (2H, d, *J* = 7.2 Hz), 7.75 (1H, d, *J* = 9.6 Hz), 7.18 (1H, s), 7.01 (1H, br s), 6.93 (1H, dd, *J* = 9.6, 2.4 Hz), 5.80 (1H, d, *J* = 4.0 Hz), 5.24 (1H, m), 4.44 (1H, t, *J* = 7.2 Hz), 3.55 (4H, t, *J* = 7.2 Hz), 1.87 (2H, m), 1.42 (3H, d, *J* = 6.8 Hz), 1.29 (6H, m), 1.18 (6H, t, *J* = 7.2 Hz), 0.86 (3H, m); ^13^C{^1^H} NMR (101 MHz, DMSO-*d*_6_) δ (ppm): 167.3, 159.9, 155.0, 151.6, 148.6, 143.0, 126.2, 120.9, 118.4, 111.7, 107.4, 105.9, 96.7, 79.0, 64.0, 58.8, 44.1, 30.7, 30.5, 25.1, 24.6, 21.9, 13.8, 12.4; HRMS (ESI-TOF) *m*/*z* [M + H]^+^ calcd for C_28_H_36_N_3_O_2_^+^ 446.2802, found 446.2797.

#### Synthesis of COUPY-Caged Compounds (**3**–**8**)

##### Compound **3**

Methyl trifluoromethanesulfonate (127 μL, 1.12 mmol) was added to a solution of compound **16**(20) (260 mg, 0.56 mmol) in DCM (100 mL) under an Ar atmosphere. The mixture was stirred overnight at room temperature and protected from light. After removing the solvent under reduced pressure, purification by column chromatography (silica gel, 0–6% MeOH in DCM) afforded 340 mg (98% yield) of a pink solid. TLC: *R_f_* (10% MeOH in DCM) 0.45; ^1^H NMR (400 MHz, DMSO-*d*_6_) δ (ppm): 8.61 (2H, d, *J* = 7.2 Hz), 8.16 (2H, d, *J* = 7.2 Hz), 8.08 (2H, m), 7.90 (1H, d, *J* = 9.2 Hz), 7.73 (1H, m), 7.59 (2H, m), 6.99 (3H, m), 6.44 (1H, q, *J* = 6.6 Hz), 4.19 (3H, s), 3.56 (4H, q, *J* = 7.2 Hz), 1.73 (3H, d, *J* = 6.6 Hz), 1.18 (6H, t, *J* = 7.2 Hz); ^13^C{^1^H} NMR (101 MHz, DMSO-*d*_6_) δ (ppm): 166.6, 164.7, 155.2, 153.5, 151.8, 148.0, 144.1, 134.0, 129.3, 129.0, 126.2, 121.1, 120. 7 (q, *J* = 322 Hz), 118.1, 111.8, 106.6, 105.7, 97.0, 79.8, 68.8, 54.9, 46.2, 44.2, 21.0, 12.4; ^19^F NMR (376.5 MHz, DMSO-*d*_6_): −77.8 (3F, s); HRMS (ESI-TOF) *m*/*z* [M]^+^ calcd for C_30_H_30_N_3_O_3_^+^ 480.2282, found 480.2279; analytical HPLC (10 to 100% B over 30 min, column 2) *R*_t_ = 9.8 min.

##### Compound **4**

Methyl trifluoromethanesulfonate (60 μL, 0.53 mmol) was added to a solution of compound **12** (120 mg, 0.27 mmol) in DCM (60 mL) under an Ar atmosphere. The mixture was stirred overnight at room temperature and protected from light. After removing the solvent under reduced pressure, purification by column chromatography (silica gel, 0–6% MeOH in DCM) afforded 160 mg (96% yield) of a pink solid. TLC: *R_f_* (10% MeOH in DCM) 0.50; ^1^H NMR (400 MHz, DMSO-*d*_6_) δ (ppm): 8.63 (2H, d, *J* = 7.2 Hz), 8.18 (2H, d, *J* = 7.2 Hz), 8.05 (2H, m), 7.73 (2H, m), 7.58 (2H, m), 7.05 (1H, br s), 6.99 (1H, br s), 6.96 (1H, dd, *J* = 9.2, 2.4 Hz), 5.70 (2H, s), 4.21 (3H, s), 3.55 (4H, q, *J* = 7.2 Hz), 1.18 (6H, t, *J* = 7.2 Hz); ^13^C{^1^H} NMR (101 MHz, DMSO-*d*_6_) δ (ppm): 166.4, 166.0, 154.9, 151.84, 148.0, 147.7, 144.1, 134.0, 129.3, 129.0, 128.9, 126.2, 121.2, 120.7 (q, *J* = 323 Hz), 118.0, 111.7, 107.9, 107.3, 96.7, 79.9, 62.2, 46.2, 44.2, 12.4; ^19^F NMR (376.5 MHz, DMSO-*d*_6_): −77.8 (3F, s); HRMS (ESI-TOF) *m*/*z* [M]^+^ calcd for C_29_H_28_N_3_O_3_^+^ 466.2125, found 466.2125; analytical HPLC (10 to 100% B over 30 min, column 2) *R*_t_ = 9.5 min.

##### Compound **5**

Methyl trifluoromethanesulfonate (174 μL, 1.54 mmol) was added to a solution of compound **13** (300 mg, 0.77 mmol) in DCM (100 mL) under an Ar atmosphere. The mixture was stirred overnight at room temperature and protected from light. After removing the solvent under reduced pressure, purification by column chromatography (silica gel, 0–6% MeOH in DCM) afforded 400 mg (94% yield) of a pink solid. TLC: *R_f_* (10% MeOH in DCM) 0.45; ^1^H NMR (400 MHz, DMSO-*d*_6_) δ (ppm): 8.62 (2H, d, *J* = 7.2 Hz), 8.17 (2H, d, *J* = 6.8 Hz), 7.62 (1H, d, *J* = 9.2 Hz), 6.96 (1H, br s), 6.92 (1H, dd, *J* = 9.2, 2.4 Hz), 6.87 (1H, br s), 5.40 (2H, s), 4.19 (3H, s), 3.53 (4H, q, *J* = 7.2 Hz), 2.15 (3H, s), 1.16 (6H, t, *J* = 7.2 Hz); ^13^C{^1^H} NMR (101 MHz, DMSO-*d*_6_) δ (ppm): 169.8, 166.4, 154.8, 151.8, 148.0, 147.8, 144.1, 126.2, 121.2, 120.7 (q, *J* = 322 Hz), 119.1, 118.8 (q, *J* = 322 Hz), 118.0, 111.7, 107.7, 107.3, 96.7, 79.8, 61.4, 54.9, 46.2, 42.2, 20.5, 12.4; ^19^F NMR (376.5 MHz, DMSO-*d*_6_): −77.8 (3F, s); HRMS (ESI-TOF) *m*/*z* [M]^+^ calcd for C_24_H_26_N_3_O_3_^+^ 404.1969, found 404.1970; analytical HPLC (10 to 100% B over 30 min, column 2) *R*_t_ = 8.3 min.

##### Compound **6**

Methyl trifluoromethanesulfonate (55 μL, 0.50 mmol) was added to a solution of compound **14** (78 mg, 0.17 mmol) in DCM (30 mL) under an Ar atmosphere. The mixture was stirred overnight at room temperature and protected from light. After removing the solvent under reduced pressure, purification by column chromatography (silica gel, 0–6.5% MeOH in DCM) afforded 102 mg (97% yield) of a purple solid. TLC: *R_f_* (15% MeOH in DCM) 0.45; ^1^H NMR (400 MHz, DMSO-*d*_6_, 75 °C) δ (ppm): 9.02 (1H, br s), 8.59 (1H, dd, *J* = 7.4, 1.8 Hz), 8.09 (2H, m), 8.03 (1H, br s), 8.00 (1H, d, *J* = 9.2 Hz), 7.85 (1H, br s), 7.73 (1H, m), 7.61 (2H, m), 7.12 (1H, dd, *J* = 9.2, 2.8 Hz), 6.94 (1H, d, *J* = 2.8 Hz), 6.52 (1H, q, *J* = 6.6 Hz), 4.03 (3H, s), 3.61 (4H, q, *J* = 7.2 Hz), 1.76 (3H, d, *J* = 6.6 Hz), 1.23 (6H, t, *J* = 7.2 Hz); ^13^C{^1^H} NMR (101 MHz, DMSO-*d*_6_, 75 °C) δ (ppm): 167.6, 164.5, 163.3, 156.1, 155.6, 152.6, 151.7, 147.5, 133.5, 128.9, 128.6, 126.2, 120.5 (q, *J* = 322 Hz), 116.5, 115.0, 113.2, 108.0, 105.8, 96.3, 68.4, 44.2, 42.4, 20., 12.0; ^19^F NMR (376.5 MHz, DMSO-*d*_6_): −77. 8 (3F, s); HRMS (ESI-TOF) *m*/*z* [M]^+^ calcd for C_29_H_29_N_4_O_3_^+^ 481.2234, found 481.2229; analytical HPLC (10 to 100% B over 30 min, column 2) *R*_t_ = 9.3 min.

##### Compound **7**

To a solution of coumarin **15** (33 mg, 0.063 mmol) in anhydrous ACN (5 mL), sodium hydride (60% dispersion in mineral oil, 7.6 mg, 0.19 mmol) was added and the resulting mixture was stirred for 15 min at room temperature under an Ar atmosphere. After the addition of 1-fluoro-2,4-dinitrobenzene (40 μL, 0.31 mmol), the reaction mixture was stirred overnight at 30 °C. Then, more NaH was added (5.0 mg, 0.13 mmol) since some starting material was still present according to HPLC-MS analysis, and the reaction mixture was stirred again overnight at 30 °C. After removal of the solvent under reduced pressure, the product was purified by column chromatography (silica gel, 50–100% DCM in hexanes, and then 2–25% MeOH in DCM) to give 7 mg (16% yield) of a purple solid. TLC: *R_f_* (10% MeOH in DCM) 0.28; ^1^H NMR (400 MHz, DMSO-*d*_6_) δ (ppm): 8.80 (1H, d, *J* = 2.4 Hz), 8.51 (2H, d, *J* = 7.6 Hz), 8.43 (1H, dd, *J* = 9.2, 3.0 Hz), 8.25 (2H, d, *J* = 7.6 Hz), 7.90 (1H, d, *J* = 9.6 Hz), 7.41 (1H, d, *J* = 9.6 Hz), 7.22 (1H, s), 7.01 (2H, m), 6.32 (1H, q, *J* = 6.4 Hz), 4.23 (3H, s), 3.62 (4H, q, *J* = 7.2 Hz), 1.86 (3H, d, *J* = 6.4 Hz), 1.28 (6H, t, *J* = 7.2 Hz); ^13^C{^1^H} NMR (101 MHz, DMSO-*d*_6_) δ (ppm): 166.4, 155.1, 153.6, 151.9, 151.7, 148.0, 144.4, 144.1, 140.4, 139.1, 129.4, 126.3, 121.9, 121.5, 121.3, 117.6, 116.5, 111.7, 106.4, 106.3, 97.0, 80.3, 74.0, 46.2, 44.2, 21.7, 12.4; HRMS (ESI-TOF) *m*/*z* [M + H]^+^ calcd for C_29_H_28_N_5_O_6_ 542.2034, found 542.2038 found; analytical HPLC (10 to 100% B over 30 min, column 2) *R*_t_ = 10.9 min.

##### Compound **8**

To a solution of coumarin **18** (27 mg, 0.051 mmol) in anhydrous ACN (5 mL), sodium hydride (60% dispersion in mineral oil, 6.12 mg, 0.15 mmol) was added under an Ar atmosphere. After stirring for 15 min at room temperature, 1-fluoro-2,4-dinitrobenzene (32 μL, 0.26 mmol) was added and the reaction mixture was stirred overnight at 30 °C. Then, more NaH was added (2.04 mg, 0.051 mmol) since some starting material was still present according to HPLC-MS analysis, and the reaction mixture was stirred for 3 h at 30 °C. After removal of the solvent under reduced pressure, the product was purified by column chromatography (silica gel, 0.25–10% MeOH in DCM) to give 11 mg (31% yield) of a purple solid. TLC: *R_f_* (10% MeOH in DCM) 0.27; ^1^H NMR (400 MHz, DMSO-*d*_6_) δ (ppm): 8.84 (1H, d, *J* = 3.2 Hz), 8.73 (1H, d, *J* = 7.2 Hz), 8.51 (1H, dd, *J* = 9.2, 2.8 Hz), 8.19 (1H, d, *J* = 7.2 Hz), 7.93 (1H, d, *J* = 8.8 Hz), 7.60 (1H, d, *J* = 9.6 Hz), 7.10 (1H, s), 7.01 (2H, m), 6.52 (1H, q, *J* = 6.8 Hz), 4.46 (2H, t, *J* = 7.2 Hz), 3.58 (4H, 1, *J* = 7.2 Hz), 1.87 (2H, m), 1.73 (3H, d, *J* = 6.4 Hz), 1.29 (6H, m), 1.19 (6H, t, *J* = 7.2 Hz), 0.86 (3H, m). ^13^C{^1^H} NMR (125 MHz, CDCl_3_) δ (ppm): 167.4, 156.0, 154.6, 152.9, 151.6, 149.4, 142.9, 140.9, 139.3, 129.5, 125.8, 122.5, 122.2, 117.8, 116.4, 112.2, 107.5, 106.5, 99.1, 81.7, 75.9, 60.3, 53.6, 45.7, 31.5, 31.2, 26.0, 22.5, 22.2, 14.0, 12.8. HRMS (ESI-TOF) *m*/*z* [M + H]^+^ calcd for C_34_H_38_N_5_O_6_ 612.2817, found 612.2817; analytical HPLC (10 to 100% B over 30 min, column 2) *R*_t_ = 9.5 min.

### Photophysical Characterization of COUPY-Caged Compounds (**3**–**8**)

The absorption spectra were recorded in a Jasco V-730 spectrophotometer at room temperature. Molar absorption coefficients (ε) were determined by direct application of the Beer–Lambert law using solutions of the compounds in a 1:1 (v/v) mixture of PBS buffer and ACN with concentrations about 10^–6^ M. The emission spectra were registered in a Photon Technology International (PTI) fluorimeter. Fluorescence quantum yields (Φ_F_) were measured by the comparative method using cresyl violet in ethanol (CV; Φ_F;Ref_ = 0.54 ± 0.03) as a reference.^26^ Then, optically matched solutions of the compounds and CV were excited and the fluorescence spectra were recorded. The absorbance of sample and reference solutions was set below 0.1 at the excitation wavelength (540 nm), and Φ_F_ values were calculated using eq 1:
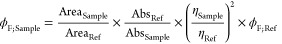
1where Area_Sample_ and Area_Ref_ are the integrated fluorescence for the sample and the reference, Abs_Sample_ and Abs_Ref_ are the absorbance registered at 540 nm, and η_Sample_ and η_Ref_ are the refractive indexes of sample and reference solutions, respectively.

### Irradiation Experiments

Photolysis studies were performed at 37 °C in a custom-built irradiation setup from Microbeam, which includes a high-performance quartz glass cuvette, a thermostated cuvette holder, and mounted high-power light-emitting diodes (LEDs) from BWTEK Inc. of red (620 ± 15 nm; 130 mW cm^–2^) and wide range (470–750 nm range, centered at 530 nm; 150 mW cm^–2^) light (Figure S12). The incorporation of a bandpass filter in the visible LED provided yellow light with a maximum emission wavelength around 560 ± 40 nm (40 mW cm^–2^) (Figure S12). In a typical experiment, the cuvette containing 1.5 mL of a solution of the caged compound (20 μM) and 4-*N*,*N′*-dimethylaminopyridine (internal standard, 20 μM) in a 1:1 (v/v) mixture of PBS buffer and ACN was placed in front of the light source (distance <0.1 mm) and irradiated for the indicated times while constantly stirred. Light irradiance at the cuvette was measured by using a light meter and used to calculate the photon irradiance spectra using the emission spectra of the LEDs. Then, the rate of photon absorption by the sample was calculated by multiplying the photon irradiance spectra by the absorption factor of the sample at each wavelength (1–10^–*A*(λ)^, where *A*(λ) is the sample absorbance) and integrating over the entire spectrum. At each time point, samples were taken and analyzed by reversed-phase HPLC-ESI-MS with a Jupiter Proteo C_18_ column (250 × 4.6 mm, 90 Å, 4 μm, flow rate: 1 mL min^–1^) by using linear gradients of 0.1% formic acid in H_2_O (A) and 0.1% formic acid in ACN (B). Photolysis quantum yields were calculated as the initial slope of the plot of the amount of coumarin deprotected vs the number of photons absorbed.^20^ Only the initial points were included in the calculation to avoid inner-filter effects due to the photoproducts, which absorb in the same range and thus slow down the process as the reaction progresses.

### Confocal Microscopy Studies

#### Cell Culture and Treatments

HeLa cells were maintained in DMEM containing high glucose (4.5 g/L) and supplemented with 10% FBS and 50 U/mL penicillin–streptomycin. For cellular uptake experiments and posterior observation under the microscope, cells were seeded on glass-bottom dishes (P35G-1.5-14-C, MatTek). Twenty-four hours after cell seeding, cells were incubated for 30 min at 37 °C with the compounds (**7**, **8**, **15**, or **18**, 2 μM; Rho123 200 μM) in supplemented DMEM. Then, cells were washed two times with DPBS (Dulbecco’s phosphate-buffered saline, pH 7.0–7.3) to remove the excess of the fluorophores and kept in low-glucose DMEM without phenol red for fluorescence imaging.

For co-localization experiments with MitoTracker Green FM, HeLa cells were treated with compounds **8** or **18** (2 μM) and MitoTracker Green FM (0.1 μM) for 30 min at 37 °C in non-supplemented DMEM. After removal of the medium and washing two times with DPBS, cells were kept in low-glucose DMEM without phenol red for fluorescence imaging.

#### Fluorescence Imaging

All microscopy observations were performed using a Zeiss LSM 880 confocal microscope equipped with a 405 nm laser diode, an argon-ion laser, a 561 nm laser, and a 633 nm laser. The microscope was also equipped with a Heating Insert P S (Pecon) and a 5% CO_2_-providing system. Cells were observed at 37 °C using a 63× 1.4 oil immersion objective. Compounds **7**, **8**, **15**, and **18** were excited using the 561 nm laser and detected from 570 to 670 nm. Rho123 and MTG were observed using the 488 nm laser line of the argon-ion laser, whereas the 405 nm laser diode was used for observing Hoechst 33342. Irradiation experiments were also performed in the confocal microscope by using its fluorescence filter set 43 with an excitation BP 545/25 filter and its HXP 120 V fluorescence lamp at 1.4 mW/cm^2^ for 15 s. Image processing and analysis were performed using Fiji.^27^

#### Intensity Measurement

The compound and Rho123 images were processed by background subtraction (rolling ball radius = 50) and median filtering (radius = 2). Mean intensity was measured after setting the Huang threshold.^28^

#### Co-Localization Coefficients

The MitoTracker and compound channels were processed by median filtering (radius = 1), Gaussian filtering (sigma = 1), and background subtraction (rolling ball radius = 30). Then, images were segmented by applying the Li threshold,^29^ and the resulting binary images were used to mask the original images. Co-localization coefficients were measured using the JaCoP plugin17 on the different stacks of images (*n* = 5) with each stack containing 25 cells on average.

## Data Availability

The data underlying this study are available in the published article and its Supporting Information.

## Supporting Information Available

UV–vis absorption and fluorescence emission spectra of the compounds, additional figures and material from stability studies, irradiation experiments and fluorescence imaging, and copies of ^1^H and ^13^C{^1^H} NMR and HRMS spectra of the synthesized compounds (PDF)
