# Supplementary material for: Mitochondria-Targeted COUPY Photocages: Synthesis and Visible-Light Photoactivation in Living Cells
Source: J Org Chem. 2023 May 20;88(11):7128–40. doi: 10.1021/acs.joc.3c00387 (PMC10242765; doi:10.1021/acs.joc.3c00387)
Supplement: Supplementary file 1 — jo3c00387_si_001.pdf [file jo3c00387_si_001.pdf]

SUPPORTING INFORMATION

Mitochondria-targeted COUPY photocages: synthesis and visible-light photoactivation in living cells.

Marta López-Corrales,[1] Anna Rovira,[1] Albert Gandioso,[1] Santi Nonell,[2] Manel Bosch,[3] Vicente Marchán*,[1]

[1] Departament de Química Inorgànica i Orgànica, Secció de Química Orgànica, Institut de Biomedicina de la Universitat de Barcelona (IBUB), Universitat de Barcelona (UB), Martí i Franqués 1–11, E-08028 Barcelona (Spain).

[2] Institut Químic de Sarrià, Universitat Ramon Llull, Vía Augusta 390, E-08017 Barcelona (Spain)

[3] Unitat de Microscòpia Òptica Avançada, Centres Científics i Tecnològics (CCiTUB), Universitat de Barcelona (UB), Av. Diagonal 643, E- 08028 Barcelona (Spain)

E-mail: vmarchan@ub.edu

Table of contents

1.- Reversed-phase HPLC analysis of COUPY-caged compounds (**3-8**) S3

2.- 2D NMR characterization of coumarin scaffolds (**12-14, 17 and 18)** S4

3.- 2D NMR characterization of COUPY-caged compounds (**4-8**) S9

4.- Irradiation experiments S14

5.- Confocal microscopy studies S26

6.- ^{1}H, ^{13}C and ^{19}F NMR spectra and HR ESI-MS of the compounds S32

1.- Reversed-phase HPLC analysis of COUPY-caged compounds (3-8).

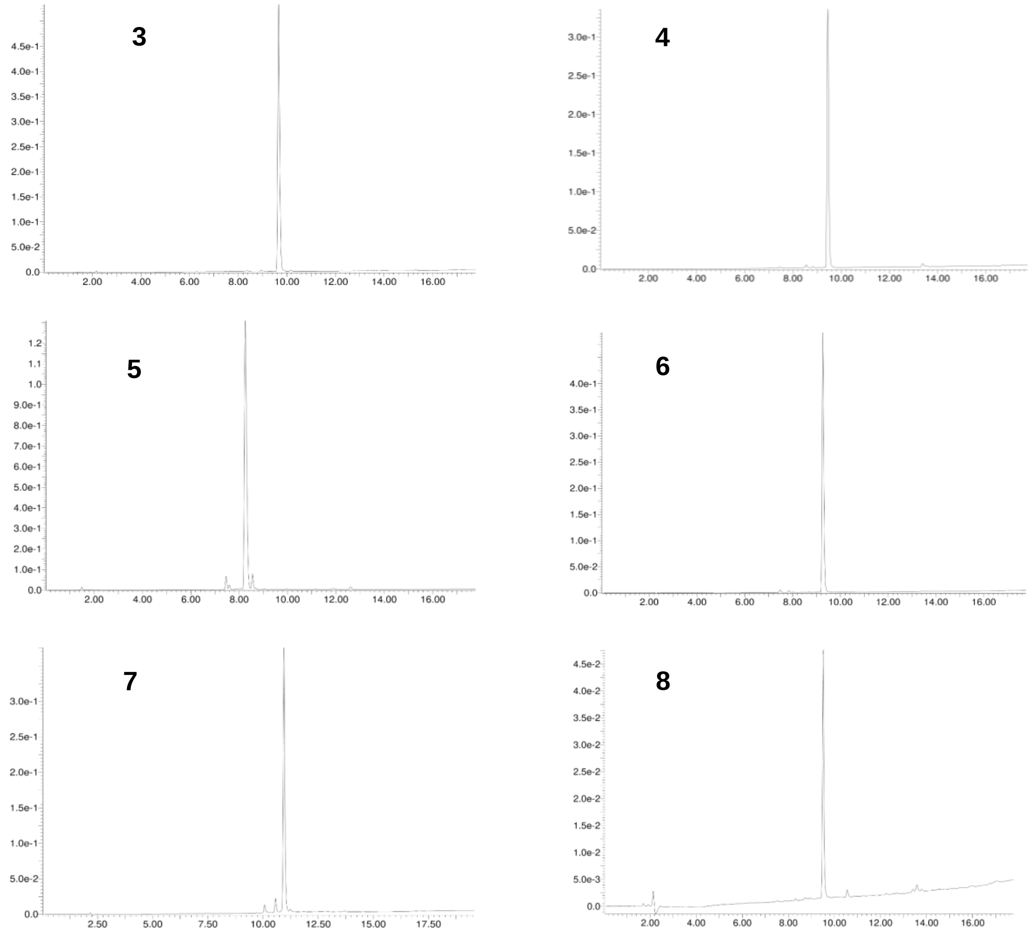

Figure S1. Reversed-phase HPLC traces of COUPY-caged compounds (**3-8**).

2.- 2D NMR characterization of coumarin scaffolds (12-14, 17 and 18).

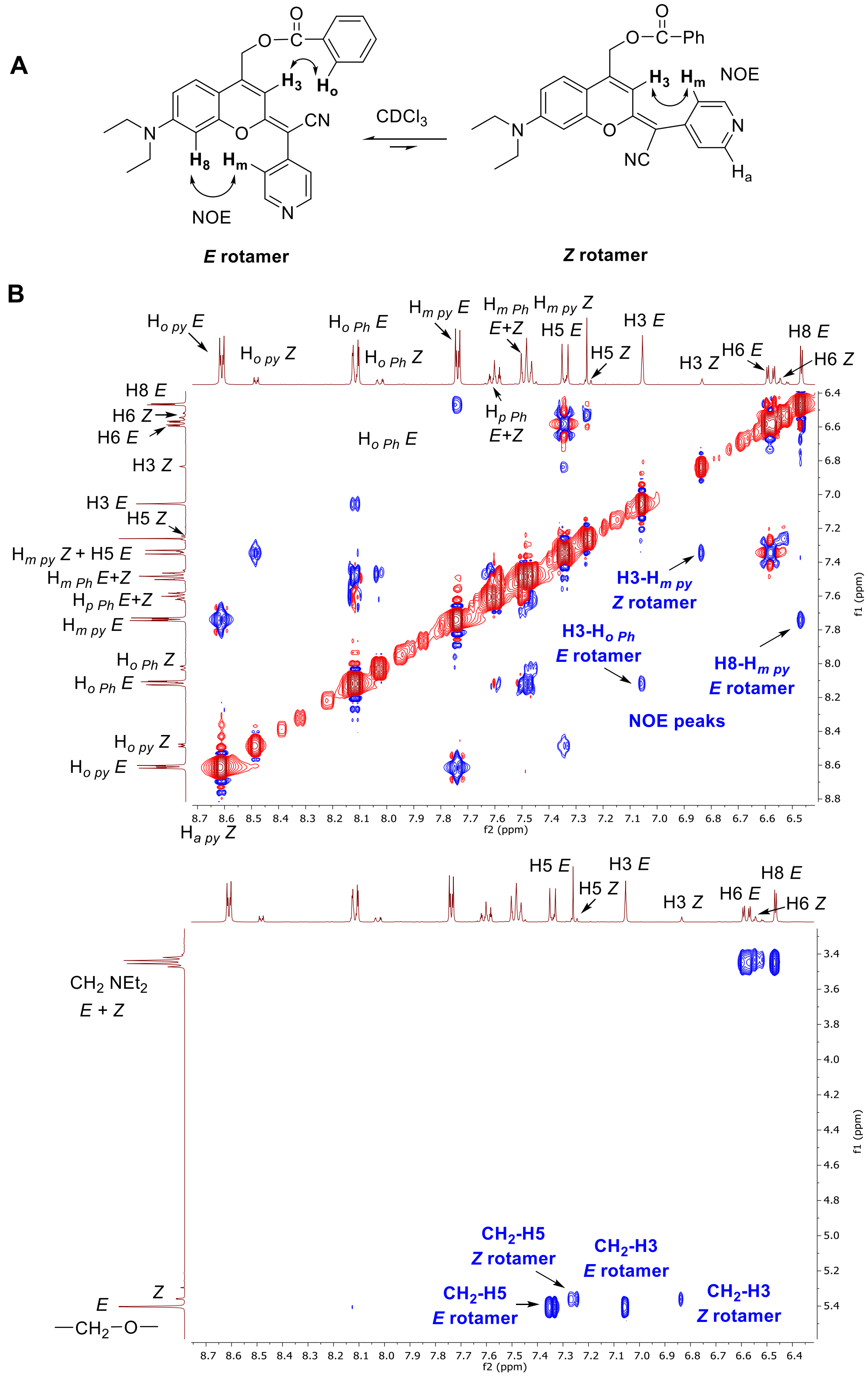

Figure S2. Structure of *E* and *Z* rotamers of coumarin **12** with some diagnostic NOE cross-peaks indicated, and expansions of the NOESY spectrum of **12** in $CDCl_3$ at 298 K showing some characteristic NOE cross-peaks.

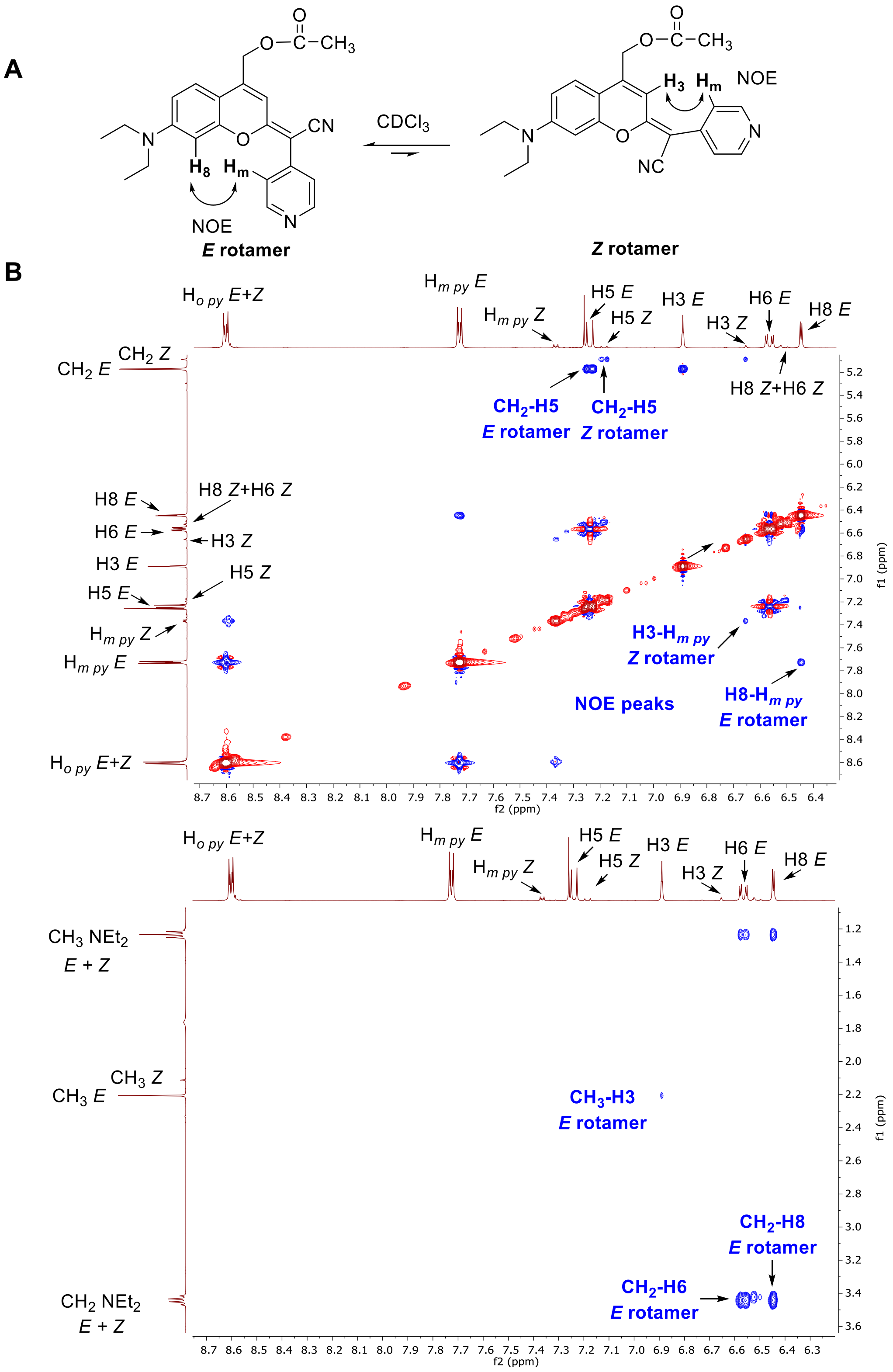

Figure S3. Structure of *E* and *Z* rotamers of coumarin **13** with some diagnostic NOE cross-peaks indicated, and expansions of the NOESY spectrum of **13** in $CDCl_3$ at 298 K showing some characteristic NOE cross-peaks.

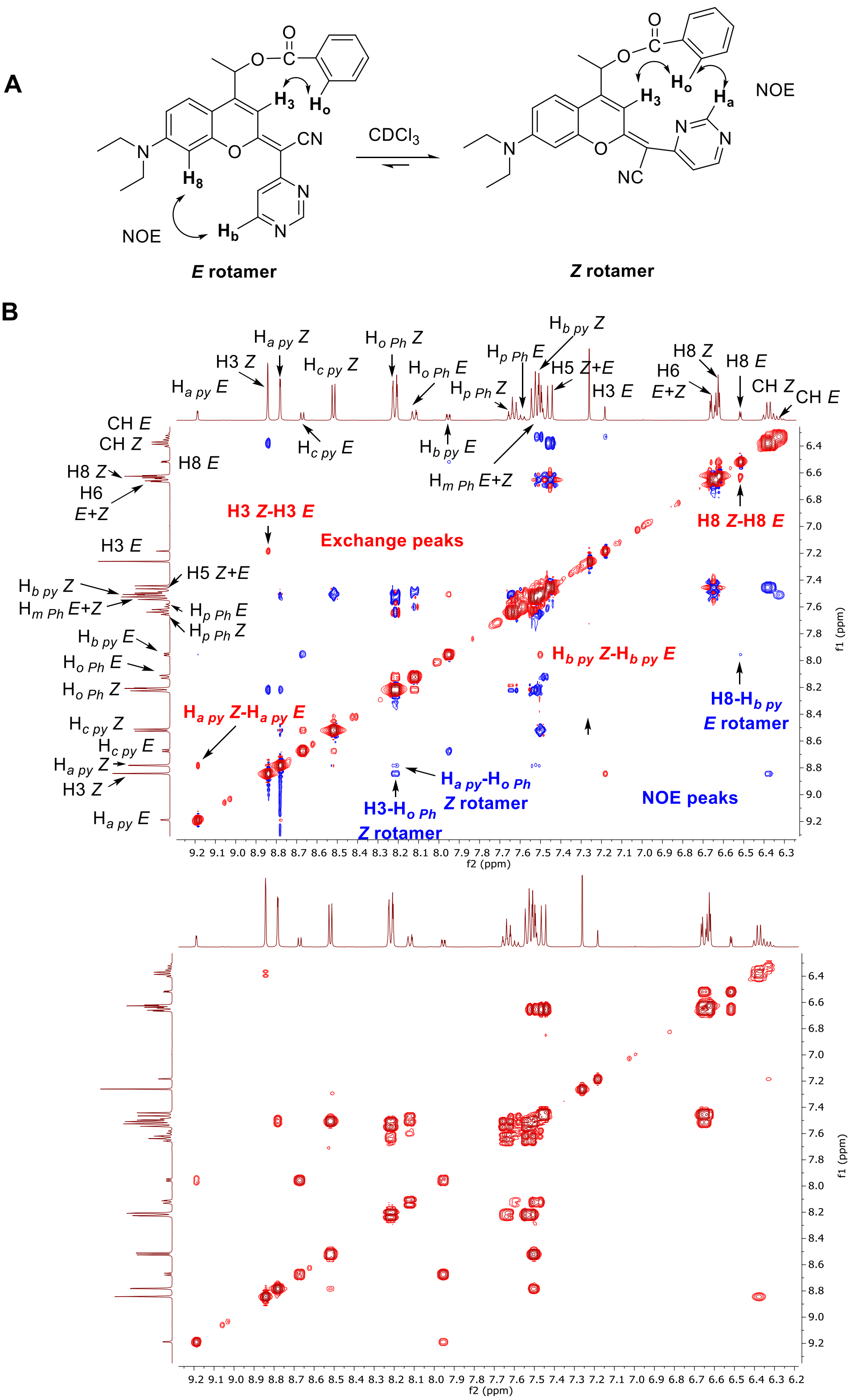

Figure S4. Structure of *E* and *Z* rotamers of coumarin **14** with some diagnostic NOE cross-peaks indicated, and expansions of the NOESY spectrum showing some characteristic NOE cross-peaks and exchange cross-peaks between rotamer resonances of the same sign as the diagonal (top) and of the COSY spectrum (bottom) of **14** in $CDCl_3$ at 298 K.

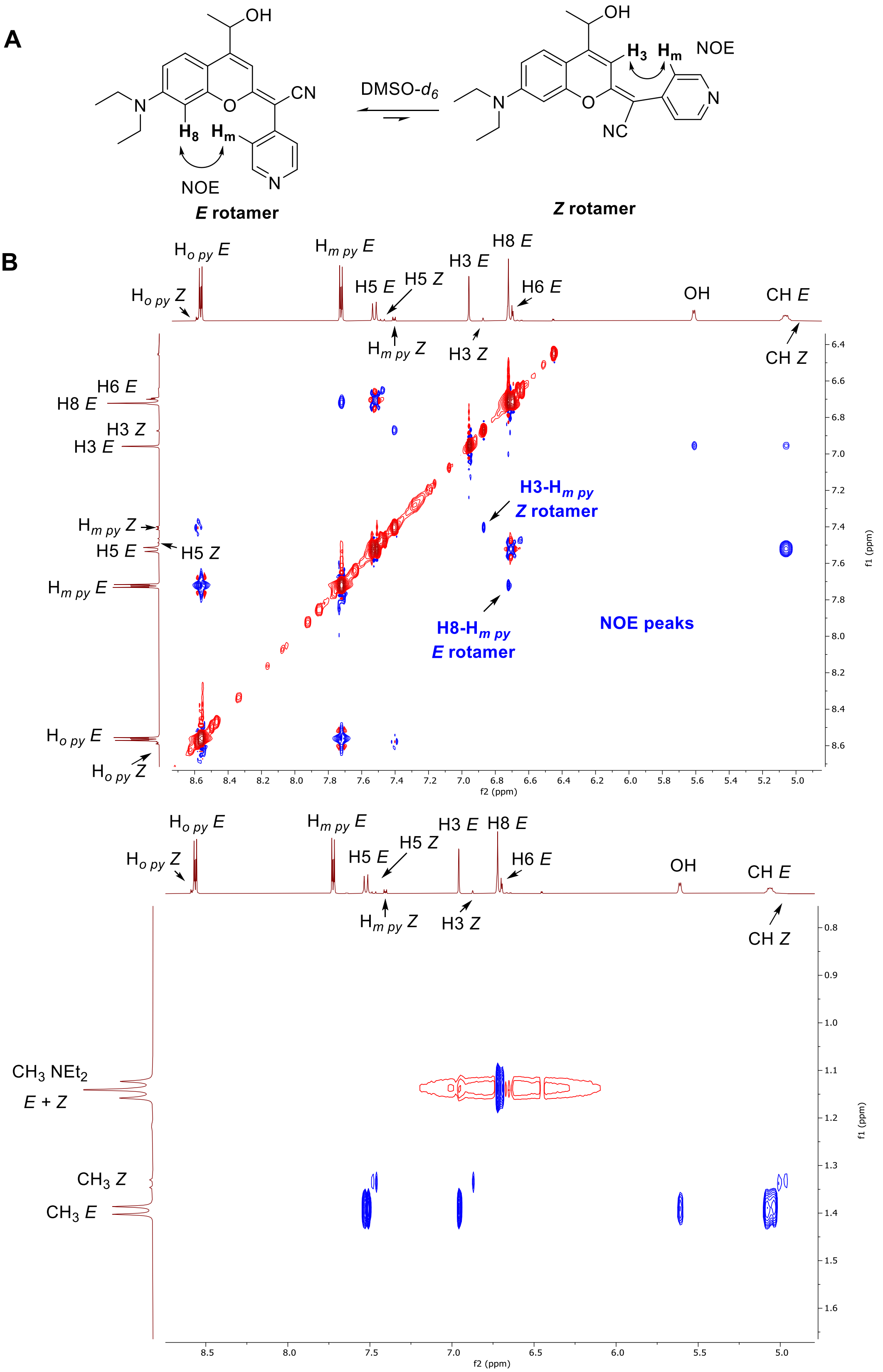

Figure S5. Structure of *E* and *Z* rotamers of coumarin **17** with some diagnostic NOE cross-peaks indicated, and expansions of the NOESY spectrum of **17** in DMSO-d_6 at 298 K showing some characteristic NOE cross-peaks.

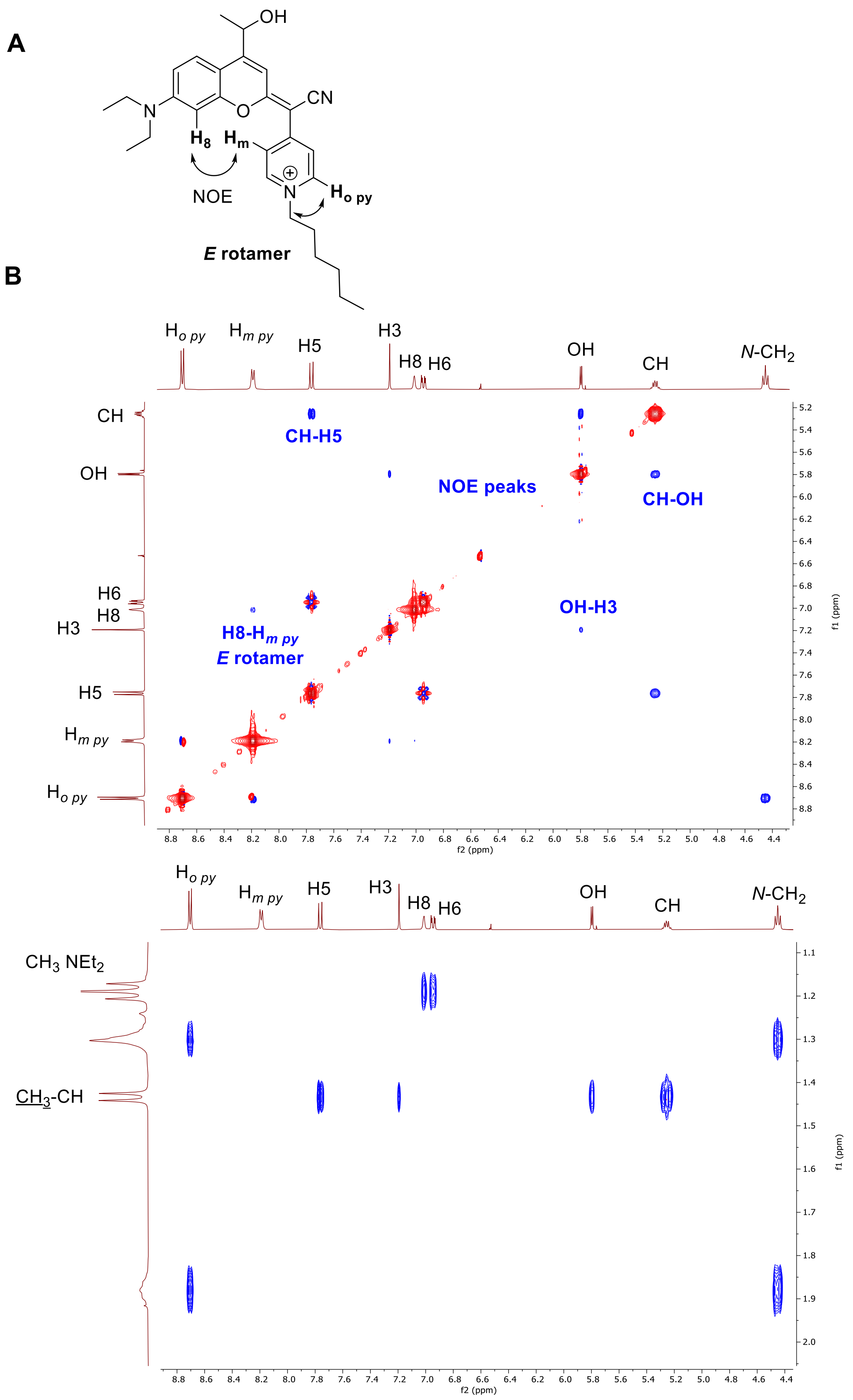

Figure S6. Structure of *E* rotamer of coumarin **18** with some diagnostic NOE cross-peaks indicated, and expansions of the NOESY spectrum of **18** in DMSO-d_6 at 298 K showing some characteristic NOE cross-peaks.

3.- 2D NMR characterization of COUPY-caged compounds (4-8).

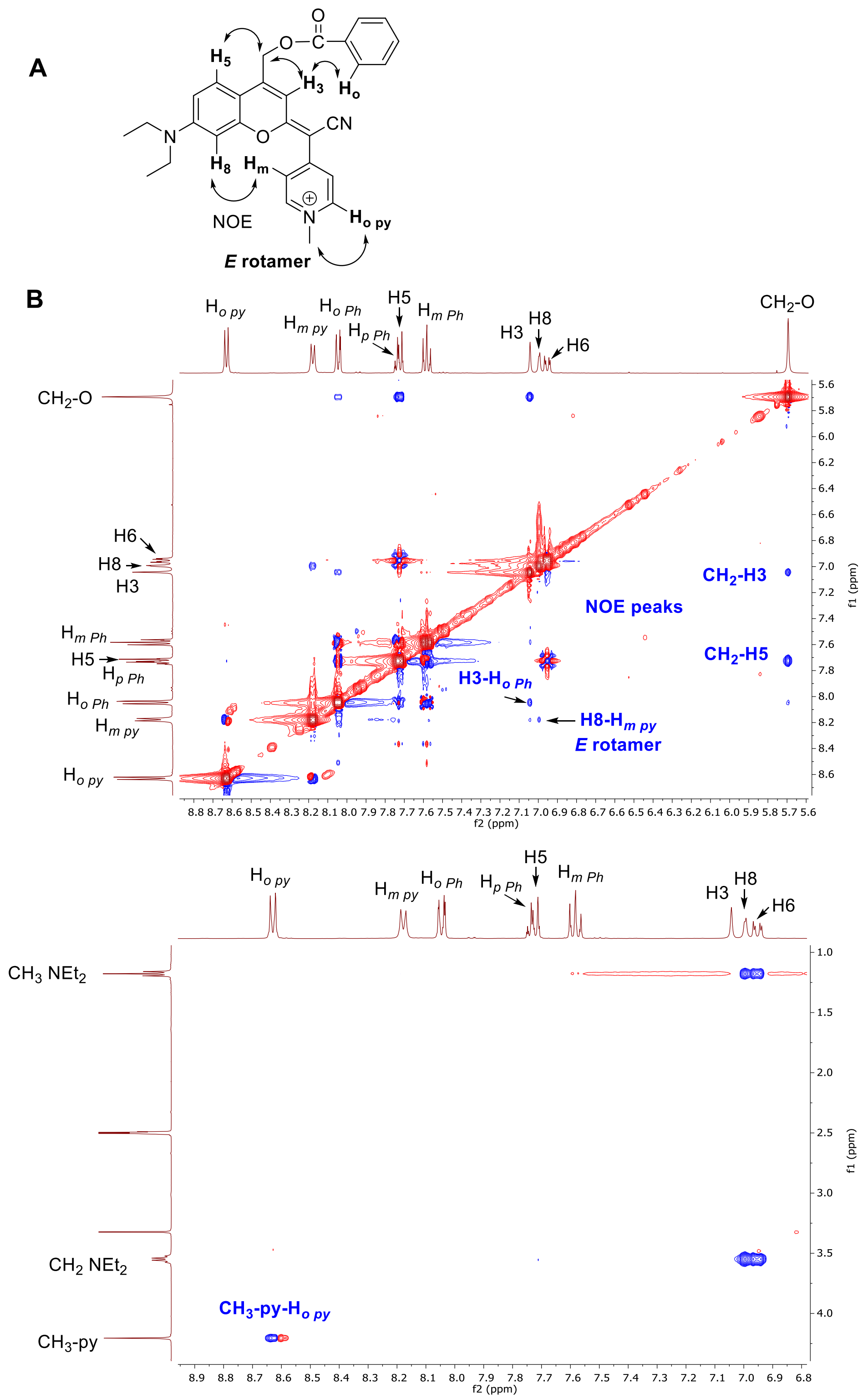

Figure S7. Structure of *E* rotamer of COUPY-caged compound **4** with some diagnostic NOE cross-peaks indicated, and expansions of the NOESY spectrum of **4** in DMSO-d_6 at 298 K showing some characteristic NOE cross-peaks.

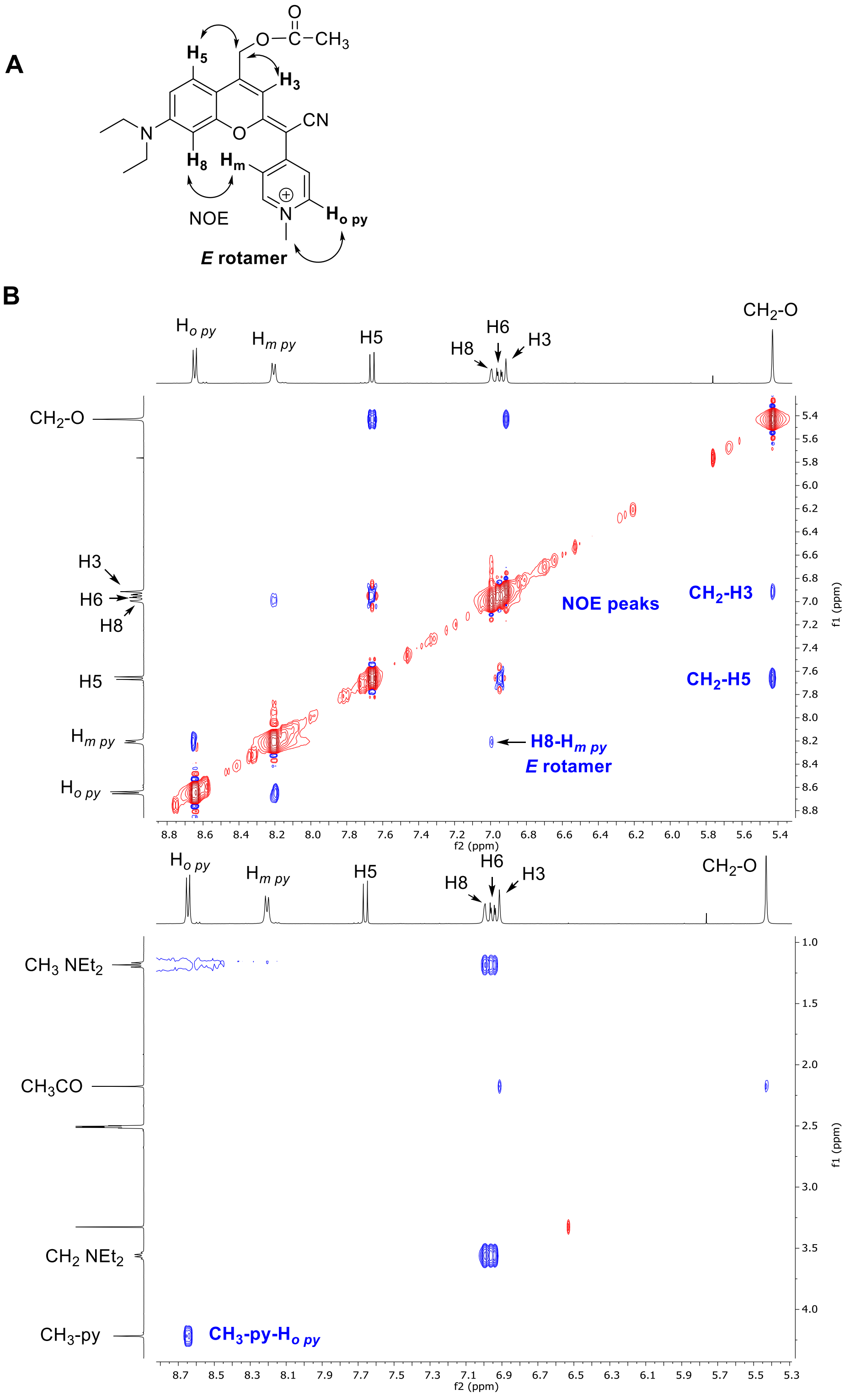

Figure S8. Structure of *E* rotamer of COUPY-caged compound **5** with some diagnostic NOE cross-peaks indicated, and expansions of the NOESY spectrum of **5** in DMSO-d_6 at 298 K showing some characteristic NOE cross-peaks.

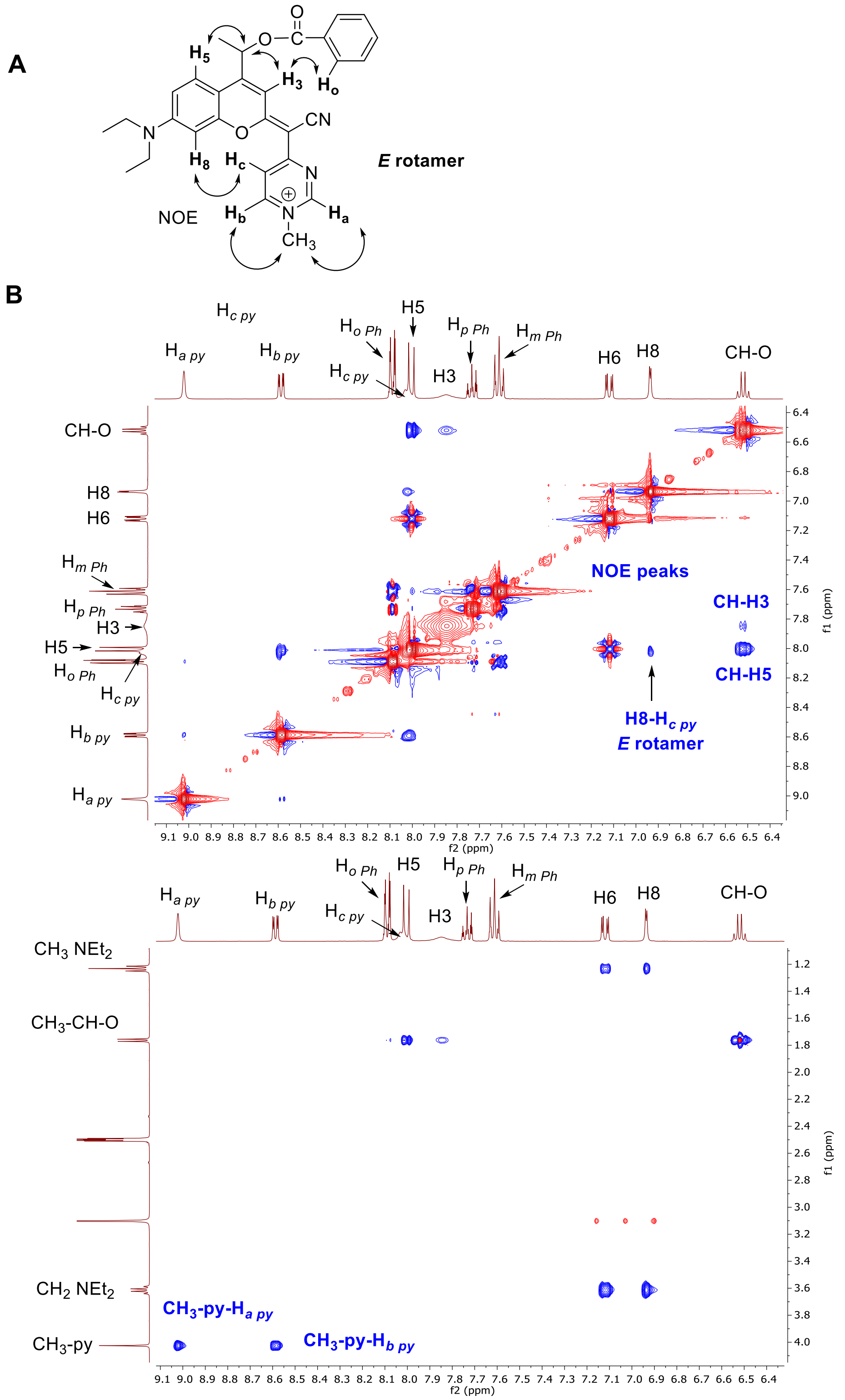

Figure S9. Structure of *E* rotamer of COUPY-caged compound **6** with some diagnostic NOE cross-peaks indicated, and expansions of the NOESY spectrum of **6** in DMSO-d_6 at 348 K showing some characteristic NOE cross-peaks.

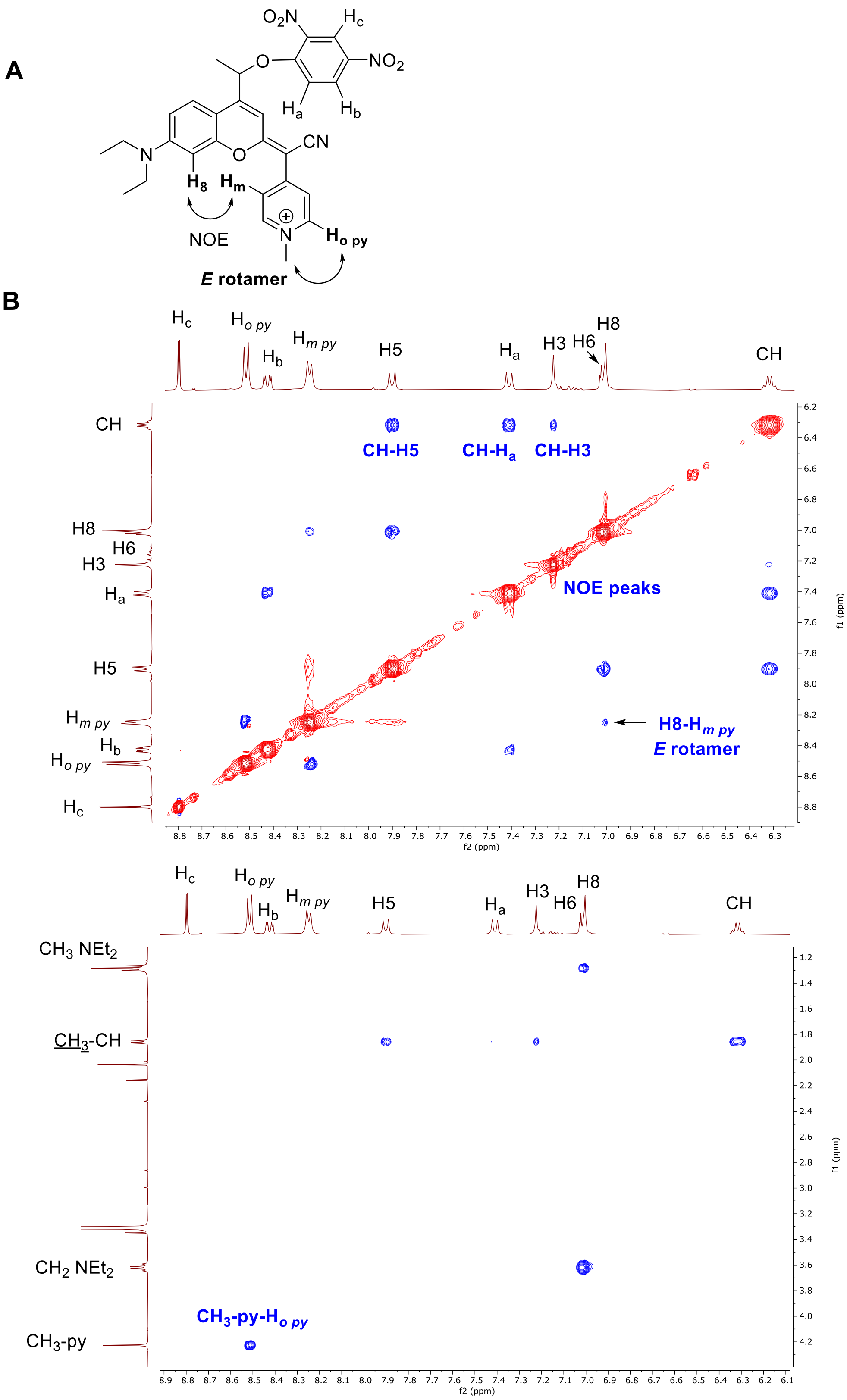

Figure S10. Structure of *E* rotamer of COUPY-caged compound **7** with some diagnostic NOE cross-peaks indicated, and expansions of the NOESY spectrum of **7** in CD_3OD at 298 K showing some characteristic NOE cross-peaks.

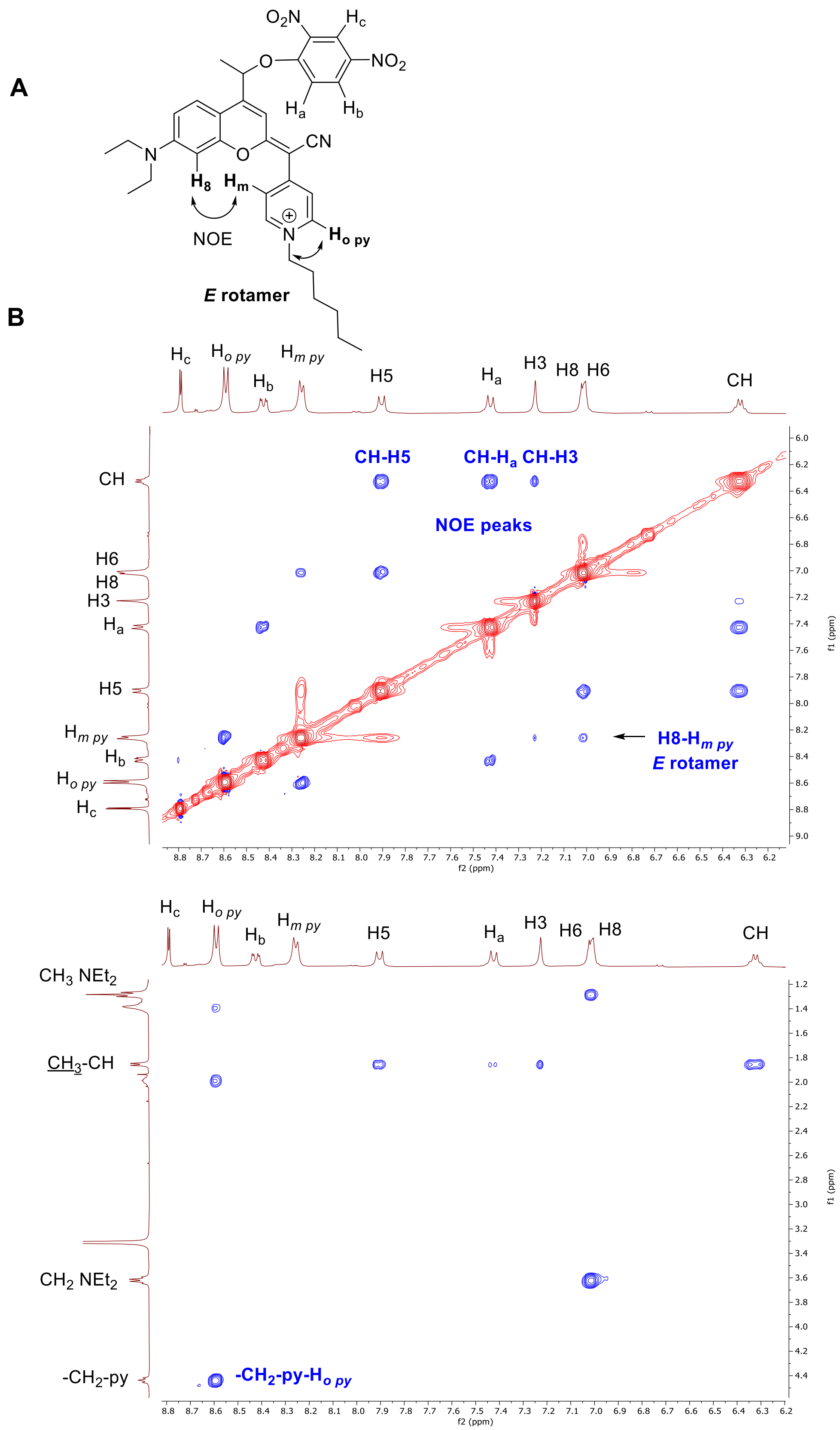

Figure S11. Structure of *E* rotamer of COUPY-caged compound **8** with some diagnostic NOE cross-peaks indicated, and expansions of the NOESY spectrum of **8** in CD_3OD at 298 K showing some characteristic NOE cross-peaks.

4.- Irradiation experiments.

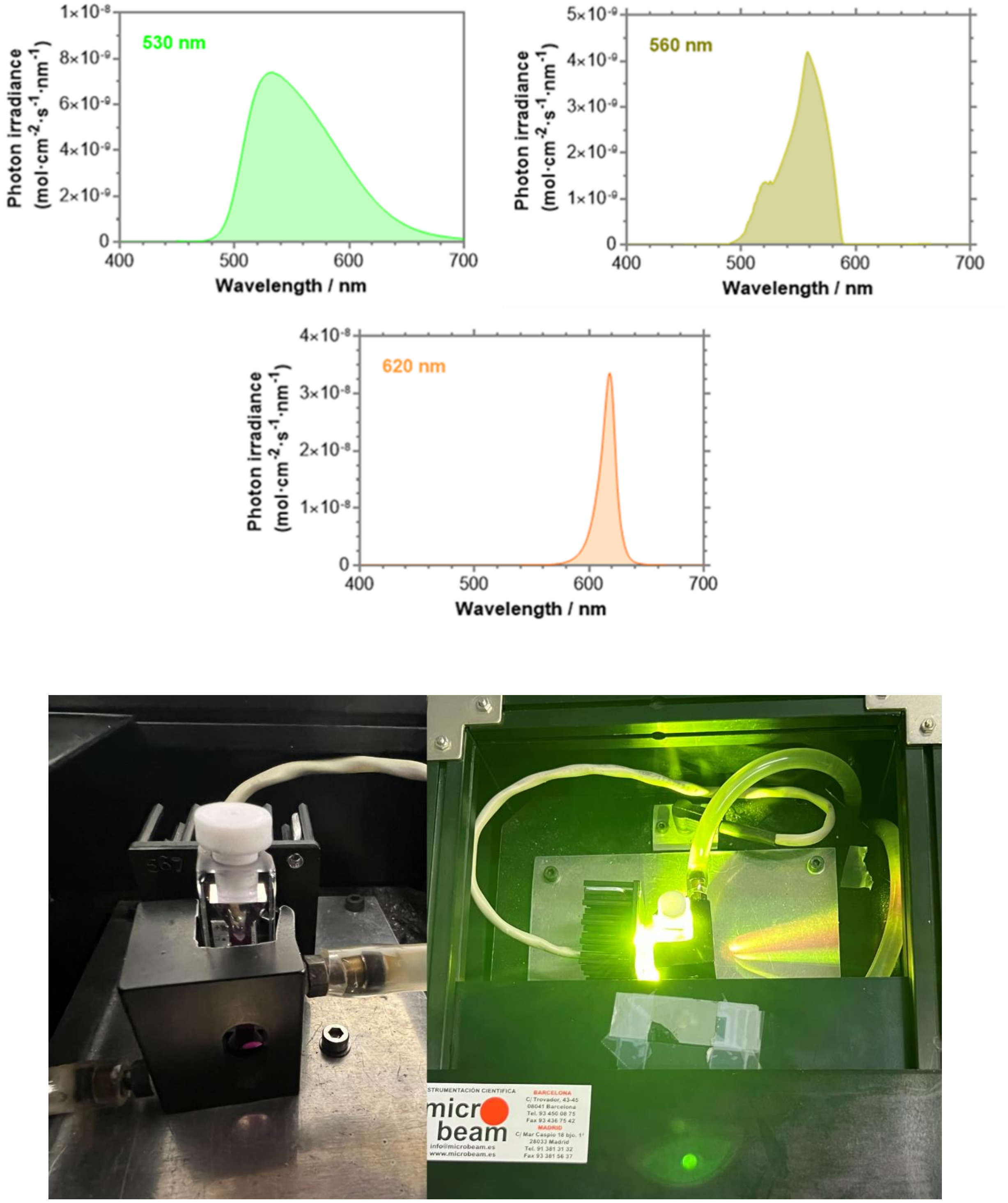

Figure S12. Photon spectral irradiance of the LEDs used in this work (top) and photographs of the custom-built irradiation setup used for uncaging experiments (bottom).

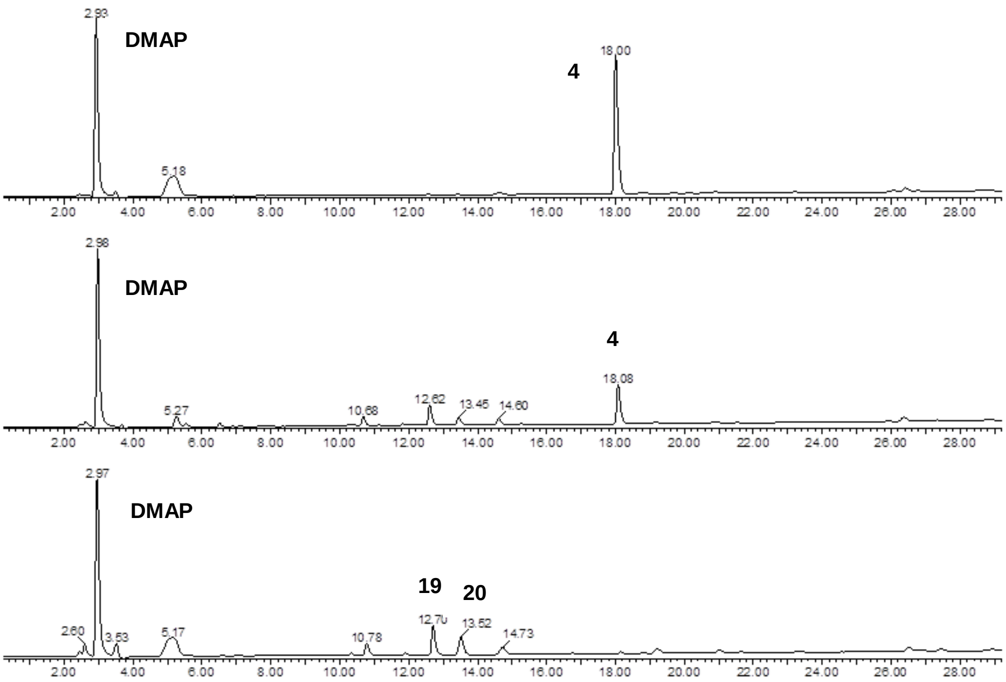

Figure S13. Reversed-phase HPLC-ESI MS traces at 260 nm for the photolysis reaction of **4** in a 1:1 (v/v) mixture of PBS buffer and ACN in the presence of DMAP (internal standard) at t=0 (top) and after irradiation with visible LED light (470-750 nm range, centered at 530 nm) for 15 min (middle) and 60 min (bottom) at 37 ºC.

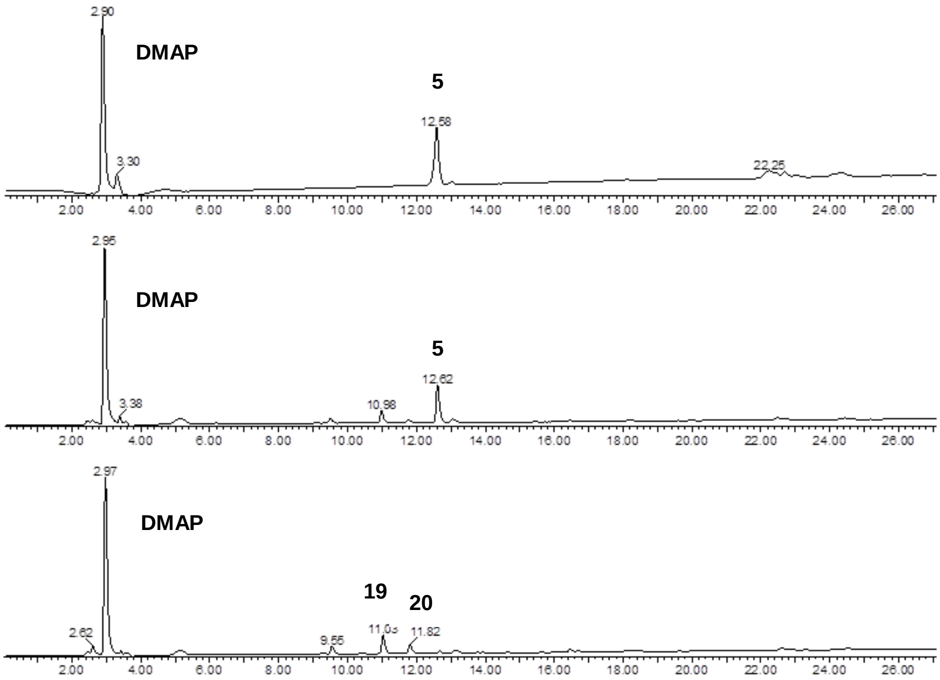

Figure S14. Reversed-phase HPLC-ESI MS traces at 260 nm for the photolysis reaction of **4** in a 1:1 (v/v) mixture of PBS buffer and ACN in the presence of DMAP (internal standard) at t=0 (top) and after irradiation with visible LED light (470-750 nm range, centered at 530 nm) for 15 min (middle) and 60 min (bottom) at 37 ºC.

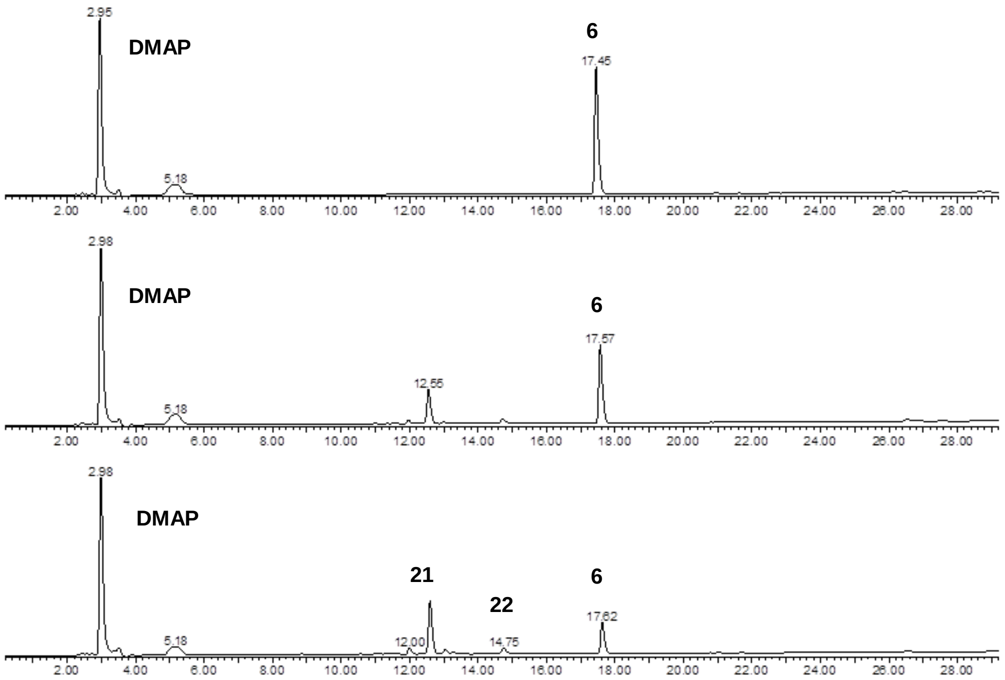

Figure S15. Reversed-phase HPLC-ESI MS traces at 260 nm for the photolysis reaction of **6** in a 1:1 (v/v) mixture of PBS buffer and ACN in the presence of DMAP (internal standard) at t=0 (top) and after irradiation with visible LED light (470-750 nm range, centered at 530 nm) for 30 min (middle) and 60 min (bottom) at 37 ºC.

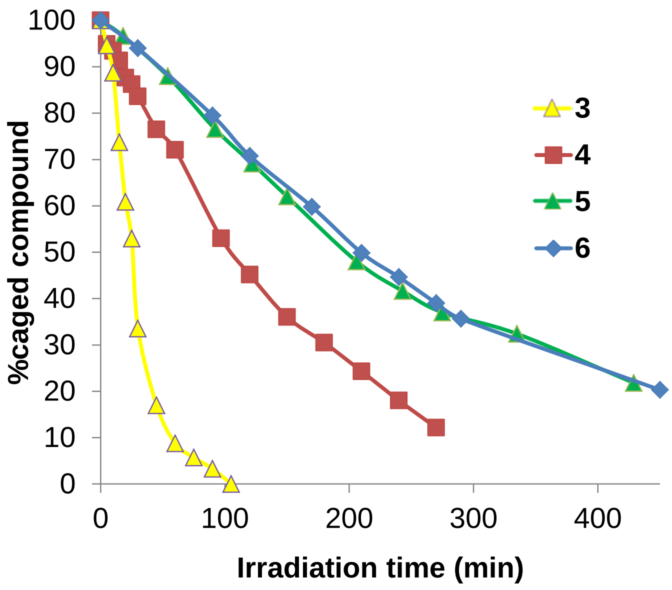

Figure S16. Plot of the temporal evolution of the amounts of COUPY-caged compounds **3-6** after irradiation with yellow light (560 ± 40 nm; 40 mW cm^{-2}). The lines connecting the experimental points are meant to aid the reader in visualizing the data. All the experiments were performed in a 1:1 (v/v) mixture of PBS buffer and ACN at 37 ºC.

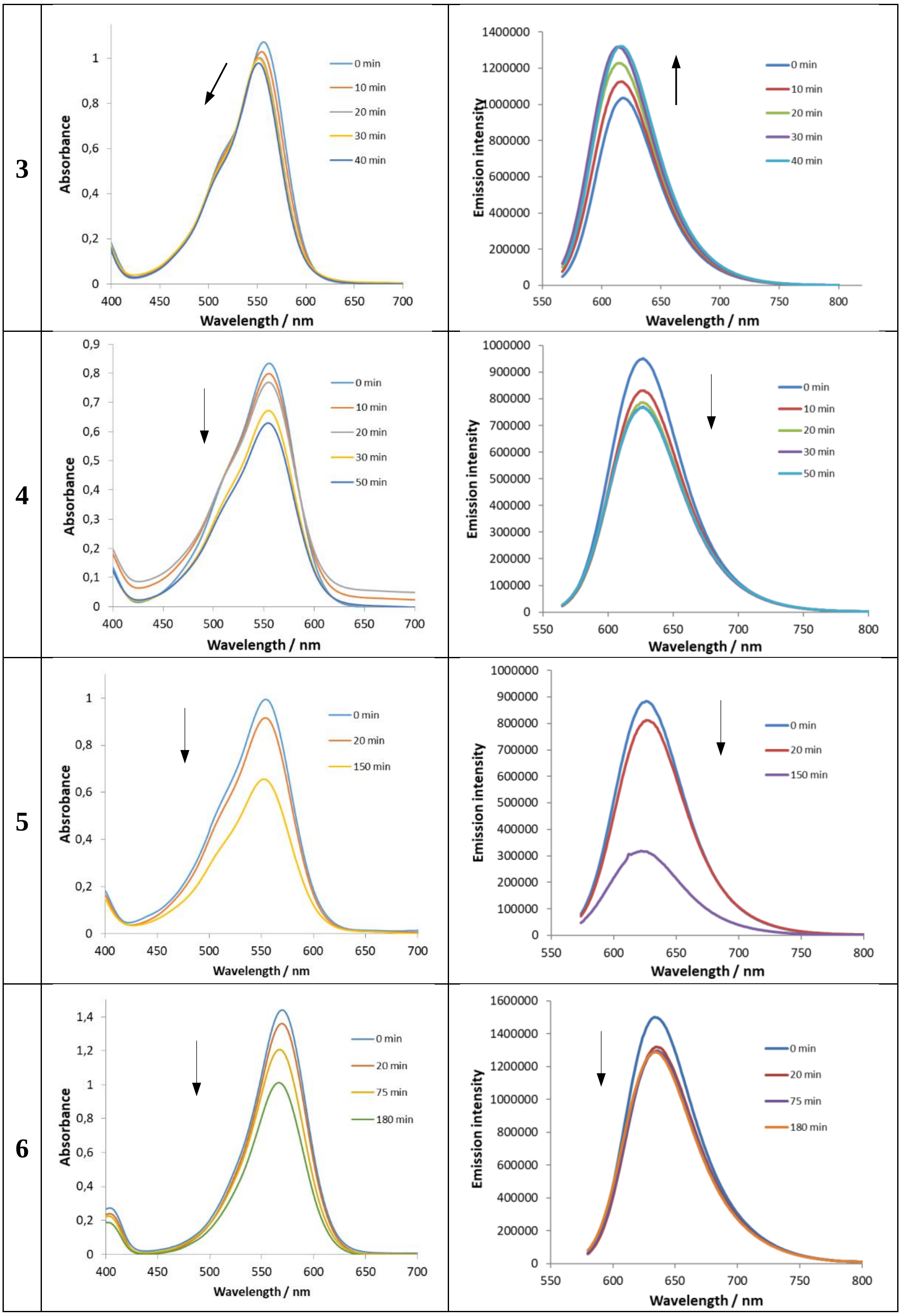

Figure S17. Temporal evolution of the solution absorbance and emission intensity of COUPY caged compounds **3-6** upon illumination with a LED source covering from the cyan to the far-red region of the visible spectrum (470–750 nm; 150 mW cm^{-2}). All the experiments were performed in a 1:1 (v/v) mixture of PBS buffer and ACN at 37 °C.

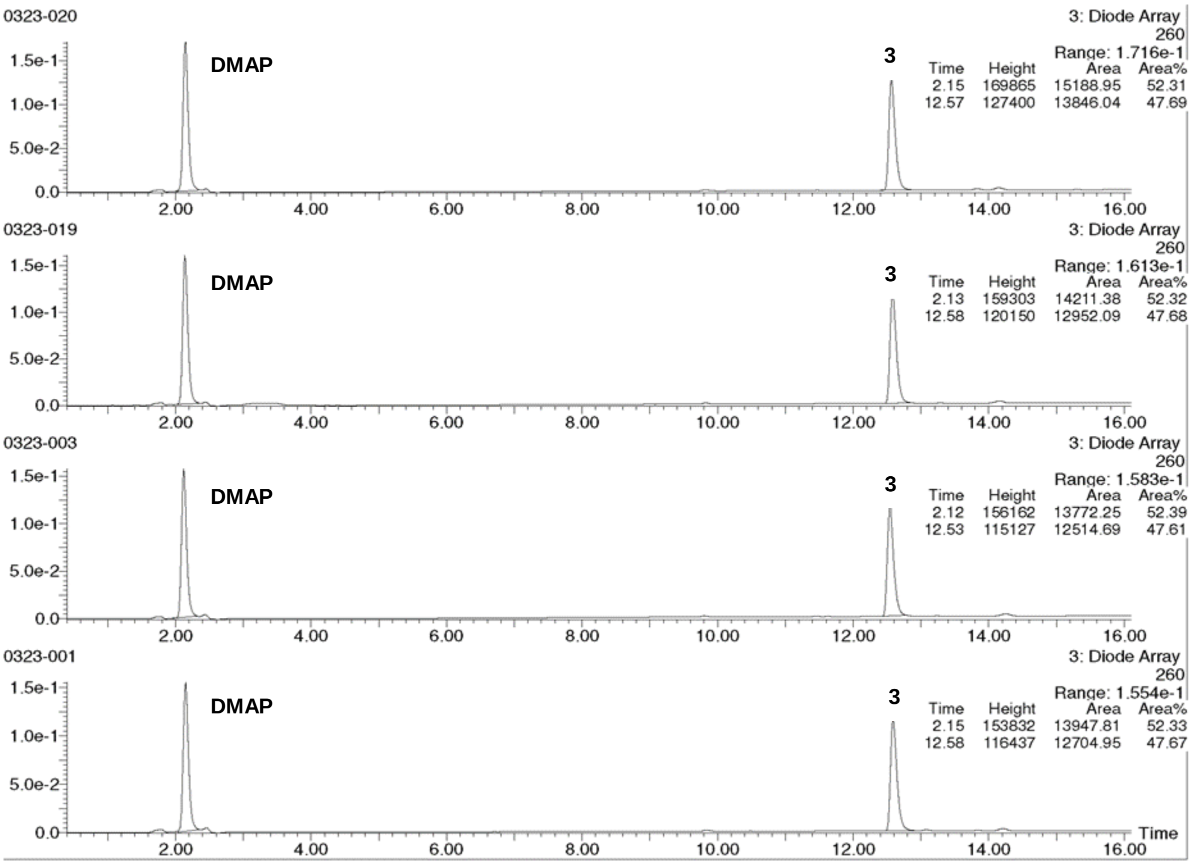

Figure S18. Reversed-phase HPLC-ESI MS traces at 260 nm of COUPY-caged compound **3** after standing for different times in the dark at 37 ºC in a 1:1 (v/v) mixture of PBS buffer and ACN in the presence of DMAP (internal standard). From bottom to top: t= 0, 2 h, 4 h and 5 h.

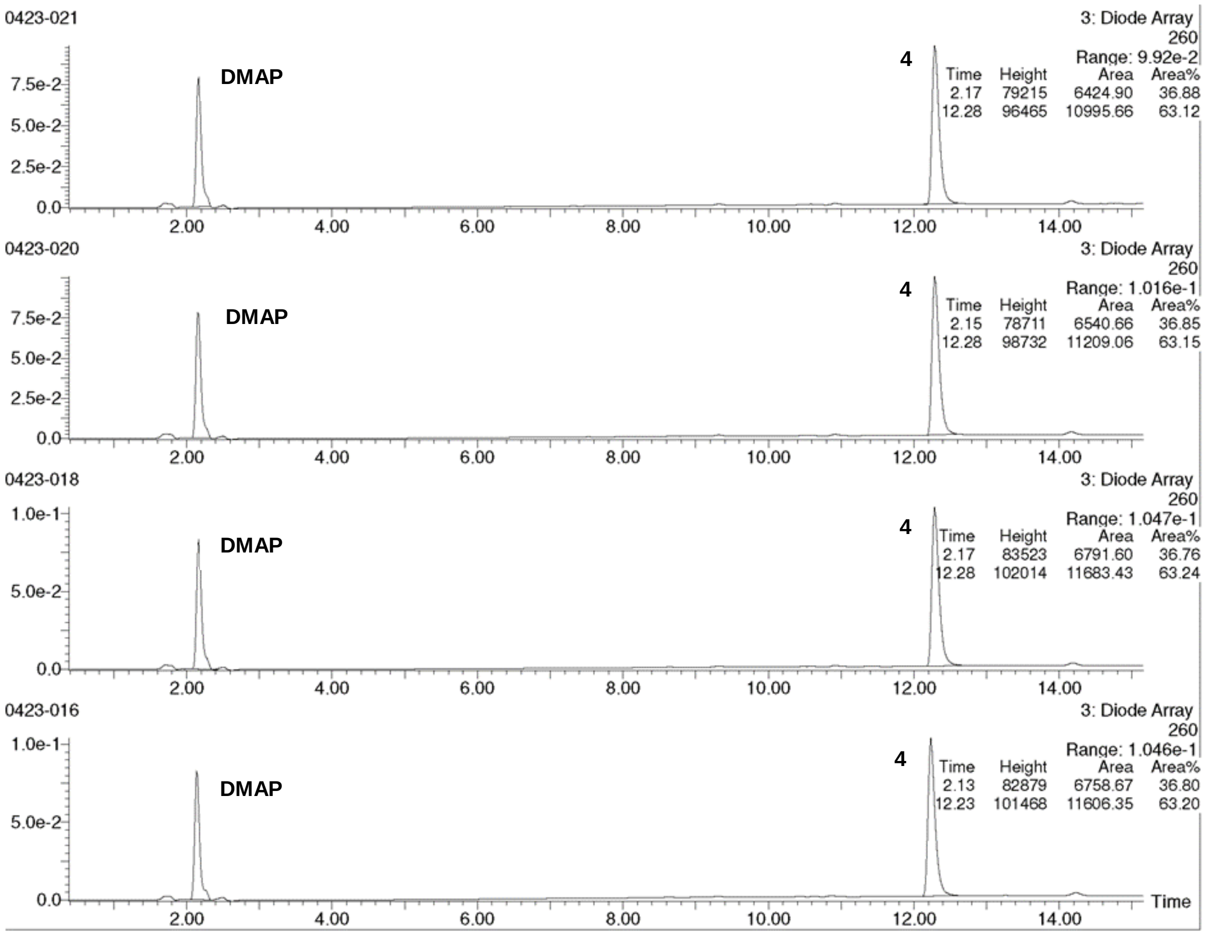

Figure S19. Reversed-phase HPLC-ESI MS traces at 260 nm of COUPY-caged compound **4** after standing for different times in the dark at 37 ºC in a 1:1 (v/v) mixture of PBS buffer and ACN in the presence of DMAP (internal standard). From bottom to top: t= 0, 2 h, 4 h and 5 h.

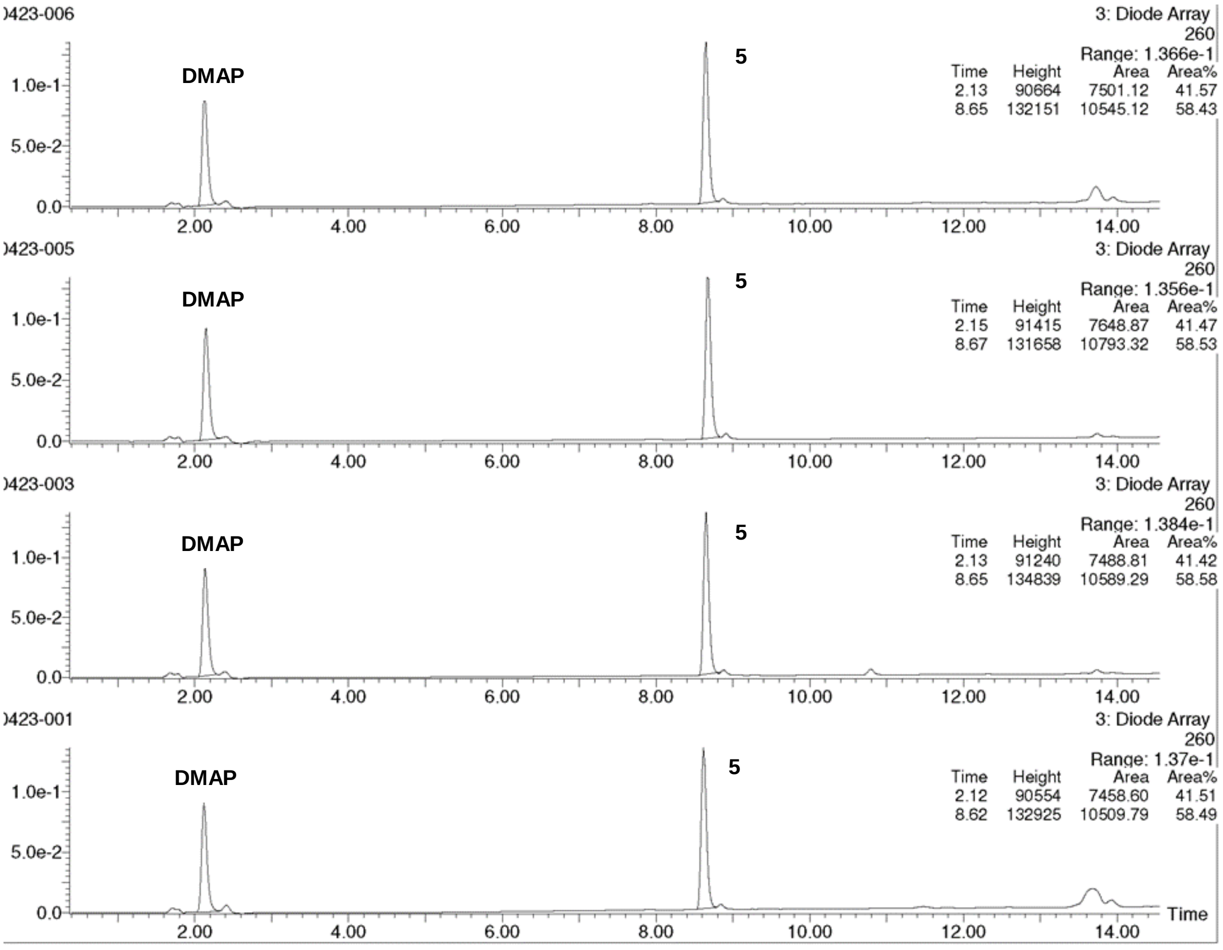

Figure S20. Reversed-phase HPLC-ESI MS traces at 260 nm of COUPY-caged compound **5** after standing for different times in the dark at 37 ºC in a 1:1 (v/v) mixture of PBS buffer and ACN in the presence of DMAP (internal standard). From bottom to top: t= 0, 2 h, 4 h and 5 h.

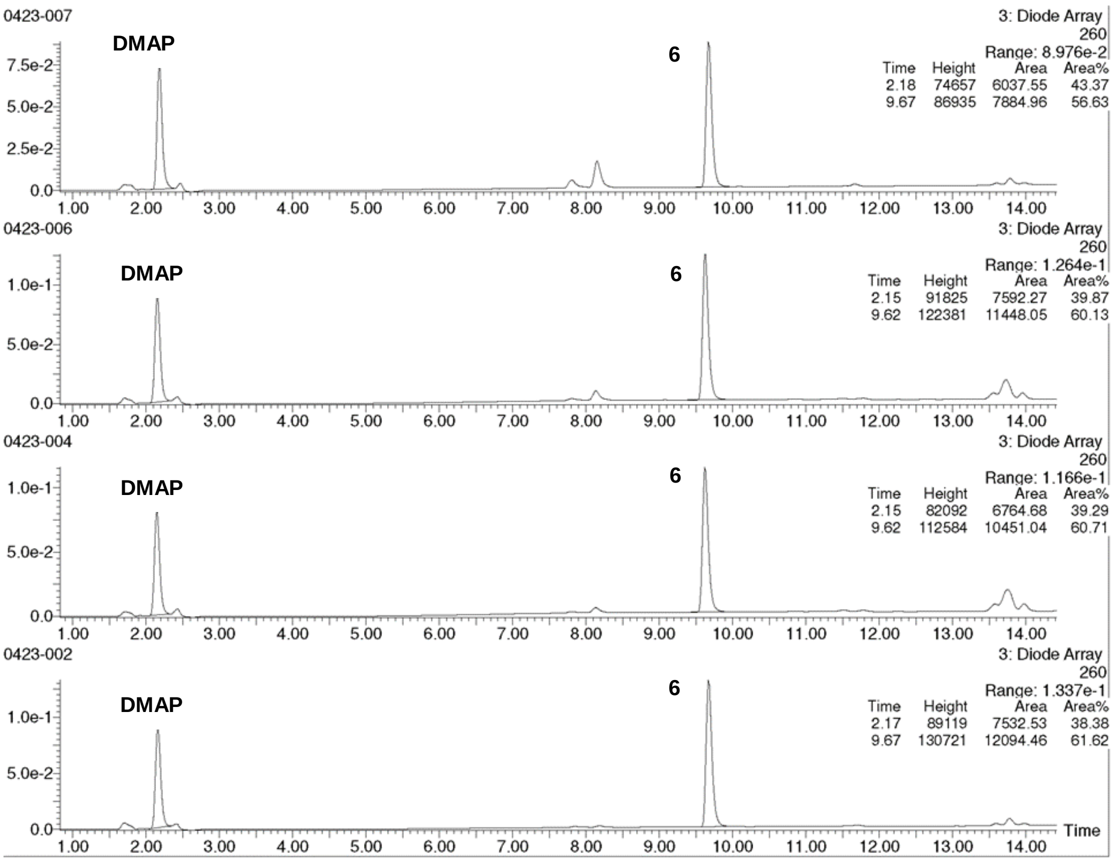

Figure S21. Reversed-phase HPLC-ESI MS traces at 260 nm of COUPY-caged compound **6** after standing for different times in the dark at 37 ºC in a 1:1 (v/v) mixture of PBS buffer and ACN in the presence of DMAP (internal standard). From bottom to top: t= 0, 2 h, 4 h and 5 h.

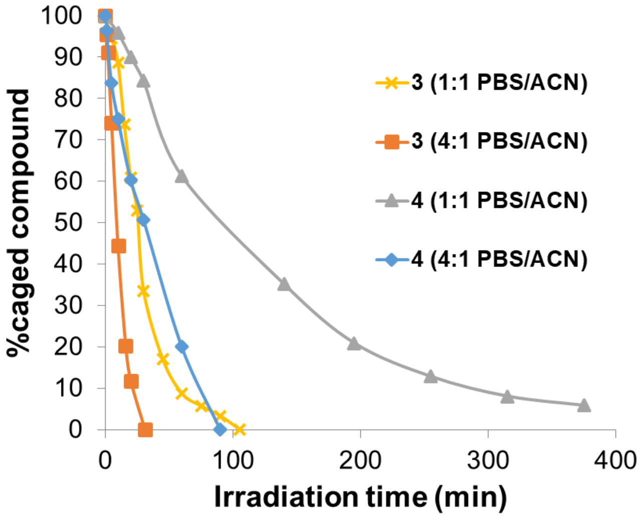

Figure S22. Plot of the temporal evolution of the amounts of COUPY-caged compounds **3** and **4** after irradiation with yellow light (560 ± 40 nm; 40 mW cm^{-2}) in 1:1 or 4:1 (v/v) mixtures of PBS buffer and ACN at 37 ºC. The lines connecting the experimental points are meant to aid the reader in visualizing the data.

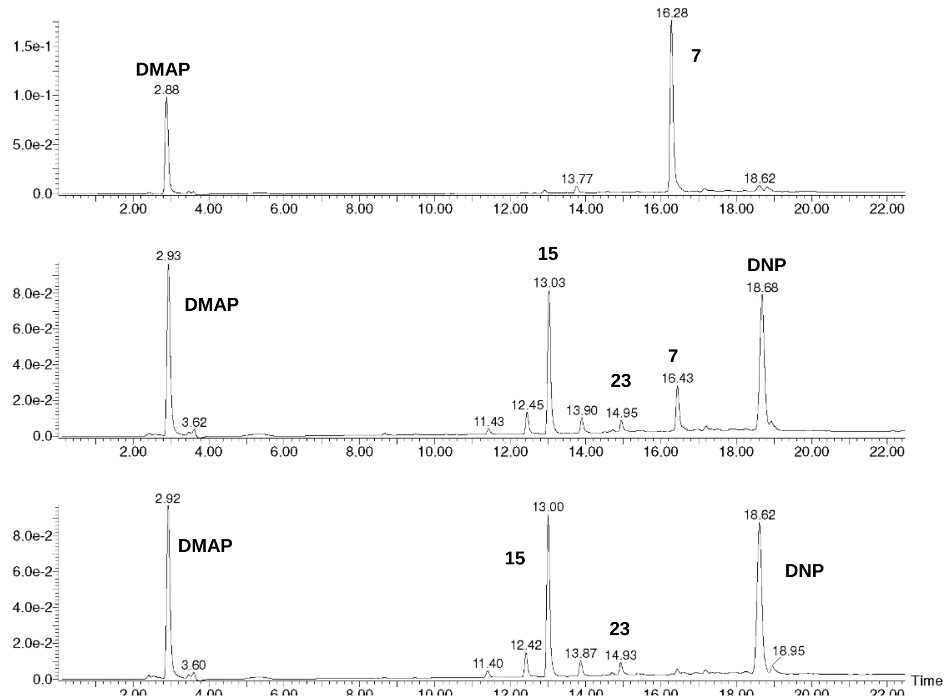

Figure S23. Reversed-phase HPLC-ESI MS traces at 260 nm for the photolysis reaction of **7** in a 1:1 (v/v) mixture of PBS buffer and ACN in the presence of DMAP (internal standard) at t=0 (top) and after irradiation with visible LED light (470-750 nm range, centered at 530 nm) for 9 min (middle) and 25 min (bottom) at 37 ºC.

Table S1. MS data for coumarin photoproducts generated upon photoactivation of compound **7**. Data was obtained from HPLC-ESI MS analysis after 25 min of irradiation (Figure S23).

**Peak (min)**	**% relative**	λ_{max}	***m/z***	**Compound**
11.4	8.1	379	380	-
12.4	10.5	552	417	-
13.0	70.8	552	376	**15**
13.9	6.4	567	374	-
14.9	4.2	581	358	**23**

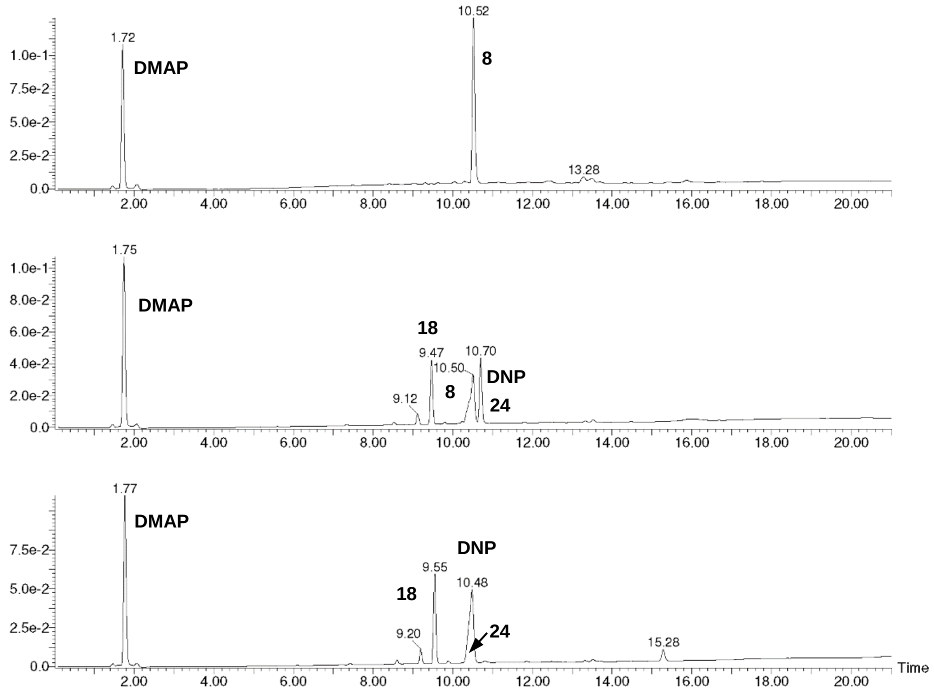

Figure S24. Reversed-phase HPLC-ESI MS traces at 260 nm for the photolysis reaction of **8** in a 1:1 (v/v) mixture of PBS buffer and ACN in the presence of DMAP (internal standard) at t=0 (top) and after irradiation with visible LED light (470-750 nm range, centered at 530 nm) for 2 min (middle) and 8 min (bottom) at 37 ºC.

Table S2. MS data for coumarin photoproducts generated upon photoactivation of compound **8**. Data was obtained from HPLC-ESI MS analysis after 8 min of irradiation (Figure S24). Since DNP and compound **24** co-elute, integration of coumarin photoproducts was carried out at 400 nm where DNP does not give signal.

Peak (min)	% relative	λ_{max}	*m/z*	Compound
8.7	5.0	369	450	-
9.2	11.7	555	487	-
9.5	63.6	553	446	**18**
10.0	1.9	567	444	-
10.5	11.9	582	428	**24**
10.7	5.9	565	612	**8**

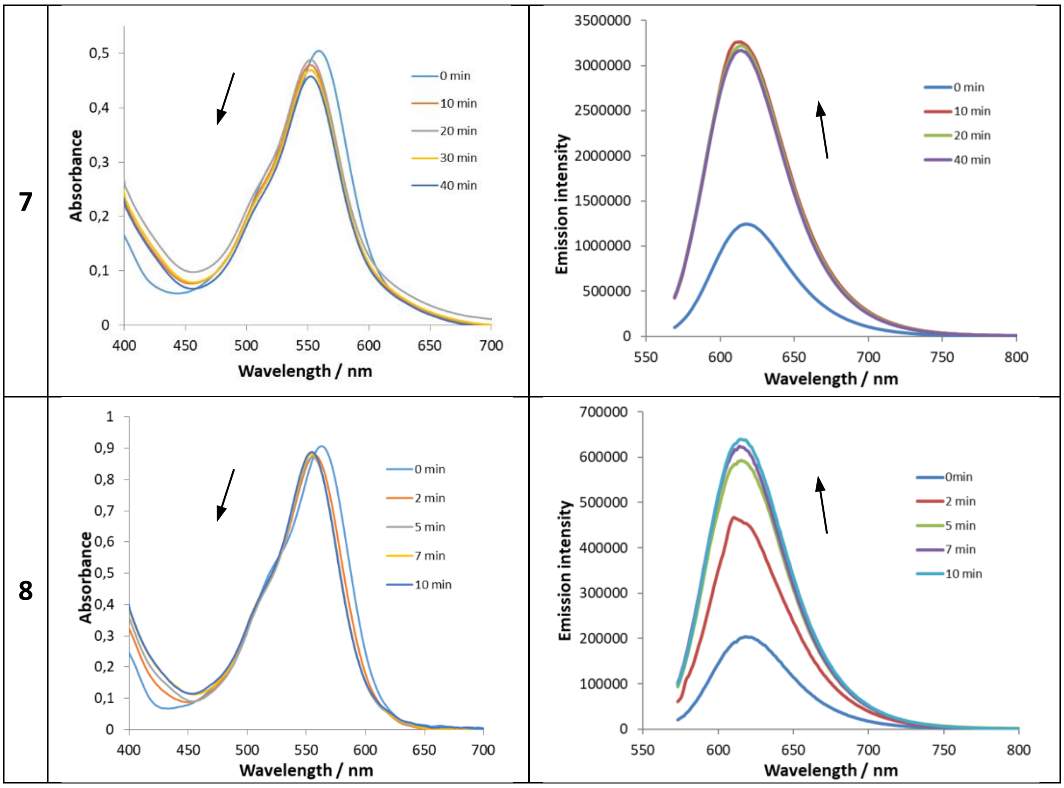

Figure S25. Temporal evolution of the solution absorbance and emission intensity of COUPY caged compounds **7-8** upon illumination with a LED source covering from the cyan to the far-red region of the visible spectrum (470–750 nm; 150 mW cm^{-2}). All the experiments were performed in a 1:1 (v/v) mixture of PBS buffer and ACN at 37 ºC.

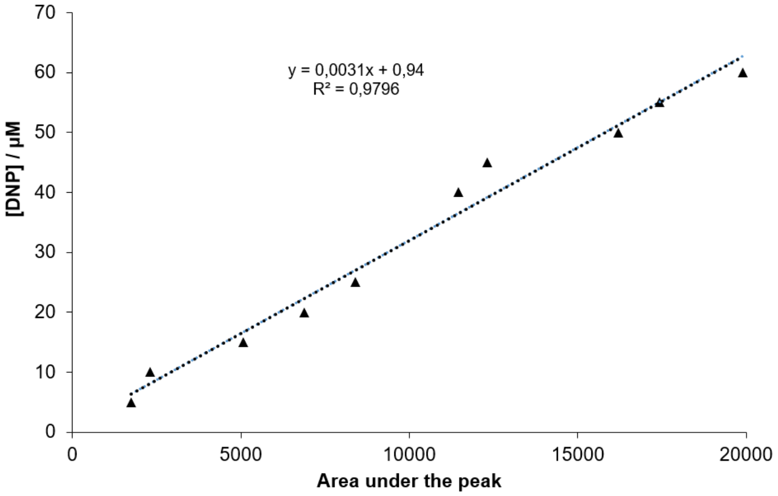

Figure S26. DNP calibration curve prepared for 5–60 µM concentration.

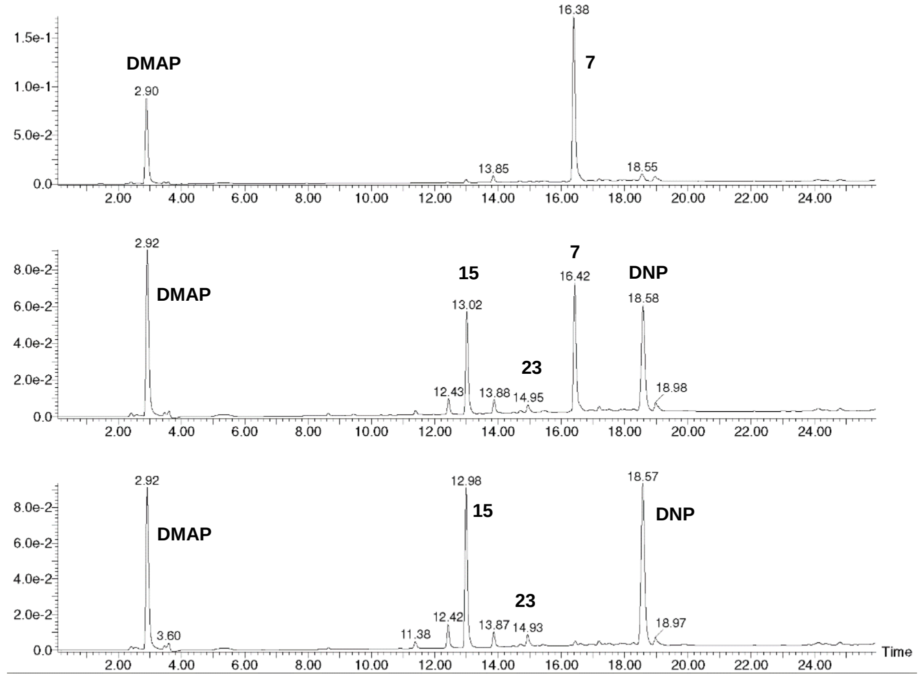

Figure S27. Reversed-phase HPLC-ESI MS traces at 260 nm for the photolysis reaction of **7** in a 1:1 (v/v) mixture of PBS buffer and ACN in the presence of DMAP (internal standard) at t=0 (top) and after irradiation with red LED light (620 nm) for 22 min (middle) and 154 min (bottom) at 37 ºC.

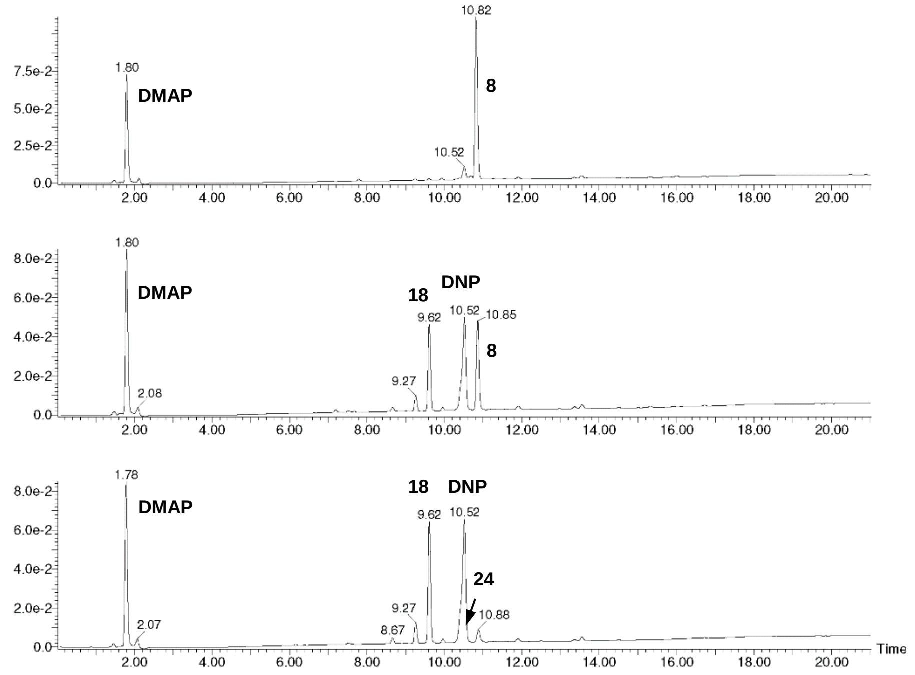

Figure S28 Reversed-phase HPLC-ESI MS traces at 260 nm for the photolysis reaction of **8** in a 1:1 (v/v) mixture of PBS buffer and ACN in the presence of DMAP (internal standard) at t=0 (top) and after irradiation with red LED light (620 nm) for 15 min (middle) and 56 min (bottom) at 37 ºC

5.- Confocal microscopy studies

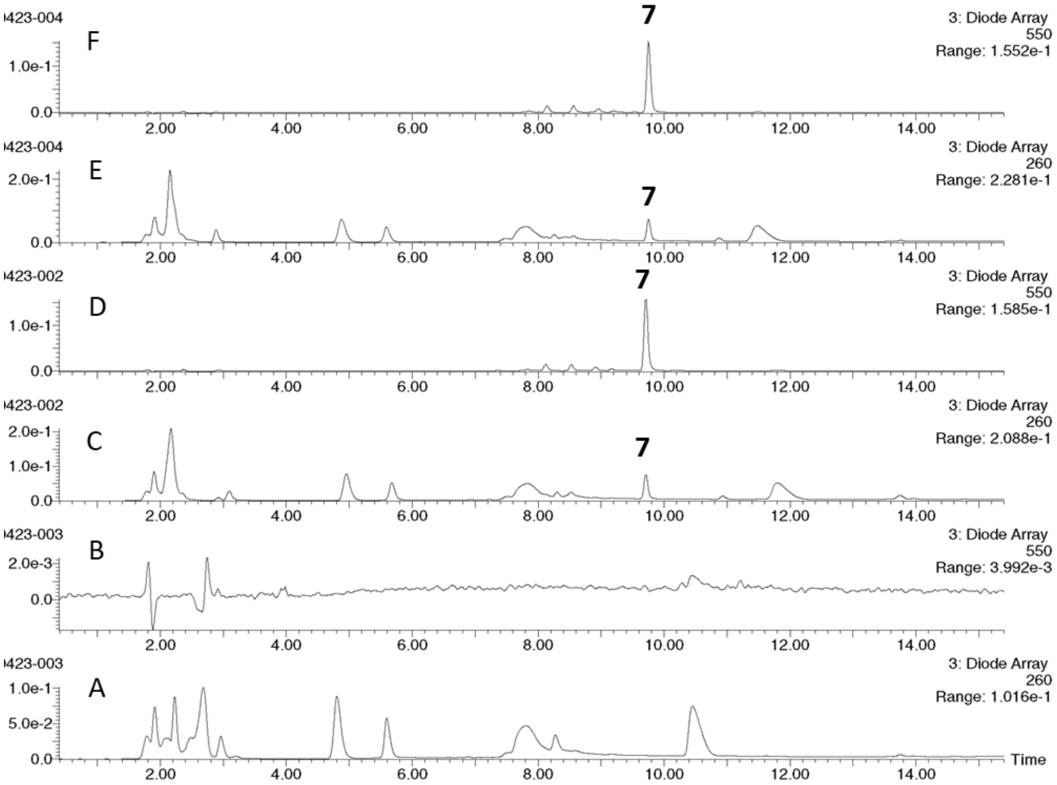

Figure S29. Dark stability of COUPY photocage **7** in Dullbecco Modified Eagle Medium (DMEM) containing high glucose and supplemented with 10% fetal bovine serum (FBS) and 50 U/mL penicillin-streptomycin. Reversed-phase HPLC-ESI MS traces of cell culture medium at 260 nm (A) and at 550 nm (B), and of a solution of compound **7** in cell culture medium at t = 0 (C: 260 nm and D: 550 nm) and after standing for 1 h in the dark at 37 ºC (E: 260 nm and F: 550 nm).

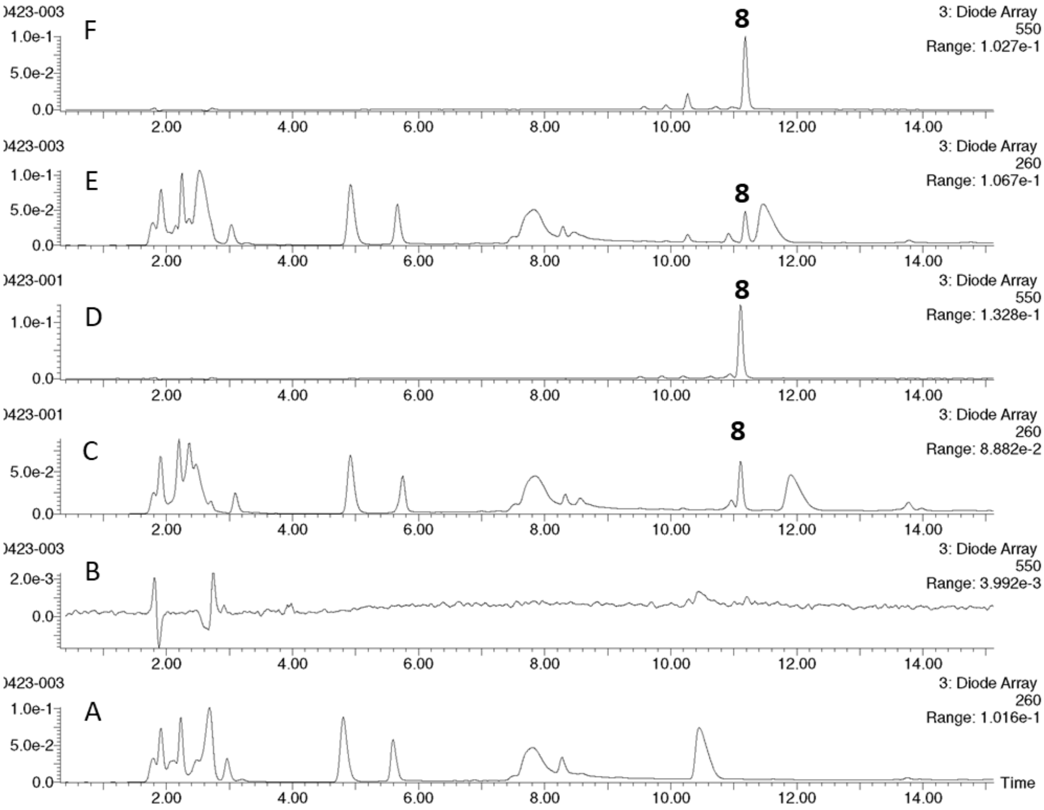

Figure S30 Dark stability of COUPY photocage **8** in Dullbecco Modified Eagle Medium (DMEM) containing high glucose and supplemented with 10% fetal bovine serum (FBS) and 50 U/mL penicillin-streptomycin. Reversed-phase HPLC-ESI MS traces of cell culture medium at 260 nm (A) and at 550 nm (B), and of a solution of compound **8** in cell culture medium at t = 0 (C: 260 nm and D: 550 nm) and after standing for 1 h in the dark at 37 ^{o}C (E: 260 nm and F: 550 nm).

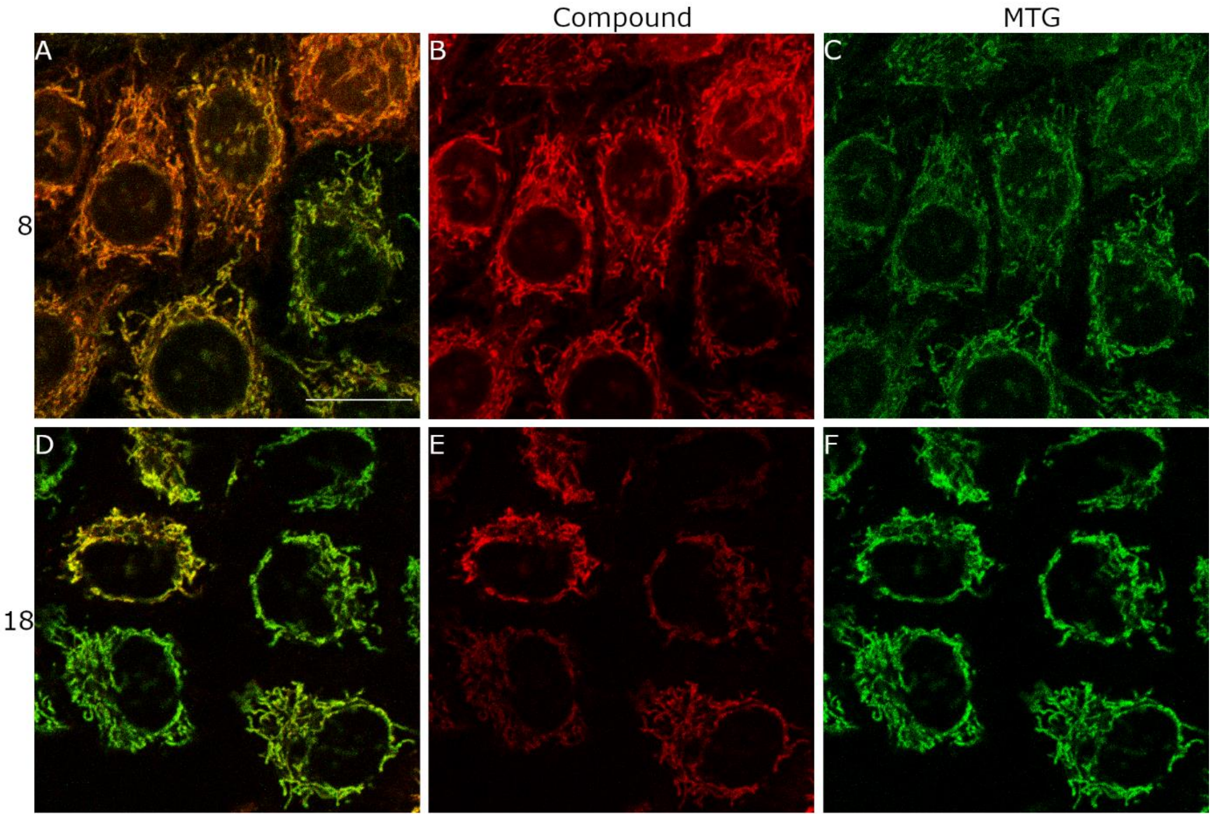

Figure S31. Co-localization studies with COUPY photocage **8** (top) and coumarin alcohol **18** (bottom) with Mitotracker Green FM. Single confocal planes of HeLa cells incubated with **8** or **18** (2 μM, red) and MTG (0.1 μM, green). A), D) Overlay of the two staining. B), E) compounds **8** and **18** signal, respectively. C), F) MTG signal. Scale bar: 20 μm. All images are at the same scale as A.

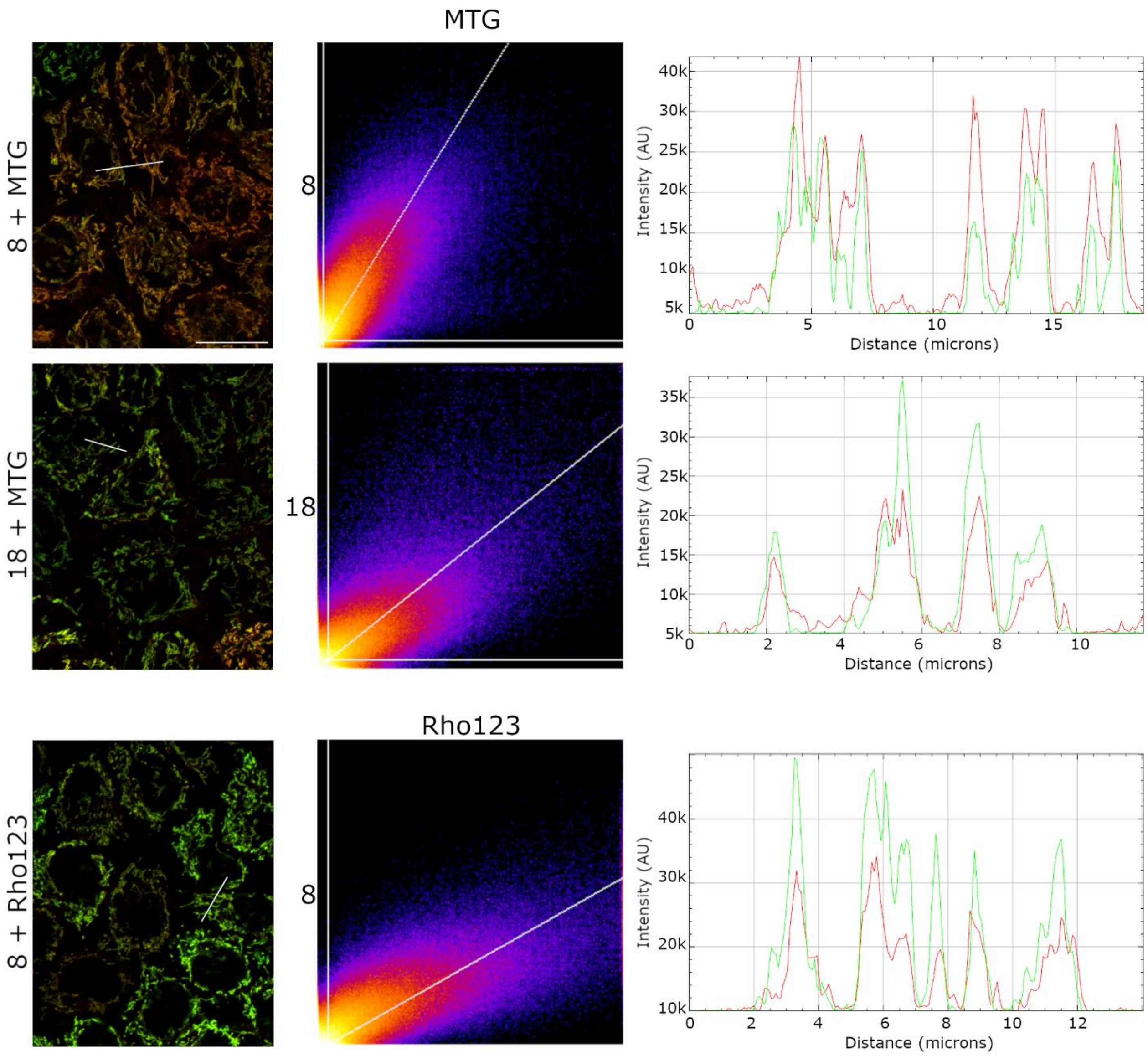

Figure S32. Visual inspection of co-localization experiments. Left column shows single confocal planes of HeLa cells with merged stainings of COUPY photocage **8** (mM 2, red) and MTG (0.1 mM, green, top row), coumarin alcohol **18** (2 mM, red) with MTG (0.1 mM, green, central row) and COUPY photocage **8** (2 mM, red) with Rho123 (26 mM, green, bottom row). Scale bar: 20 mm. Central column shows the scatterplots of fluorescence signals of left column images. Right column shows the plot profiles of fluorescence intensities along the lines drawn on left column images.

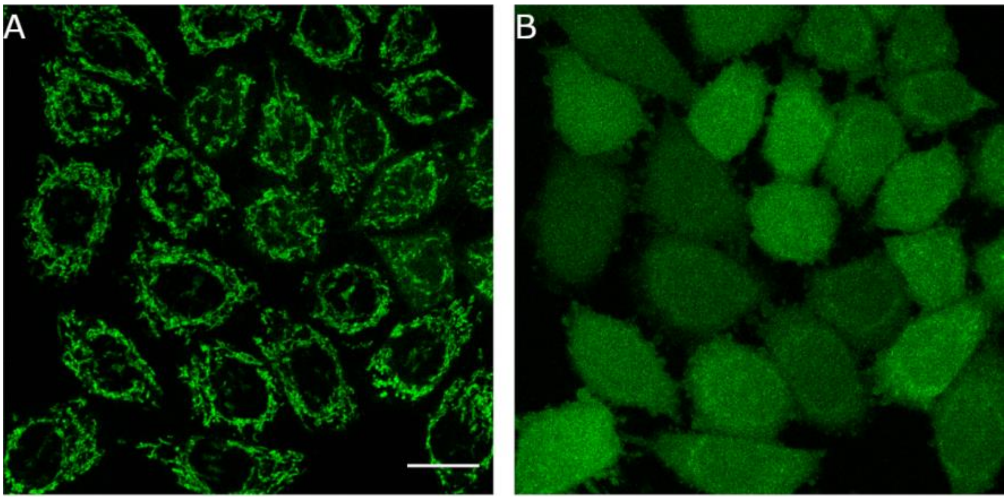

Figure S33. Single confocal planes of HeLa cells incubated with Rho123 (26 μM, 15 min) before (A) and after addition (B) of DNP (200 μM). Scale bar: 20 μm.

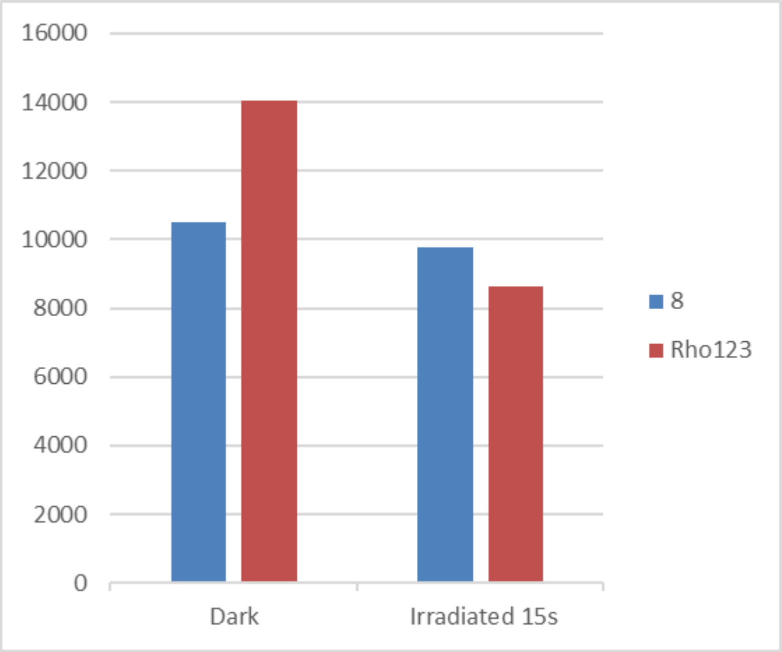

Figure S34. Mean intensity (Arbitrary Units) graph of COUPY photocage **8** and Rho123 at the mitochondria before and after irradiation with yellow light (BP 545/25 filter, 1.4 mW/cm^2, 15 s).

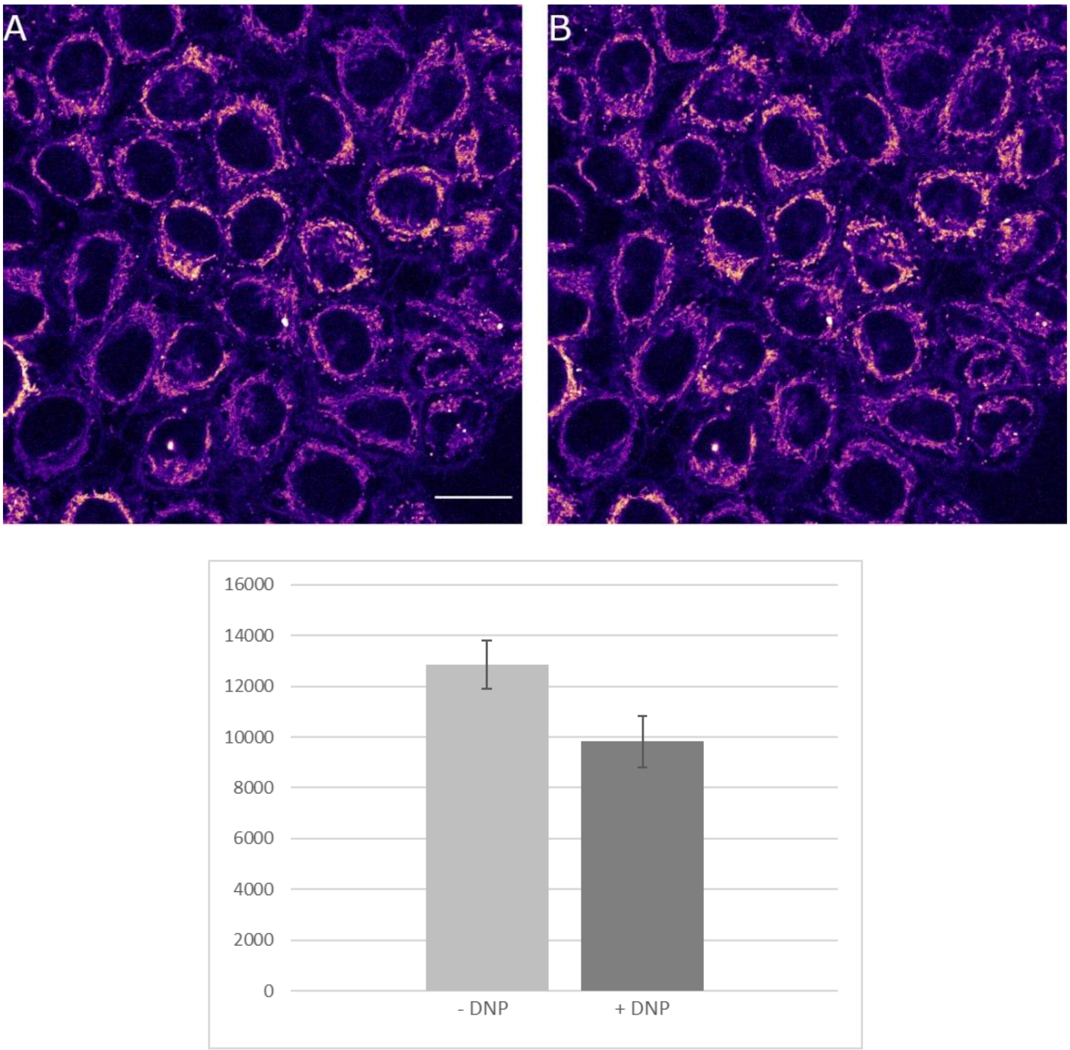

Figure S35. Top: Single confocal planes of HeLa cells incubated with coumarin **18** (2 μM, 30 min) before (A) and after addition (B) of DNP (2 μM). Scale bar: 20 μm. Bottom: Mean intensity (Arbitrary Units) graph of coumarin **18** before and after addition of DNP (2.0 μM, 37 °C, X min).

5.- ^{1}H, ^{13}C and ^{19}F NMR spectra and HR ESI-MS of the compounds

Compound 12

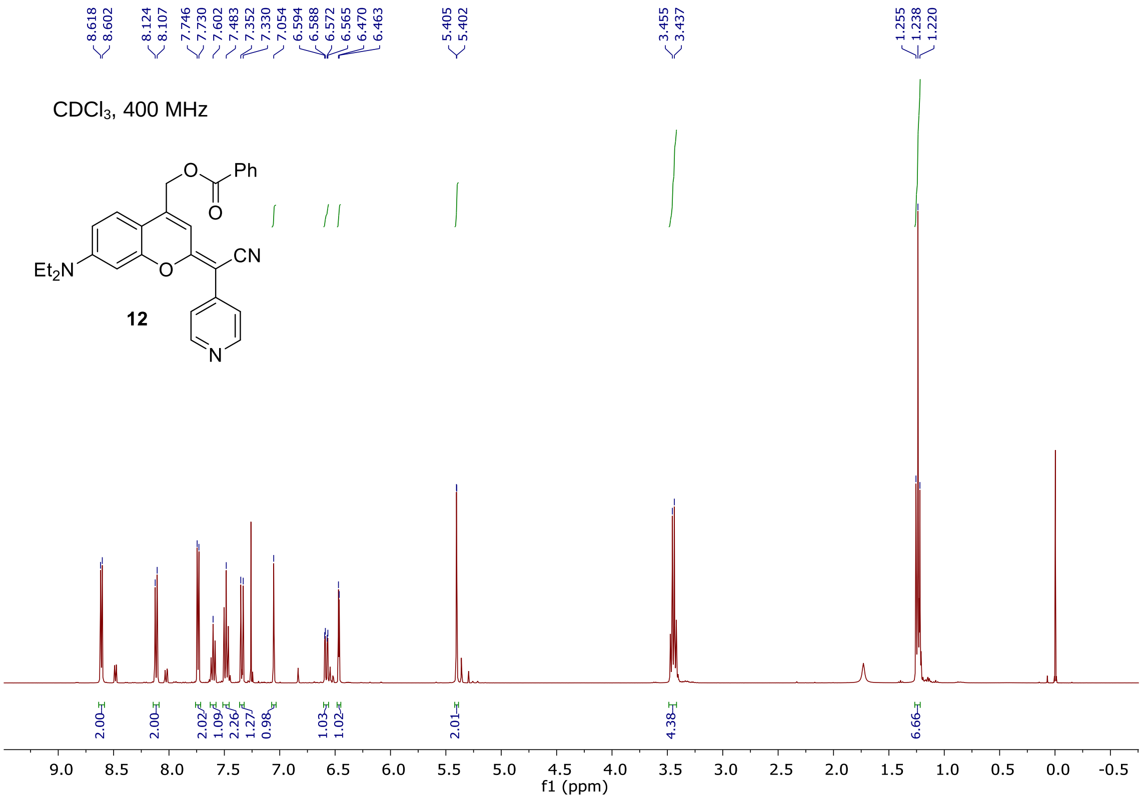

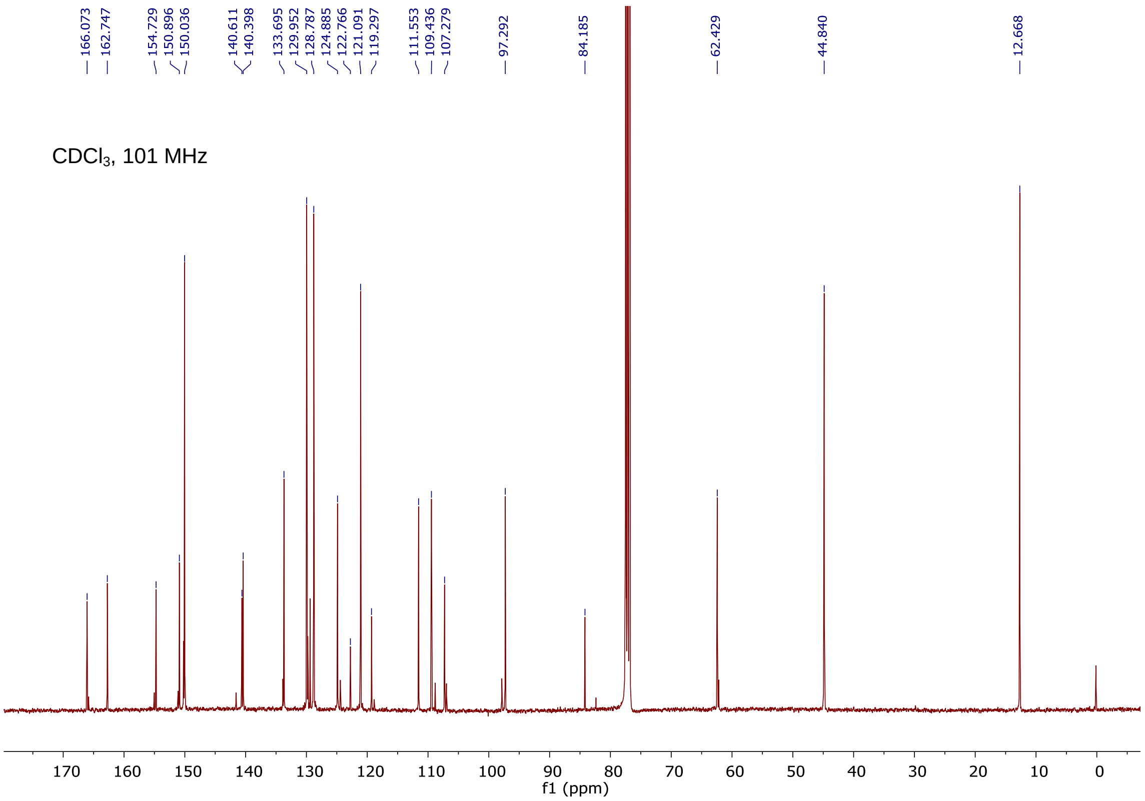

Figure S36. ^{1}H and ^{13}C NMR spectra of compound **12** in $CDCl_3$.

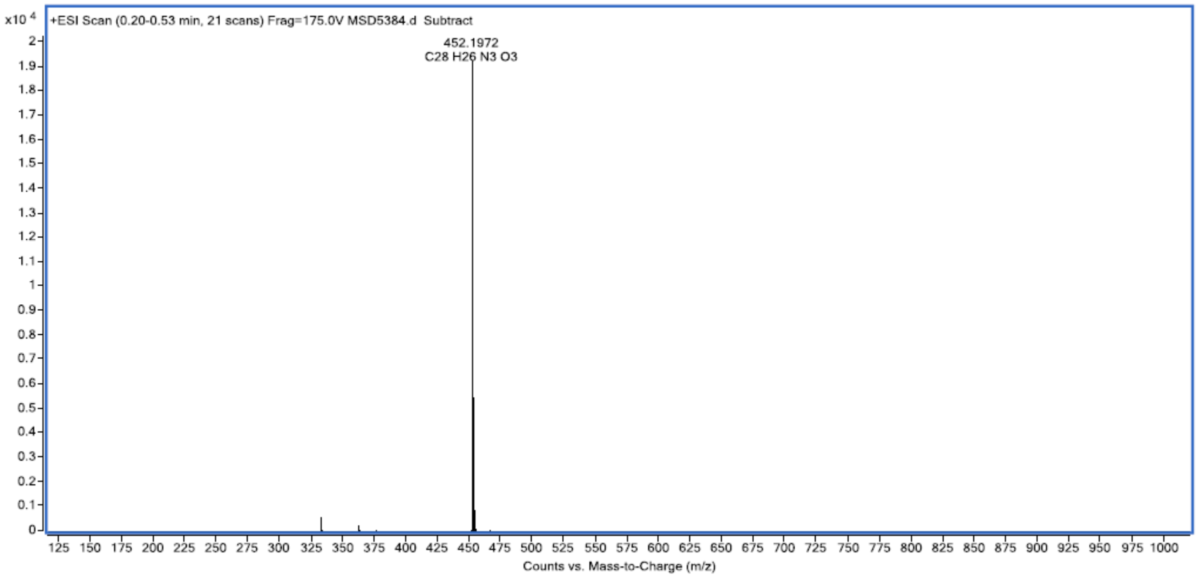

Figure S37. HR ESI-MS spectrum of compound **12**.

Compound 13

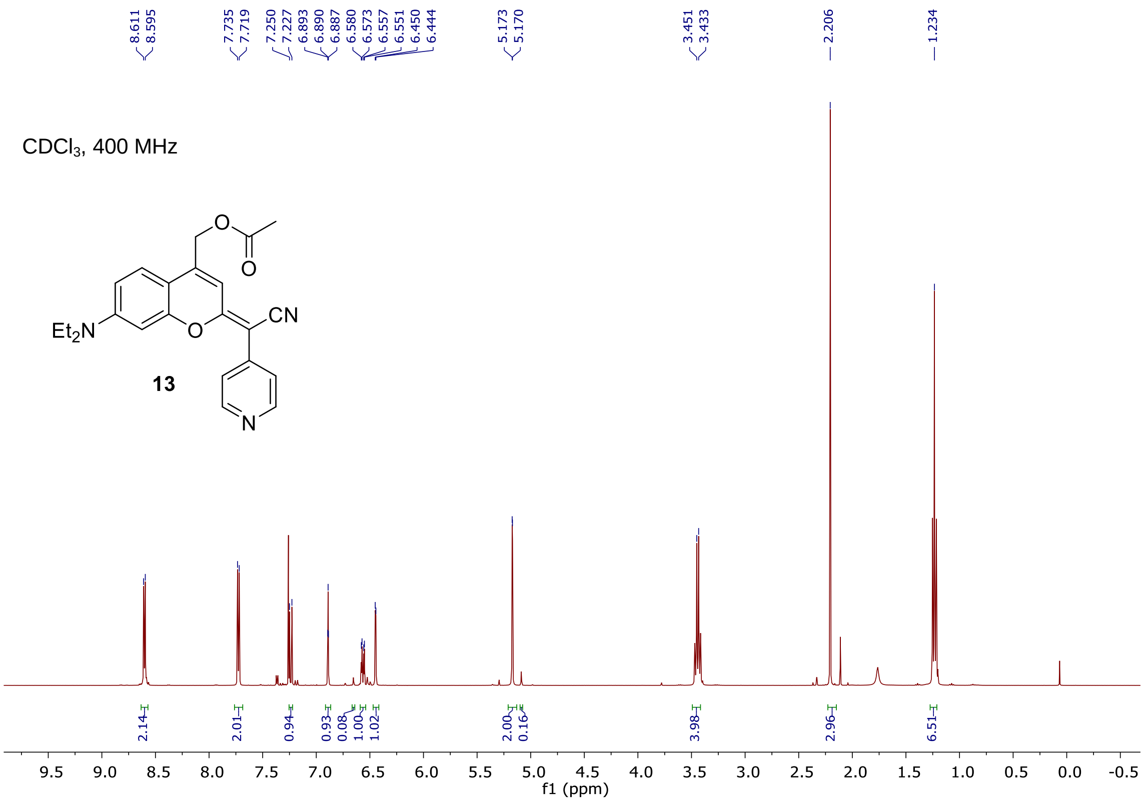

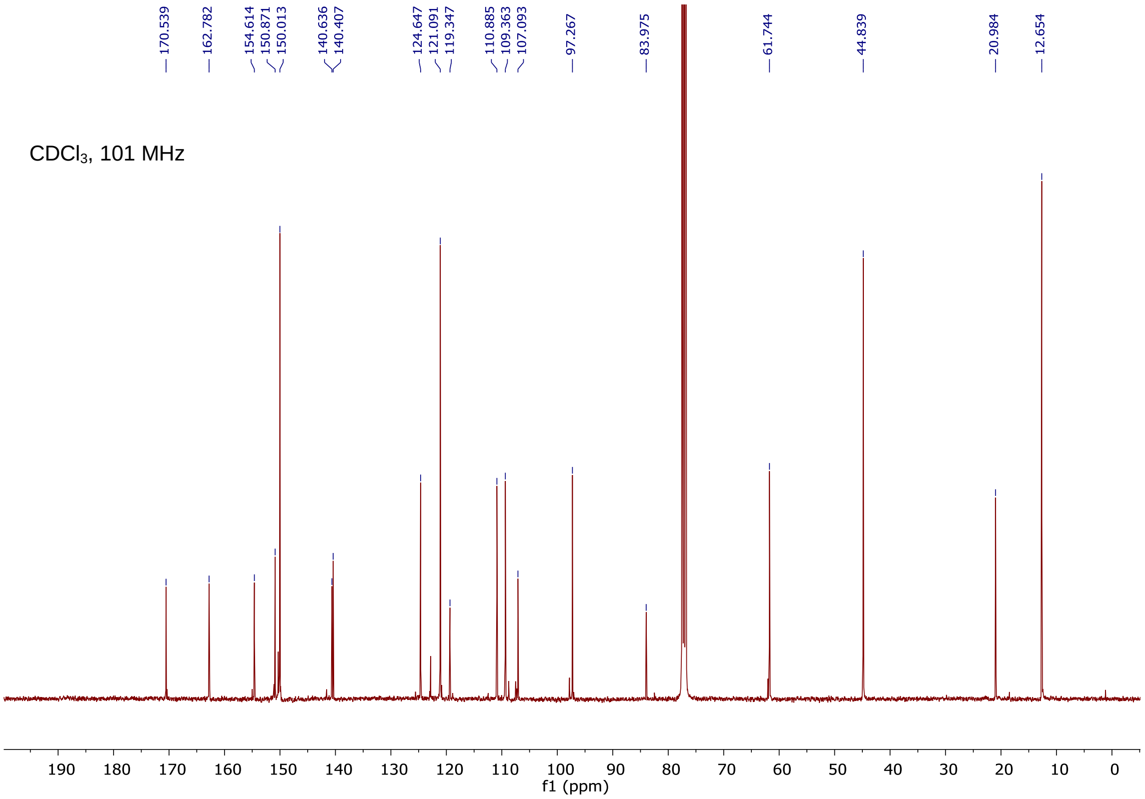

Figure S38. ^{1}H and ^{13}C NMR spectra of compound **13** in $CDCl_3$.

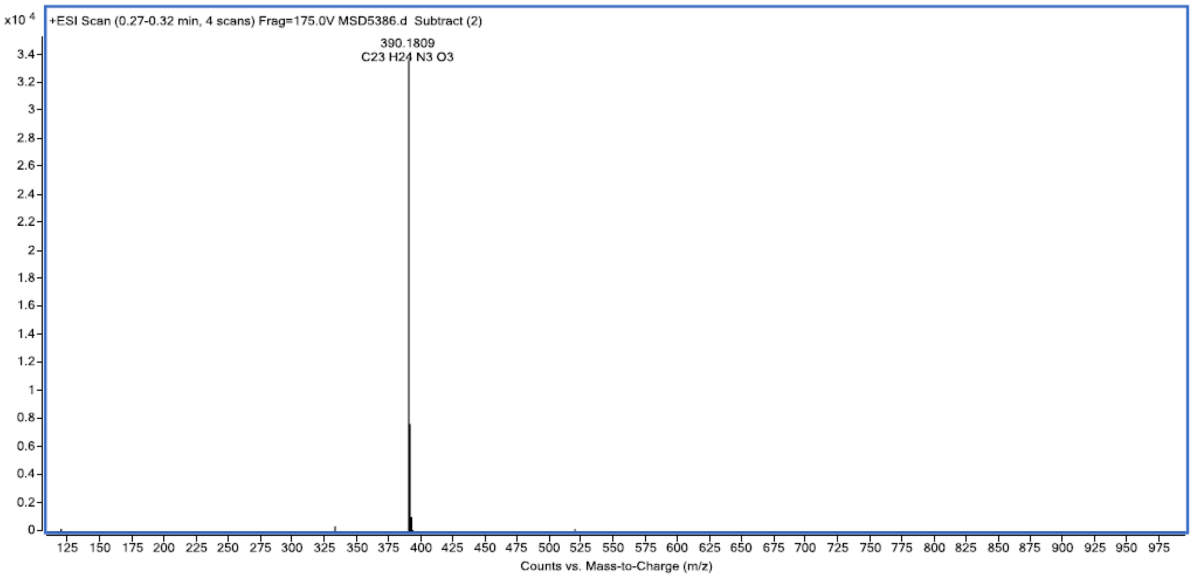

Figure S39. HR ESI-MS spectrum of compound **13**.

Compound 14

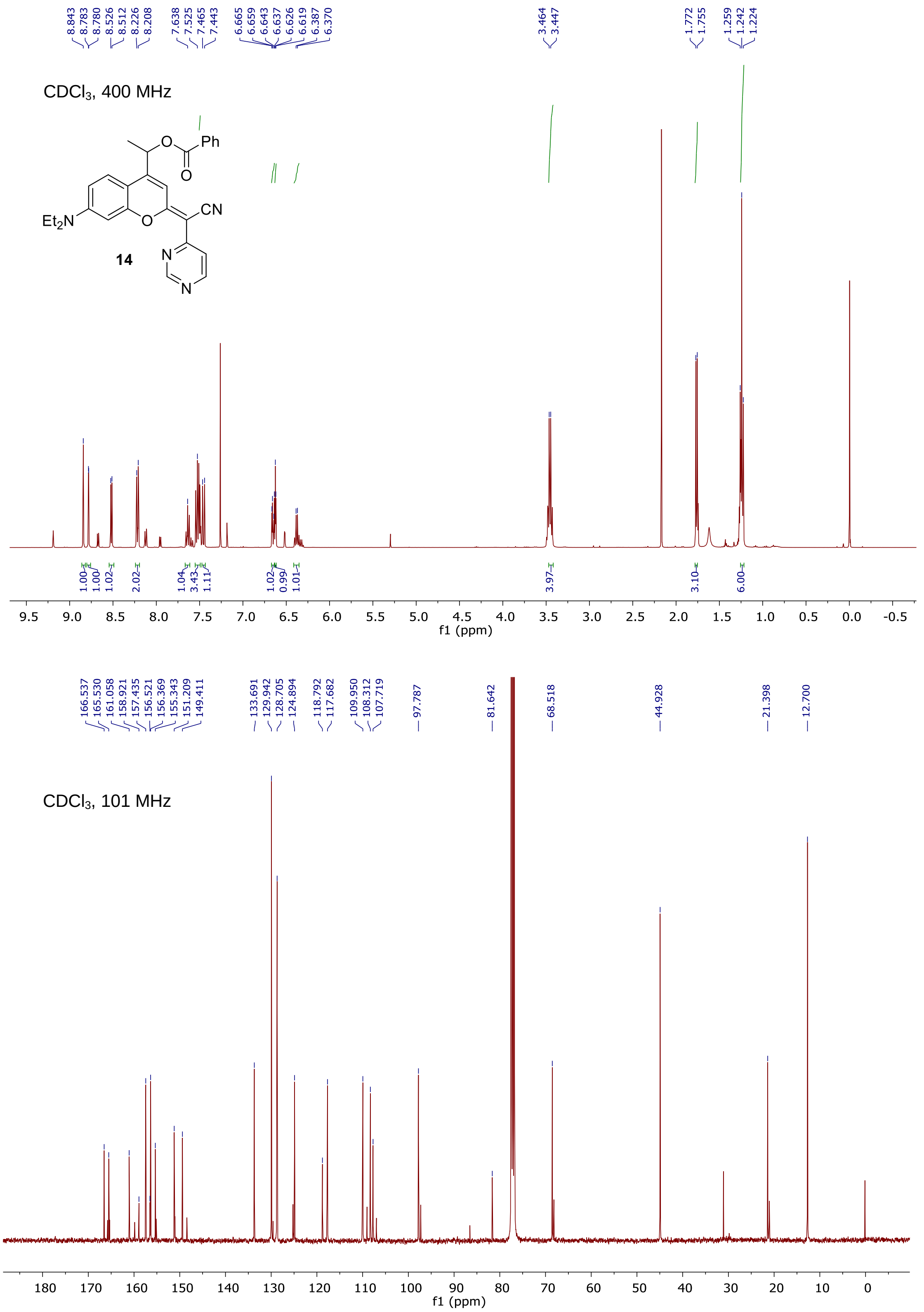

Figure S40. ^{1}H and ^{13}C NMR spectra of compound **14** in $CDCl_3$.

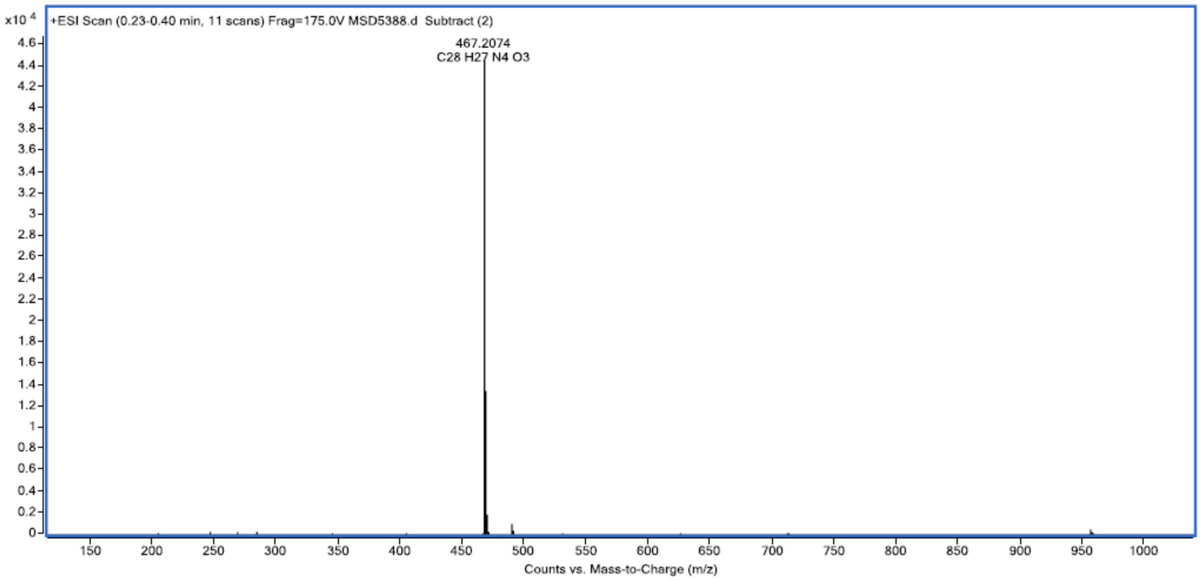

Figure S41. HR ESI-MS spectrum of compound **14**.

Compound 15

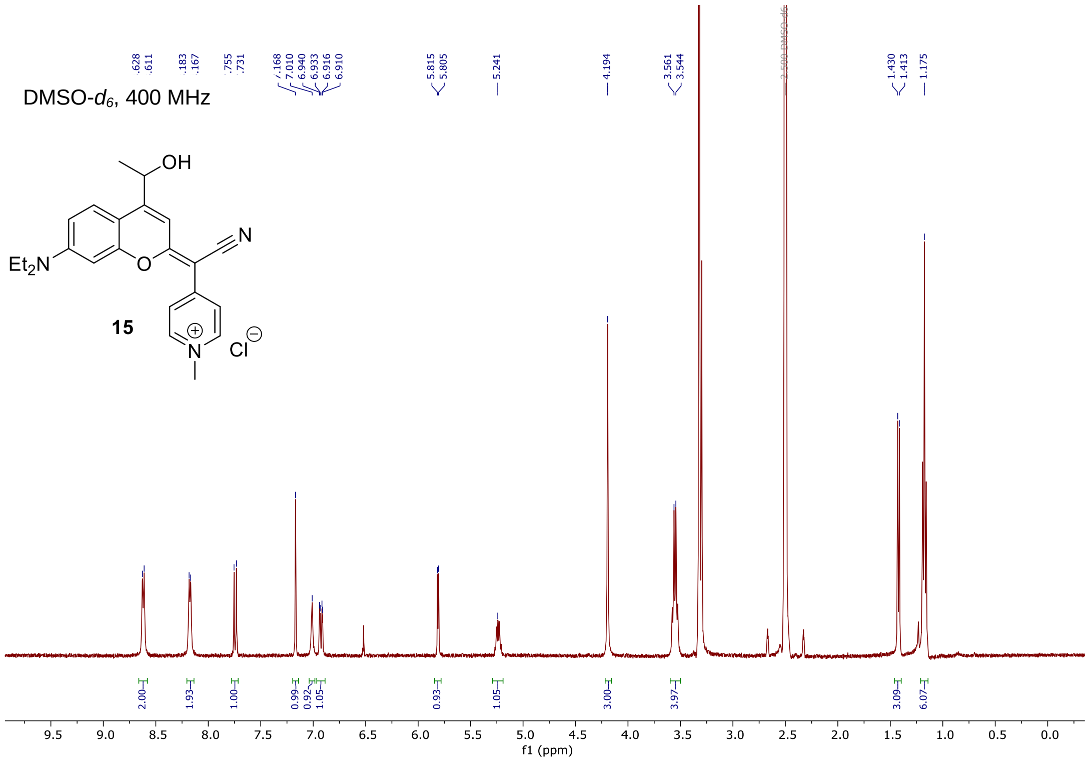

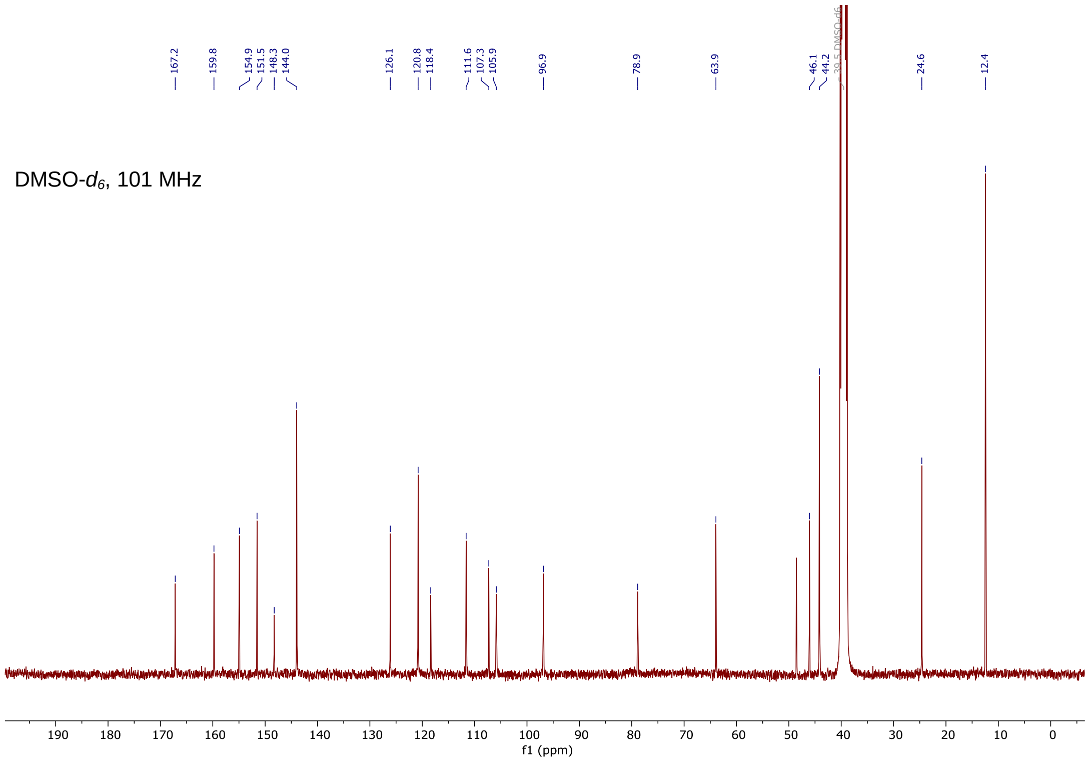

Figure S42. ^{1}H and ^{13}C NMR spectra of compound **15** in DMSO-d_6.

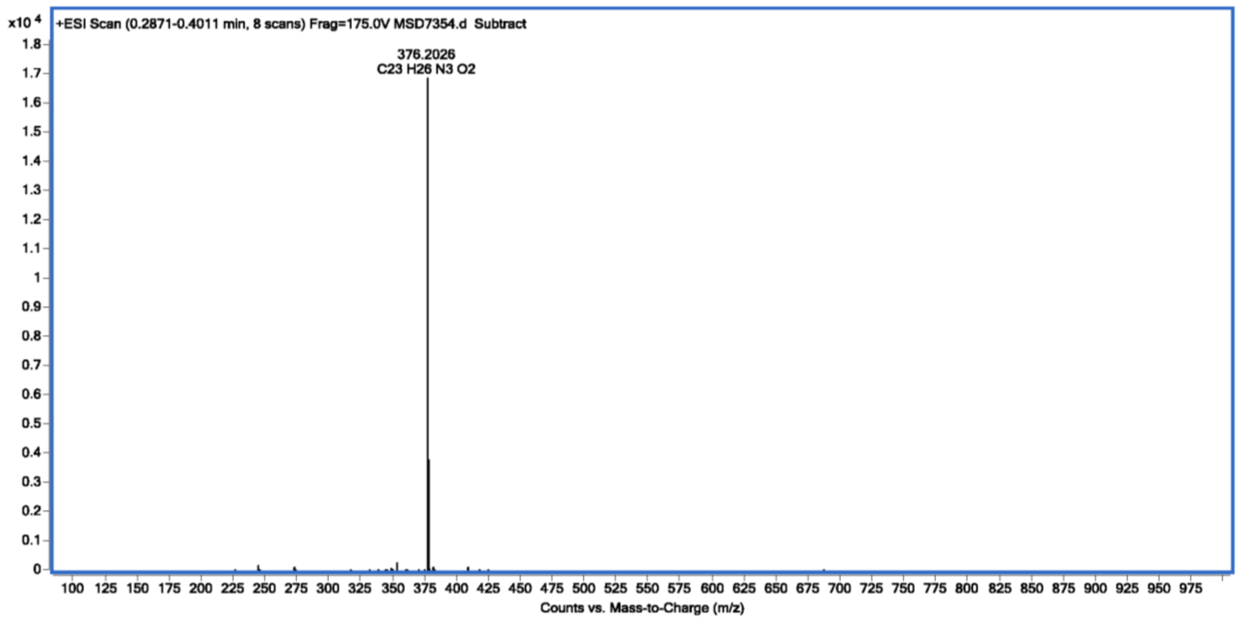

Figure S43. HR ESI-MS spectrum of compound **15**.

Compound 16

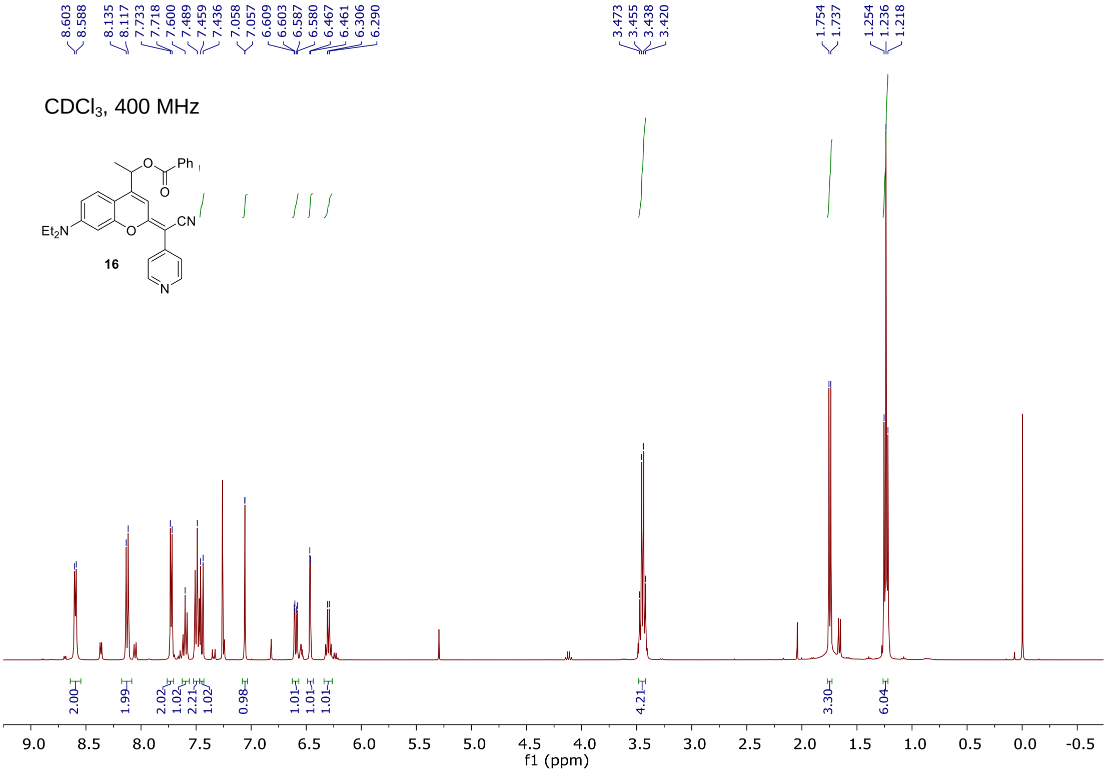

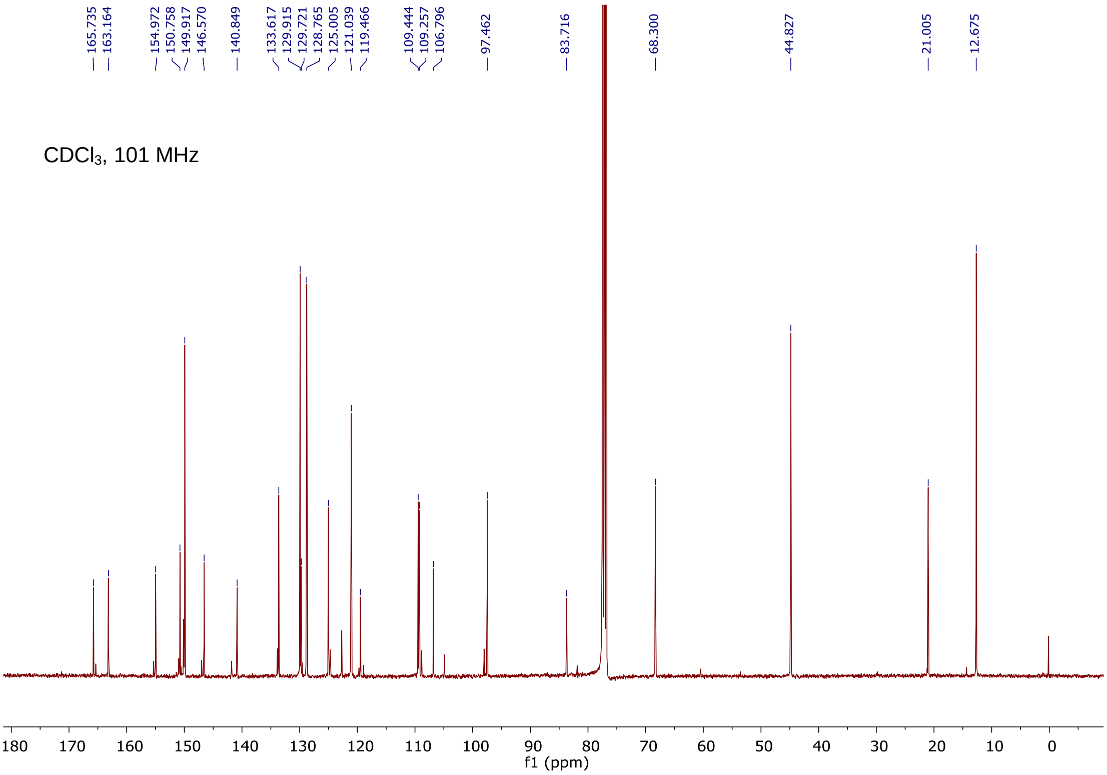

Figure S44. ^{1}H and ^{13}C NMR spectra of compound **16** in $CDCl_3$.

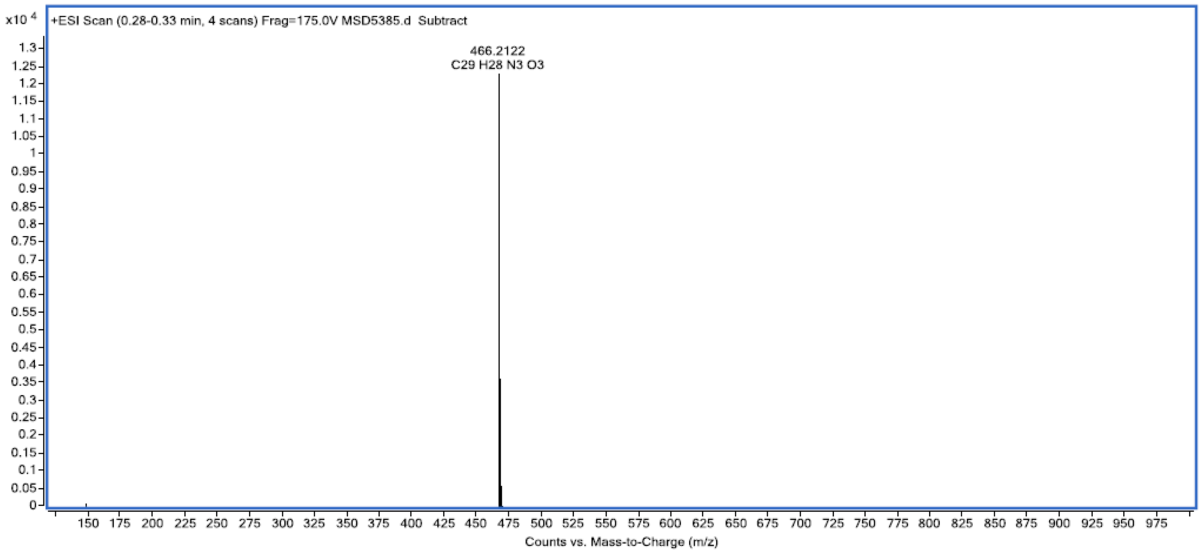

Figure S45. HR ESI-MS spectrum of compound **16**.

Compound 17

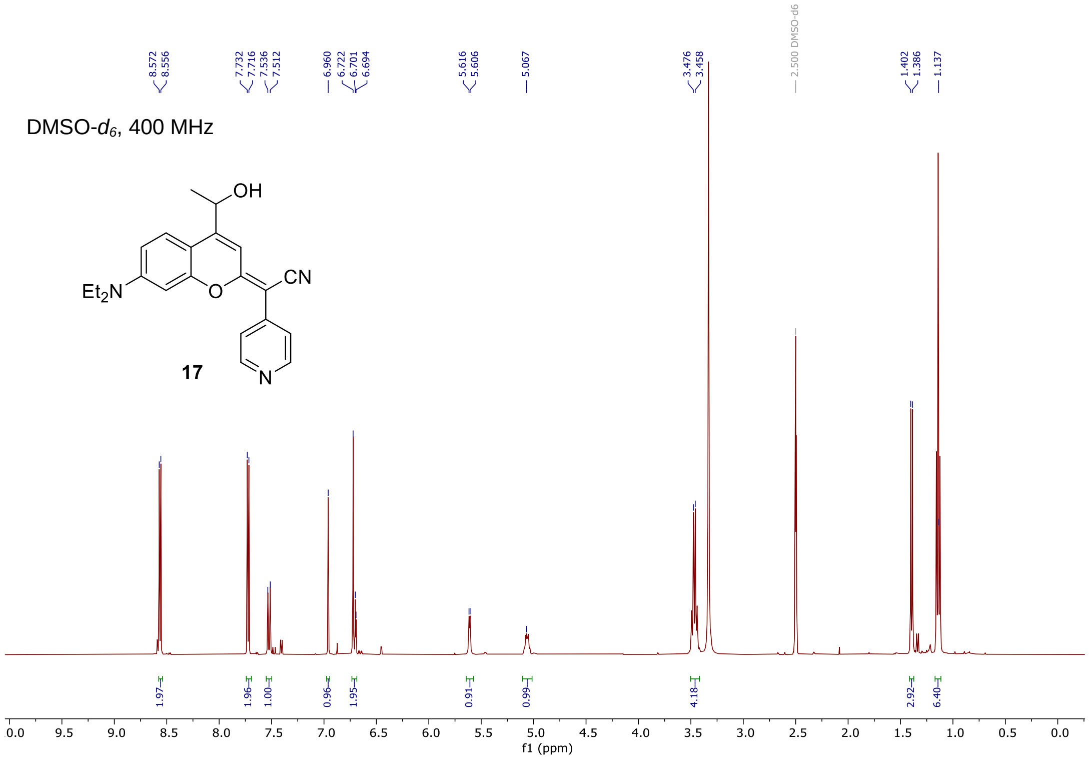

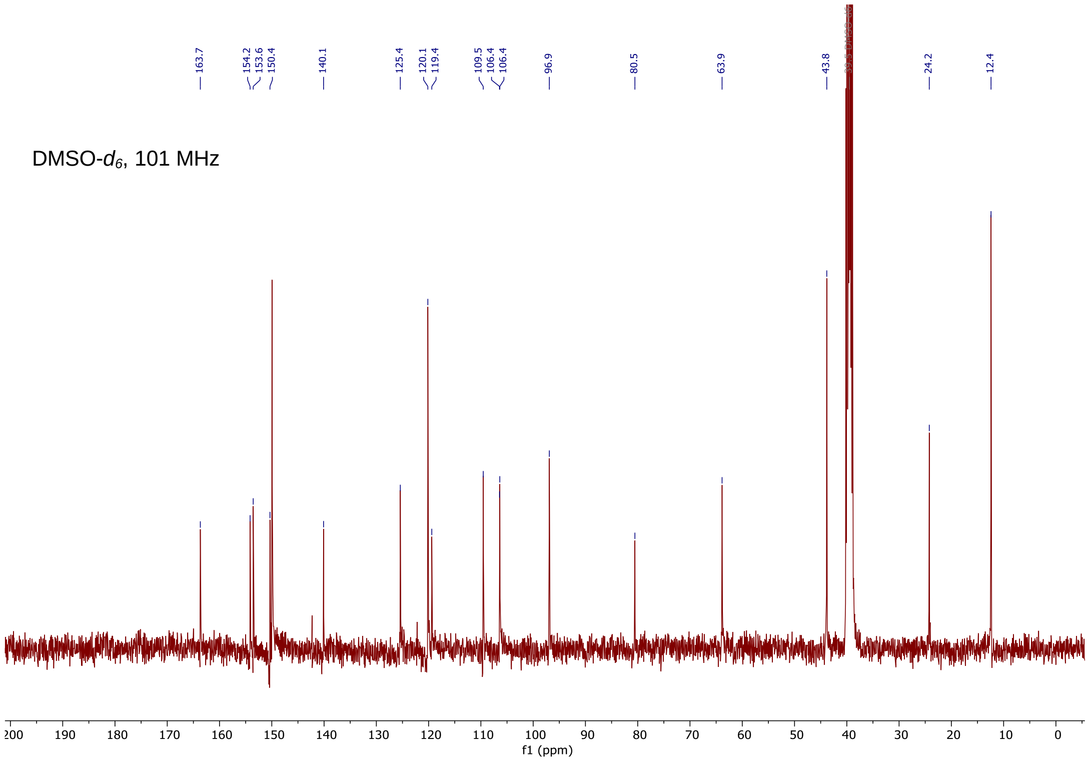

Figure S46. ^{1}H and^{13}C NMR spectra of compound **36** in DMSO-d_6.

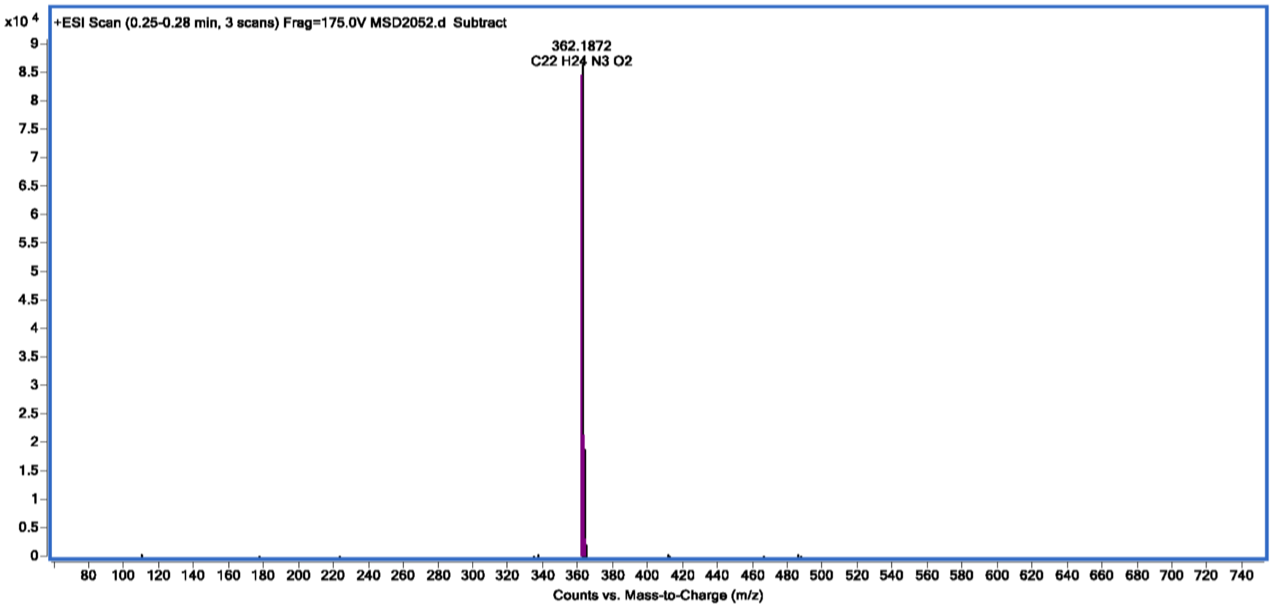

Figure S47. HR ESI-MS spectrum of compound **17**.

Compound 18

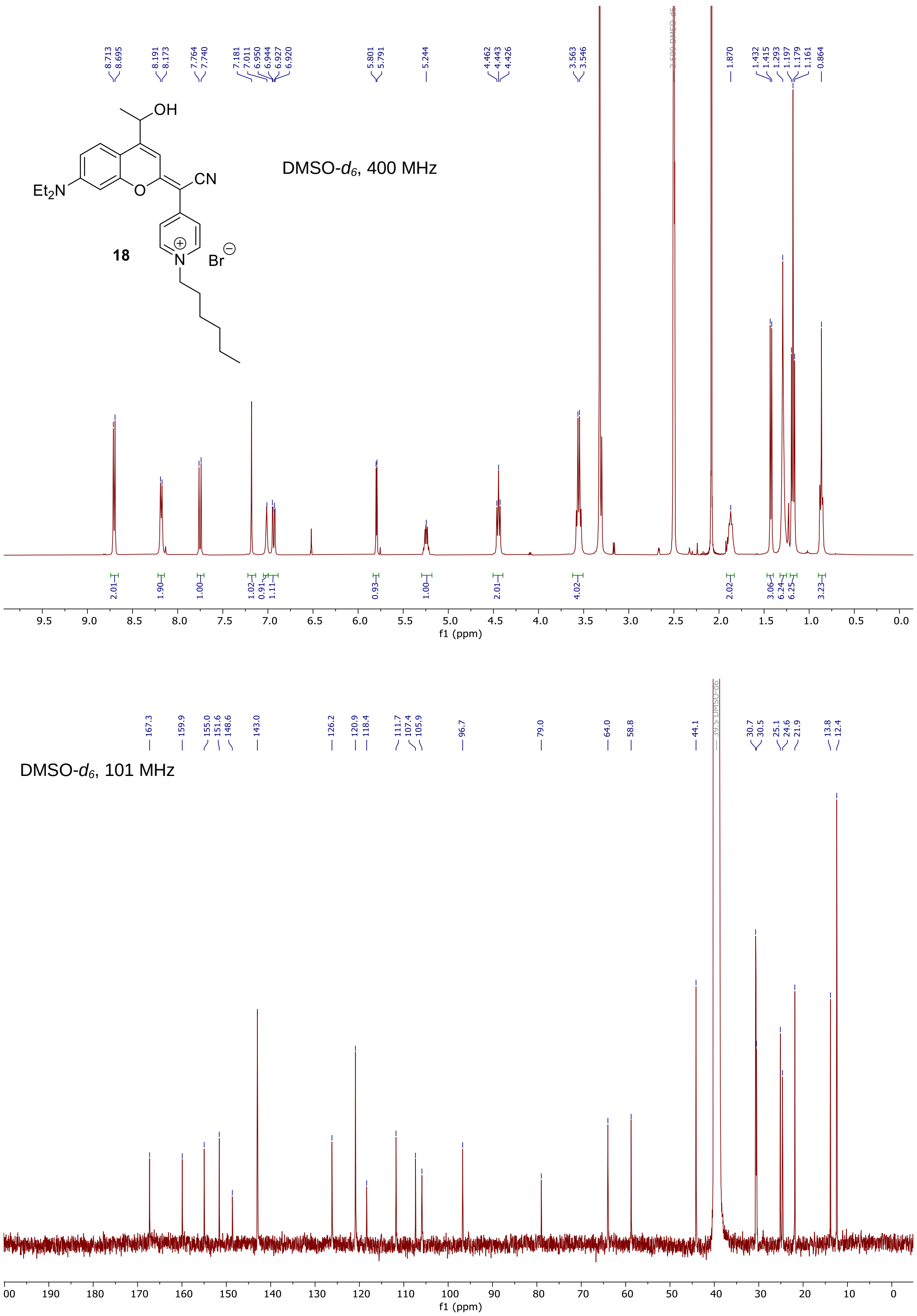

Figure S48. ^{1}H and ^{13}C NMR spectra of compound **18** in DMSO-d_6.

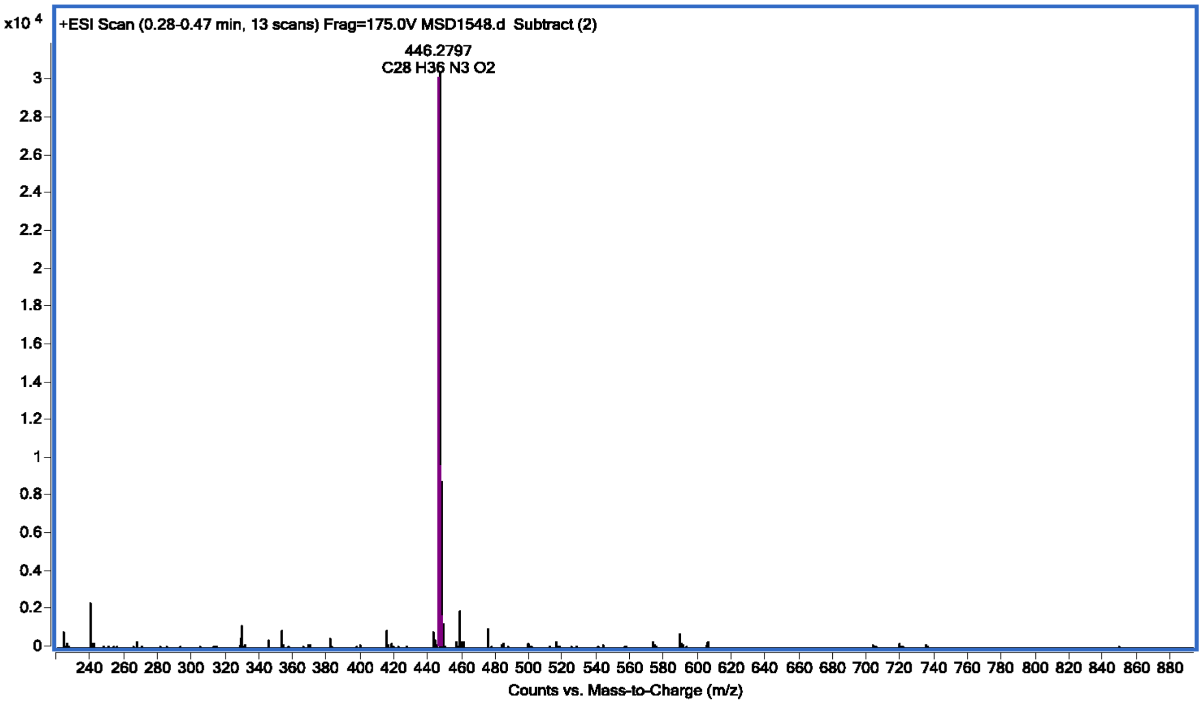

Figure S49. HR ESI-MS spectrum of compound **18**.

Compound 3

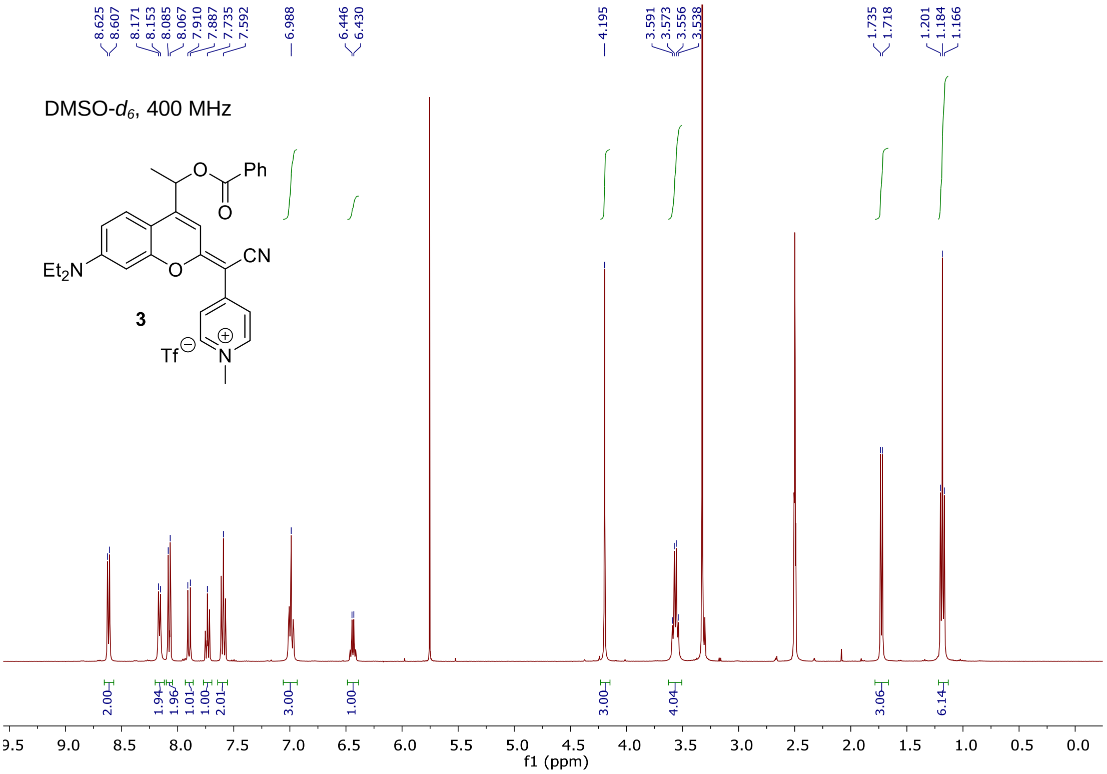

8.625
8.607
8.171
8.153
8.085
8.067
7.910
7.887
7.735
7.592
6.988
6.446
6.430
4.195
3.591
3.573
3.556
3.538
1.735
1.718
1.201
1.184
1.166
DMSO-d_6, 400 MHz
Ph
O
O
CN
Et_2N
O
3
Tf
N
2.00
1.94
1.96
1.01
1.00
2.01
3.00
1.00
3.00
4.04
3.06
6.14
f1 (ppm)

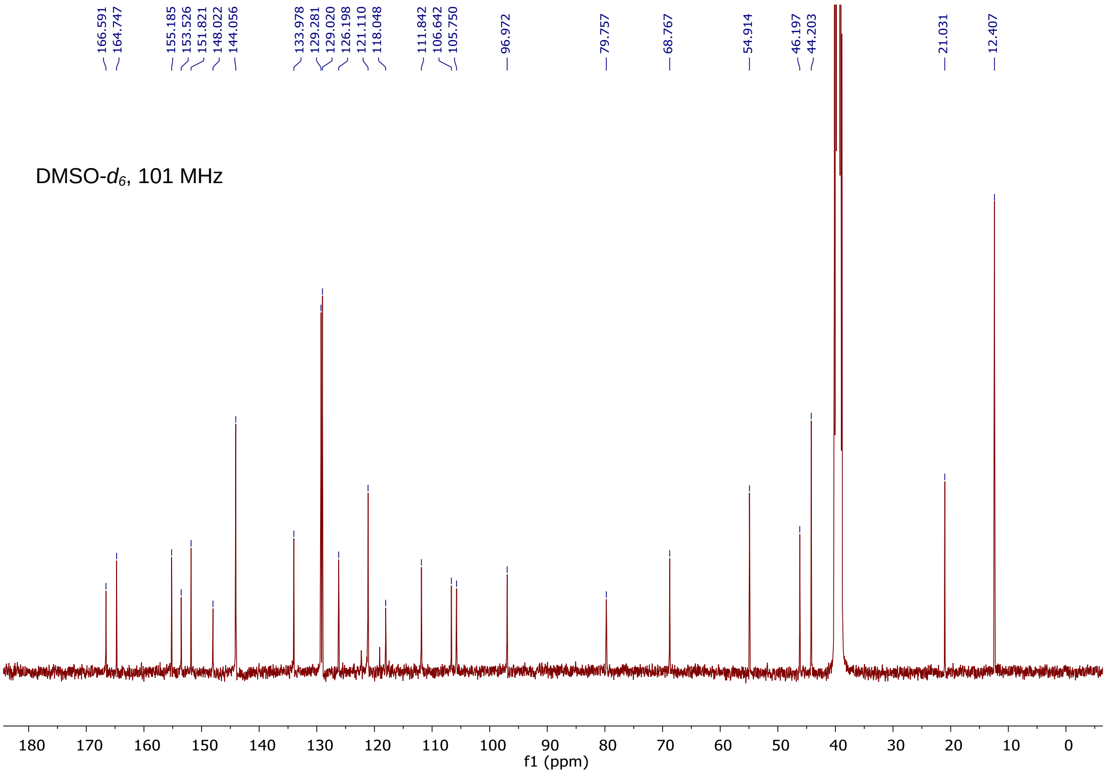

166.591
164.747
155.185
153.526
151.821
148.022
144.056
133.978
129.281
129.020
126.198
121.110
118.048
111.842
106.642
105.750
96.972
79.757
68.767
54.914
46.197
44.203
21.031
12.407
DMSO-d_6, 101 MHz
f1 (ppm)

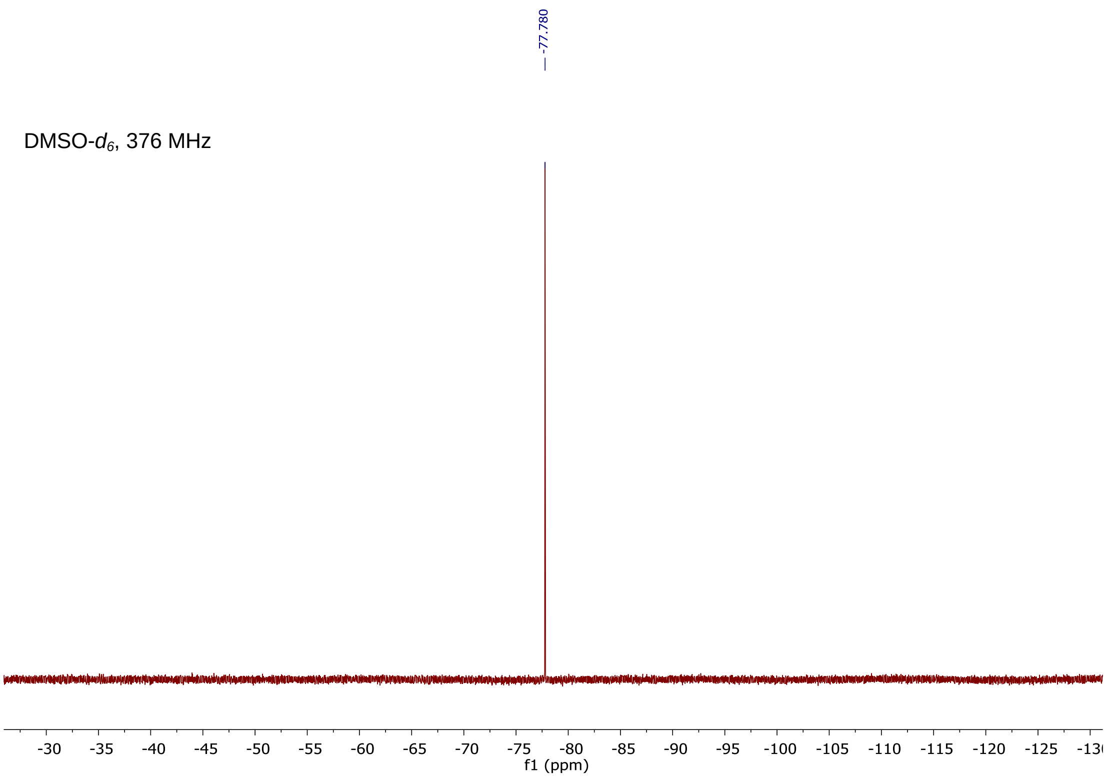

Figure S50. ^{1}H, ^{13}C and ^{19}F NMR spectra of compound **3** in DMSO-*d*6.

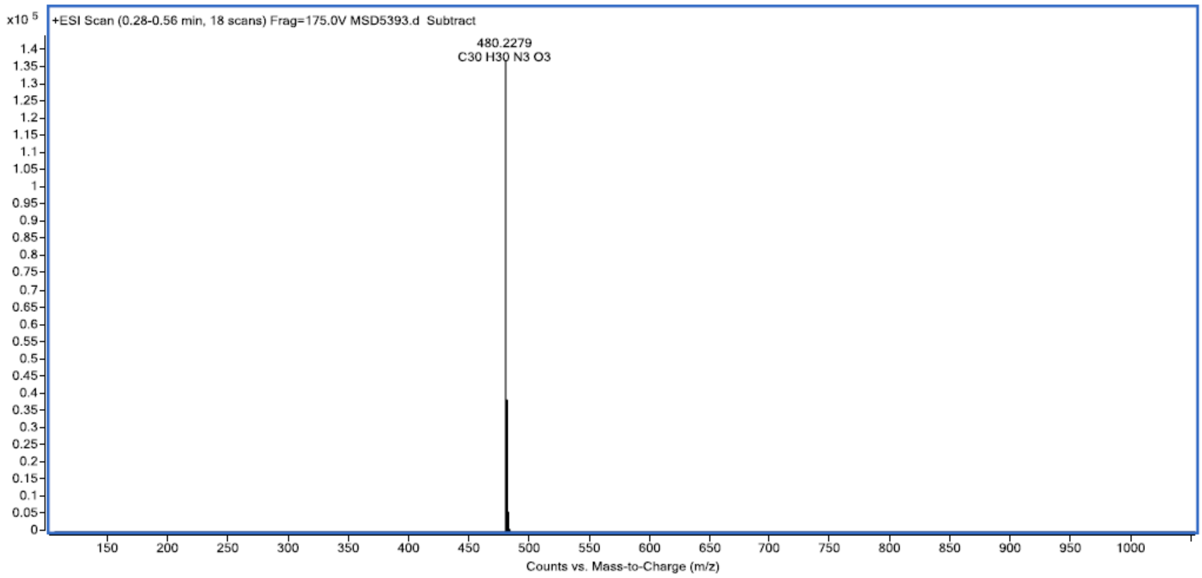

Figure S51. HR ESI-MS spectrum of compound **3**.

Compound 4

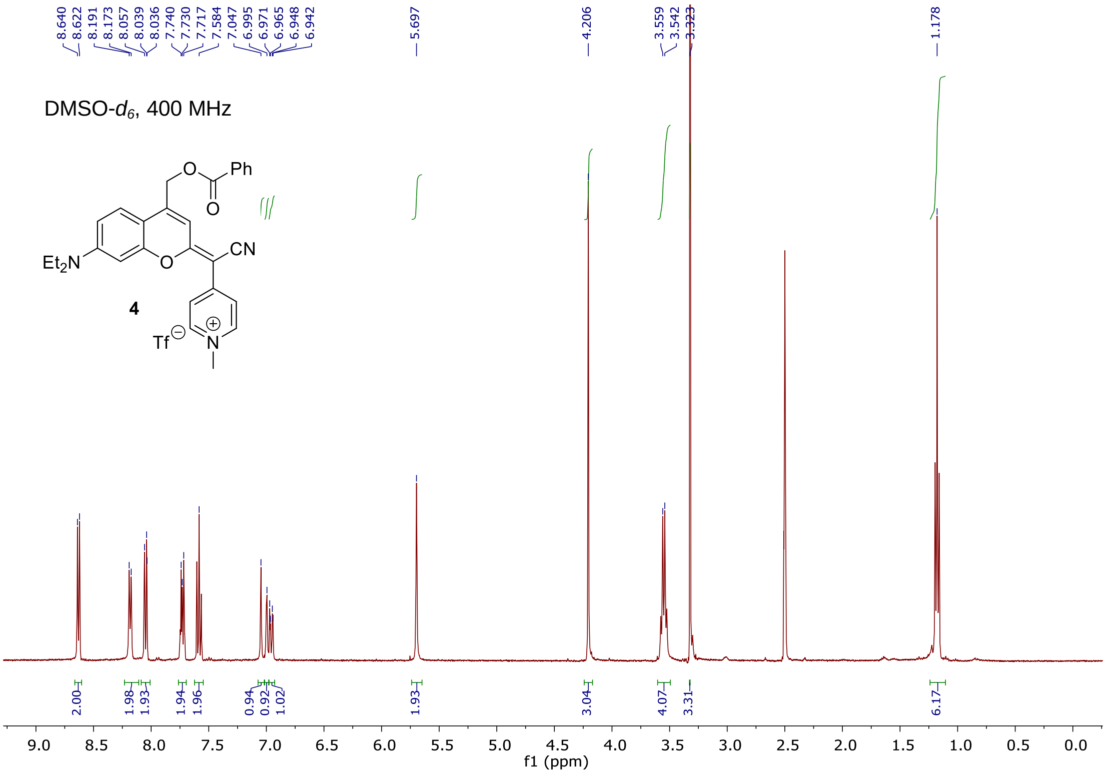
8.640
8.622
8.191
8.173
8.057
8.039
8.036
7.740
7.730
7.717
7.584
7.047
6.995
6.971
6.965
6.948
6.942
5.697
4.206
3.559
3.542
3.323
1.178
DMSO-d_6, 400 MHz
Ph
O
O
CN
Et_2N
O
4
Tf
N
2.00
1.98
1.93
1.94
1.96
0.94
0.92
1.02
1.93
3.04
4.07
3.31
6.17
9.0 8.5 8.0 7.5 7.0 6.5 6.0 5.5 5.0 4.5 4.0 3.5 3.0 2.5 2.0 1.5 1.0 0.5 0.0
f1 (ppm)

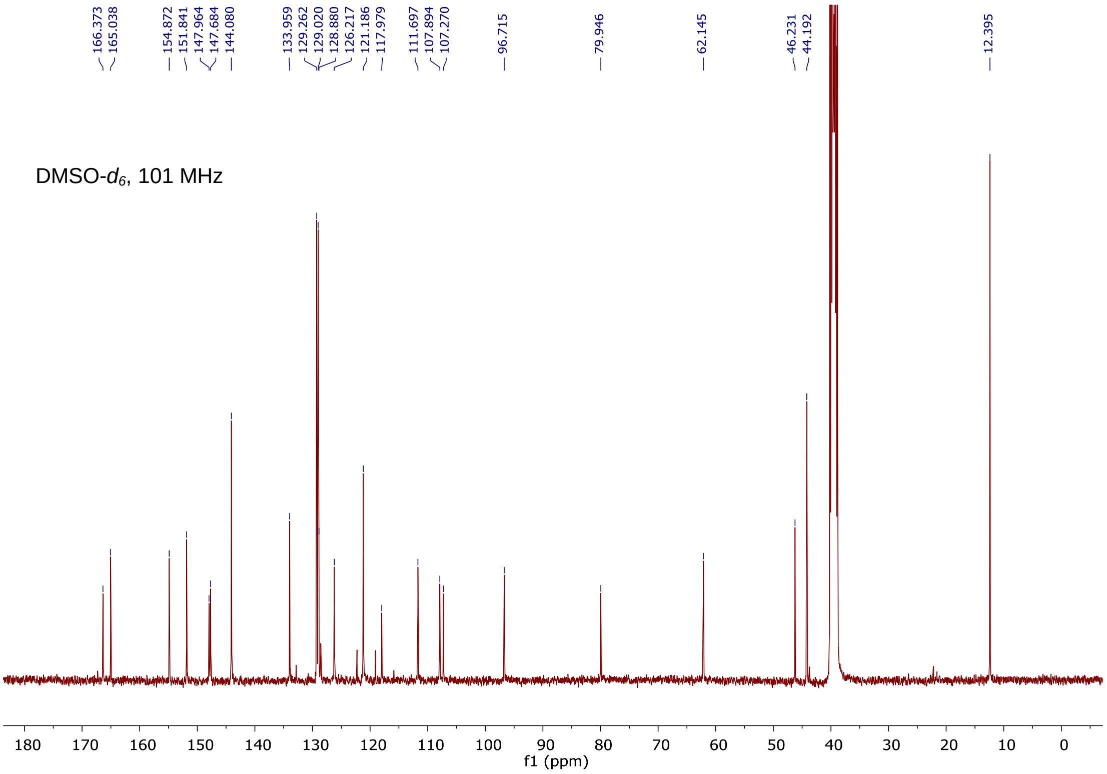
166.373
165.038
154.872
151.841
147.964
147.684
144.080
133.959
129.262
129.020
128.880
126.217
121.186
117.979
111.697
107.894
107.270
96.715
79.946
62.145
46.231
44.192
12.395
DMSO-d_6, 101 MHz
180 170 160 150 140 130 120 110 100 90 80 70 60 50 40 30 20 10 0
f1 (ppm)

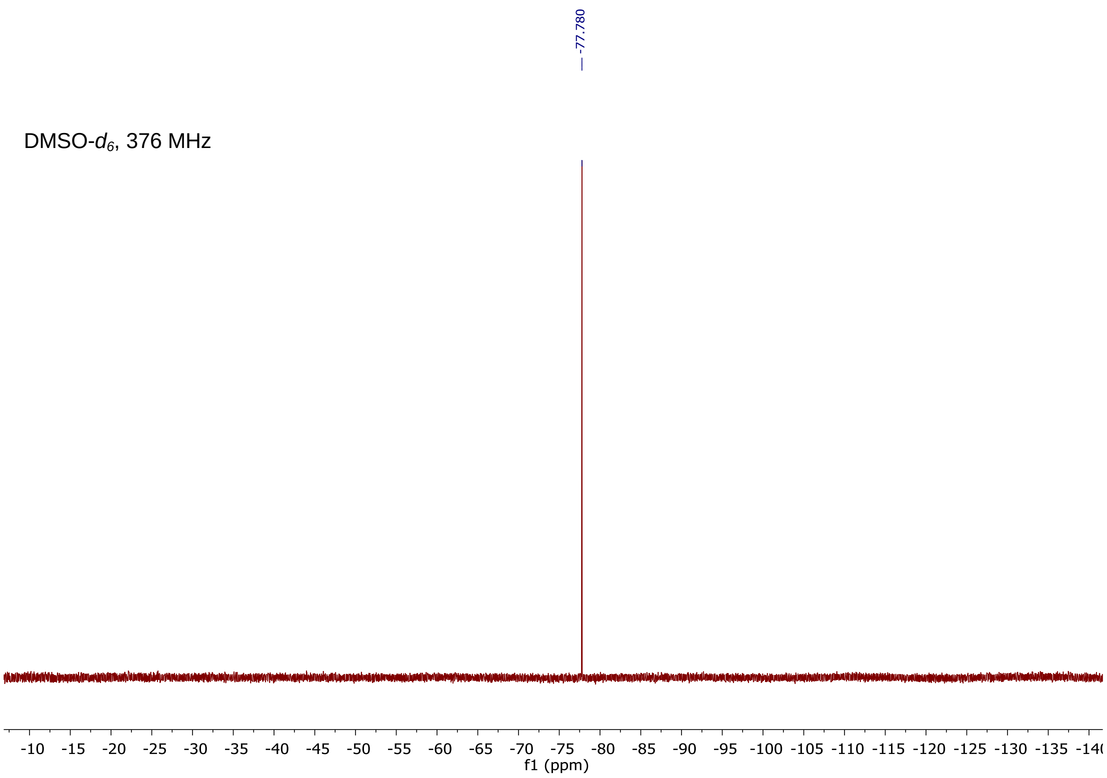

Figure S52. ^{1}H, ^{13}C and ^{19}F NMR spectra of compound **4** in DMSO-d_6.

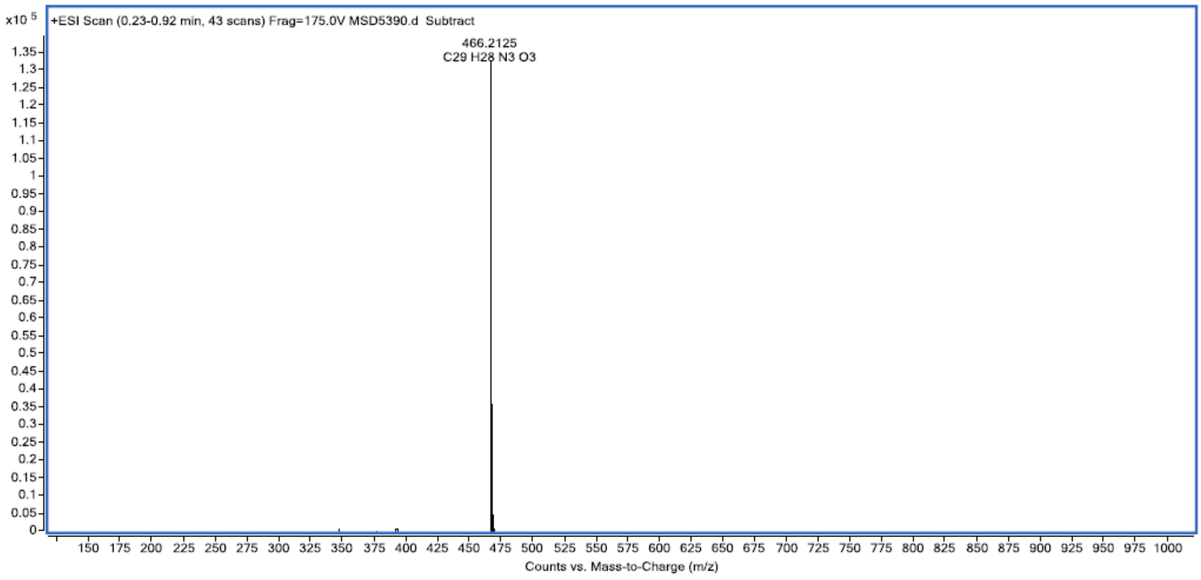

Figure S53. HR ESI-MS spectrum of compound **4**

Compound 5

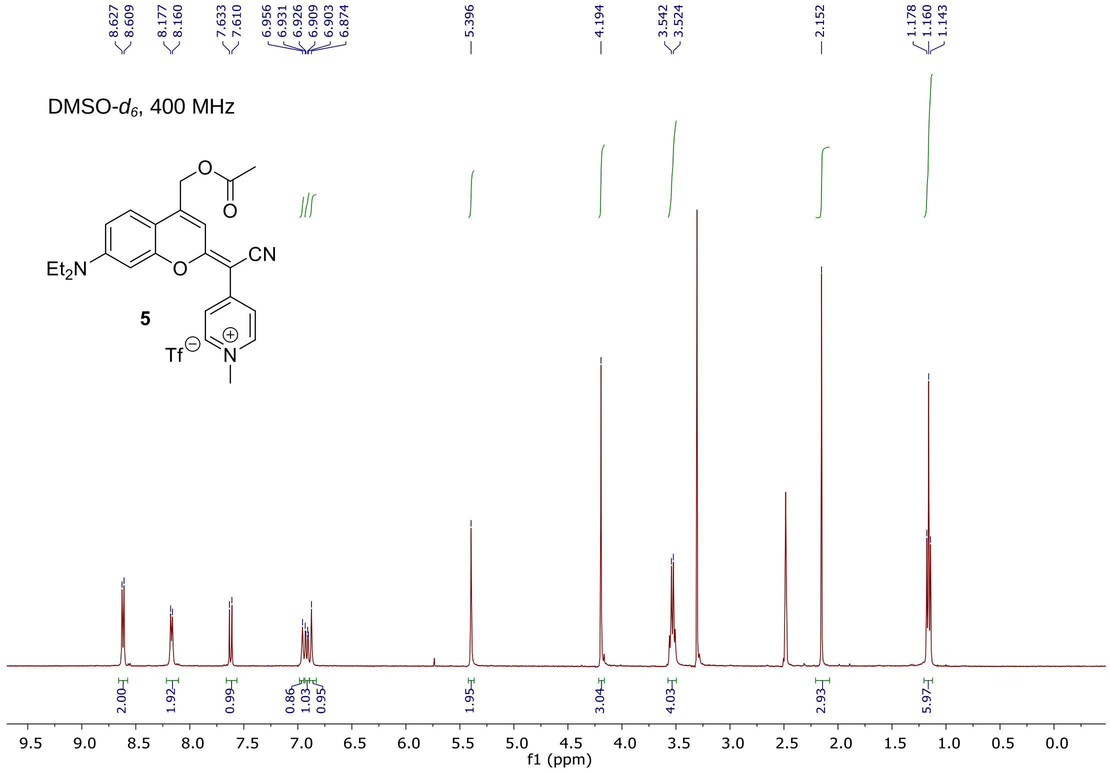
8.627
8.609
8.177
8.160
7.633
7.610
6.956
6.931
6.926
6.909
6.903
6.874
5.396
4.194
3.542
3.524
2.152
1.178
1.160
1.143
DMSO-d_6, 400 MHz
Et$_2$N
O
CN
5
Tf
N
2.00
1.92
0.99
0.86
1.03
0.95
1.95
3.04
4.03
2.93
5.97
f1 (ppm)

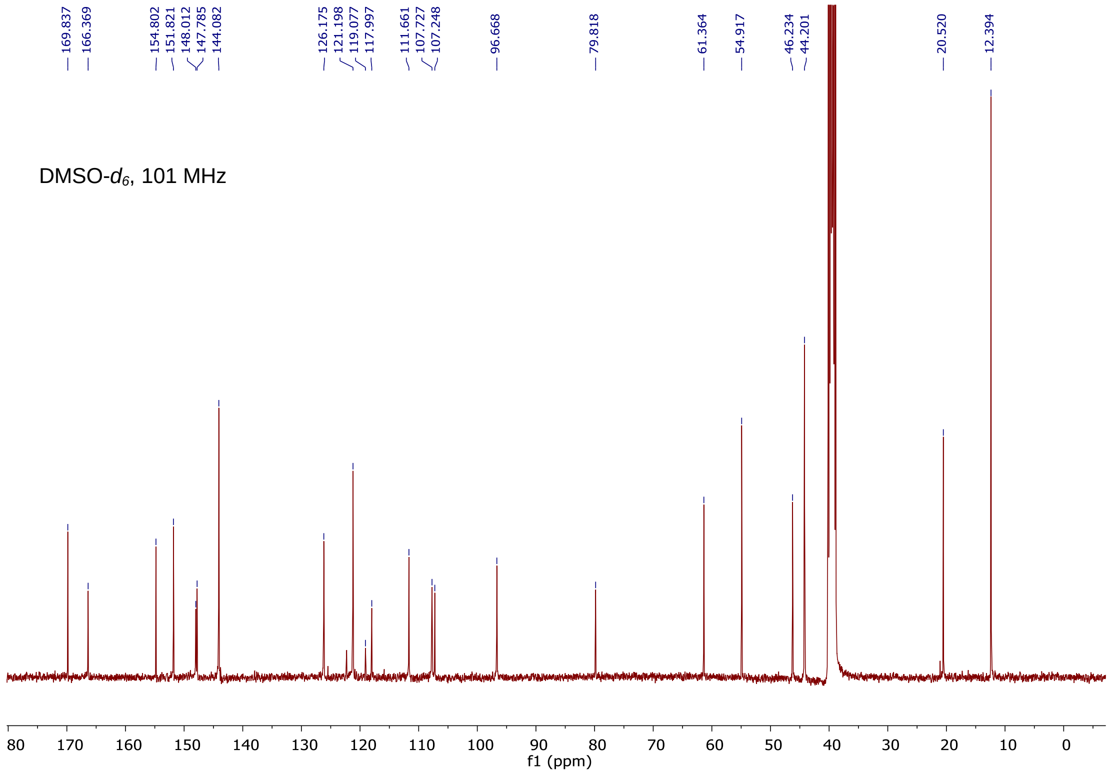
169.837
166.369
154.802
151.821
148.012
147.785
144.082
126.175
121.198
119.077
117.997
111.661
107.727
107.248
96.668
79.818
61.364
54.917
46.234
44.201
20.520
12.394
DMSO-d_6, 101 MHz
f1 (ppm)

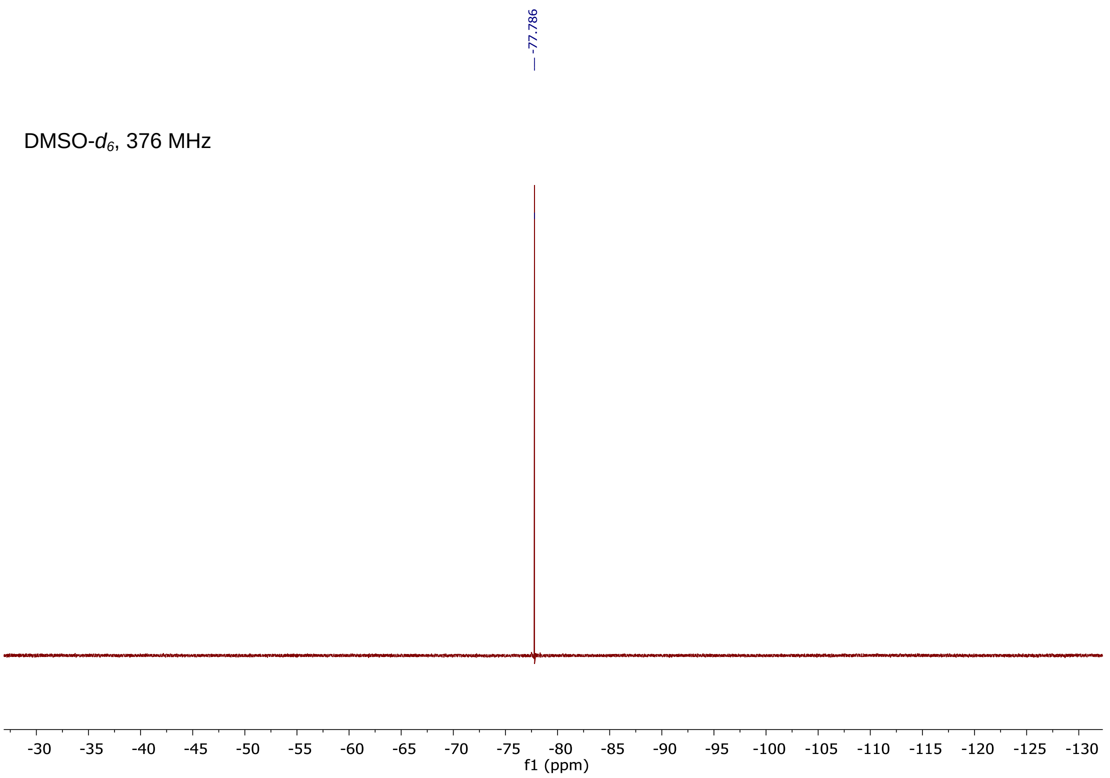

Figure S54. ^{1}H, ^{13}C and ^{19}F NMR spectra of compound **5** in DMSO-d_6.

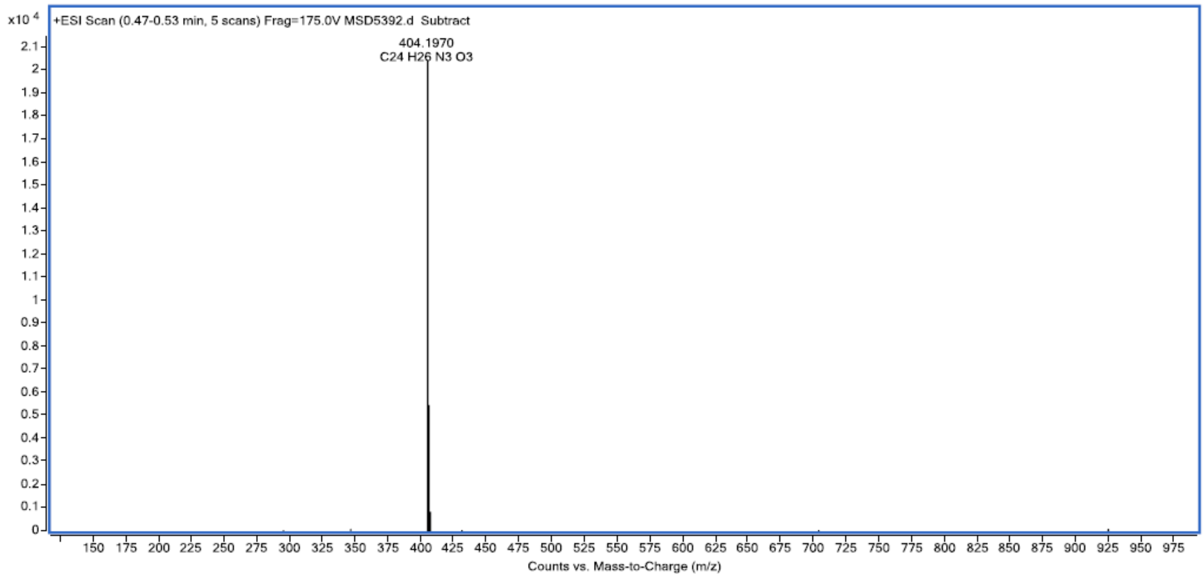

Figure S55. HR ESI-MS spectrum of compound **5**.

Compound 6

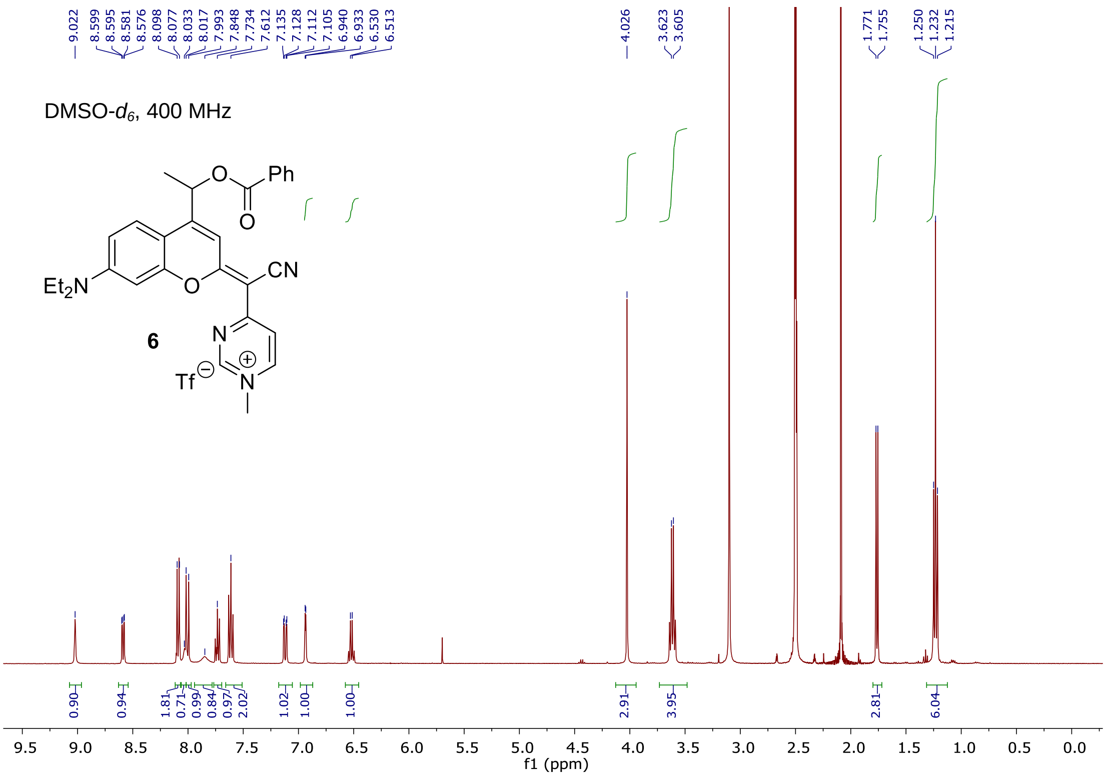

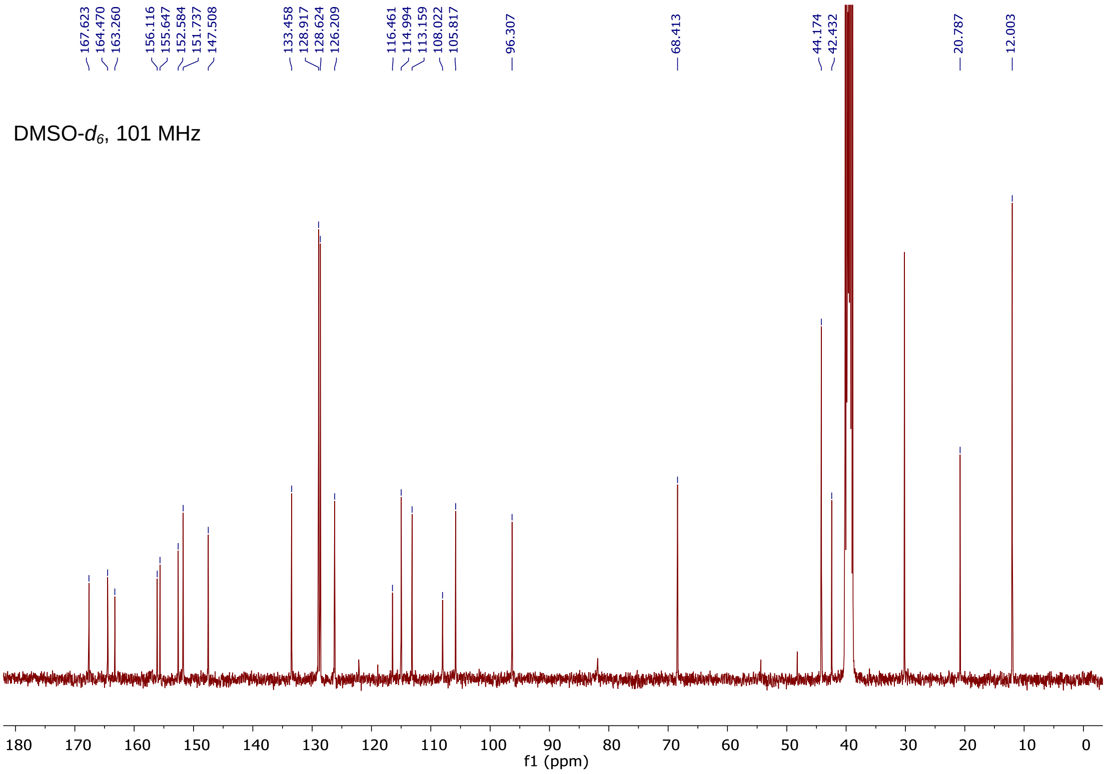

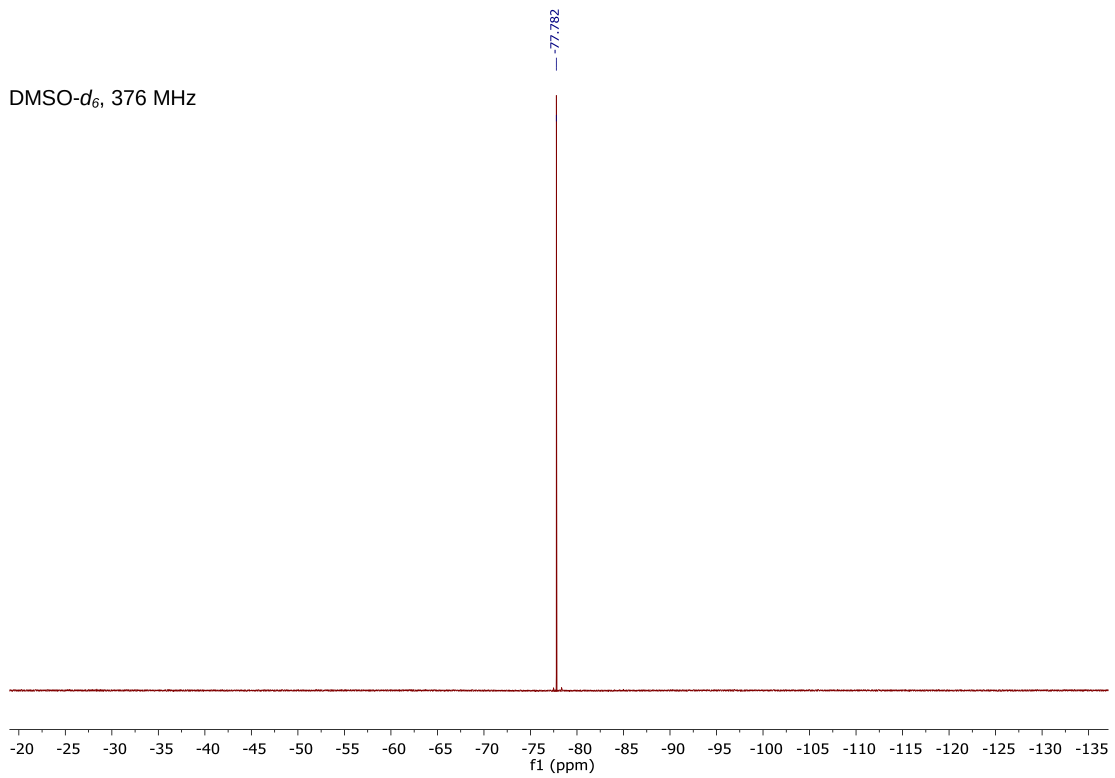

Figure S56. ^{1}H, ^{13}C and ^{19}F NMR spectra of compound **6** in DMSO-d_6. ^{1}H and ^{13}C NMR spectra were recorded at 348 K.

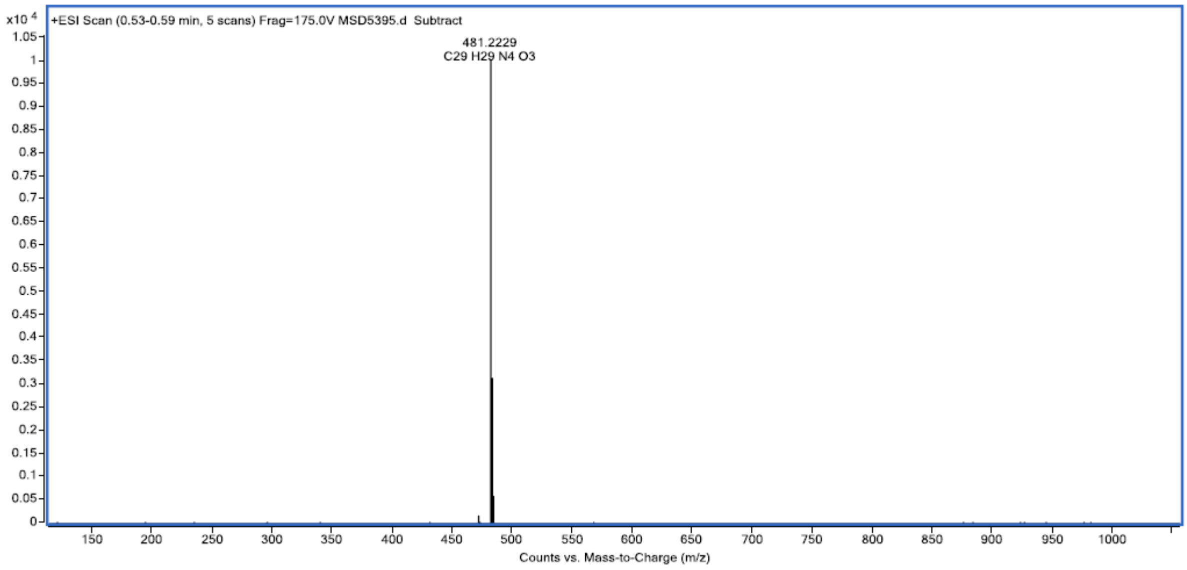

Figure S57. HR ESI-MS spectrum of compound **6**.

Compound 7

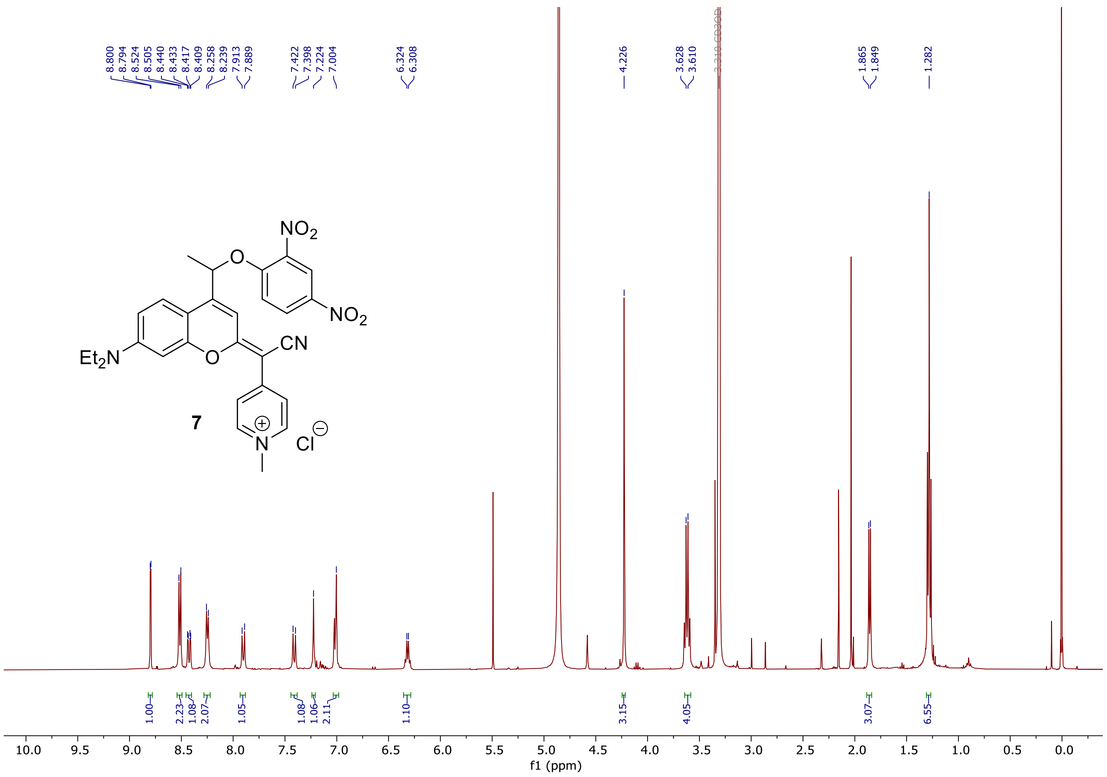

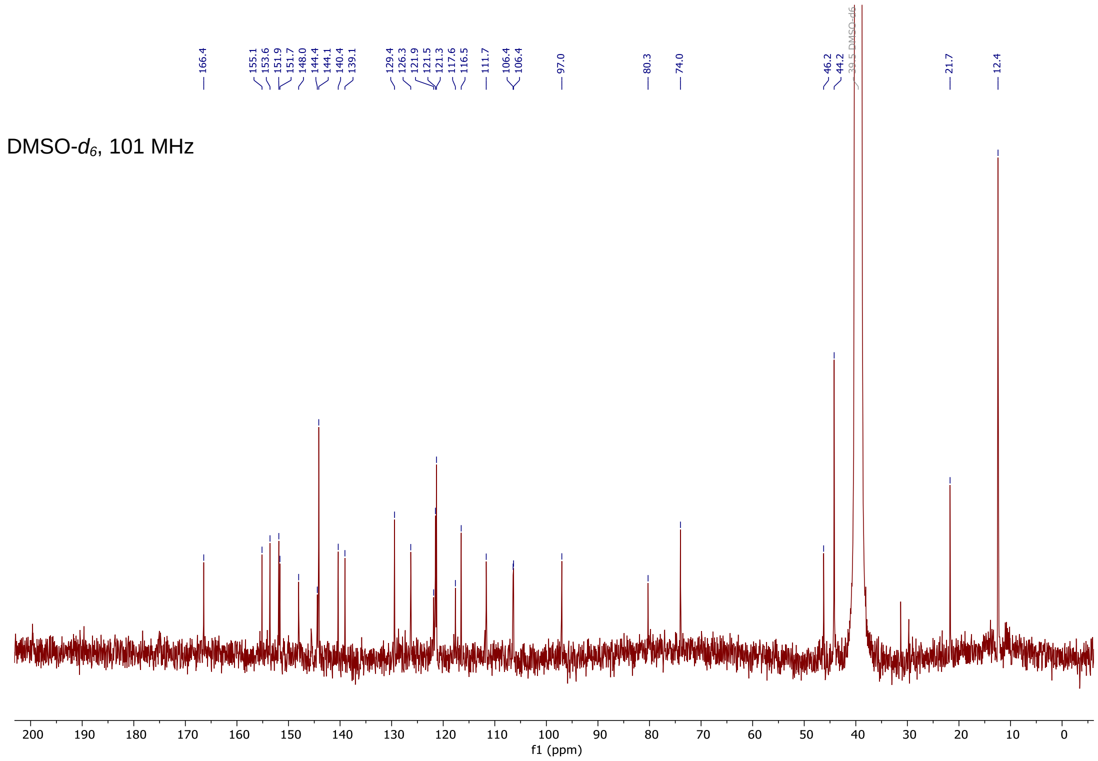

Figure S58. ^{1}H and ^{13}C NMR spectra of compound **7** in CD_3OD and DMSO-d_6, respectively.

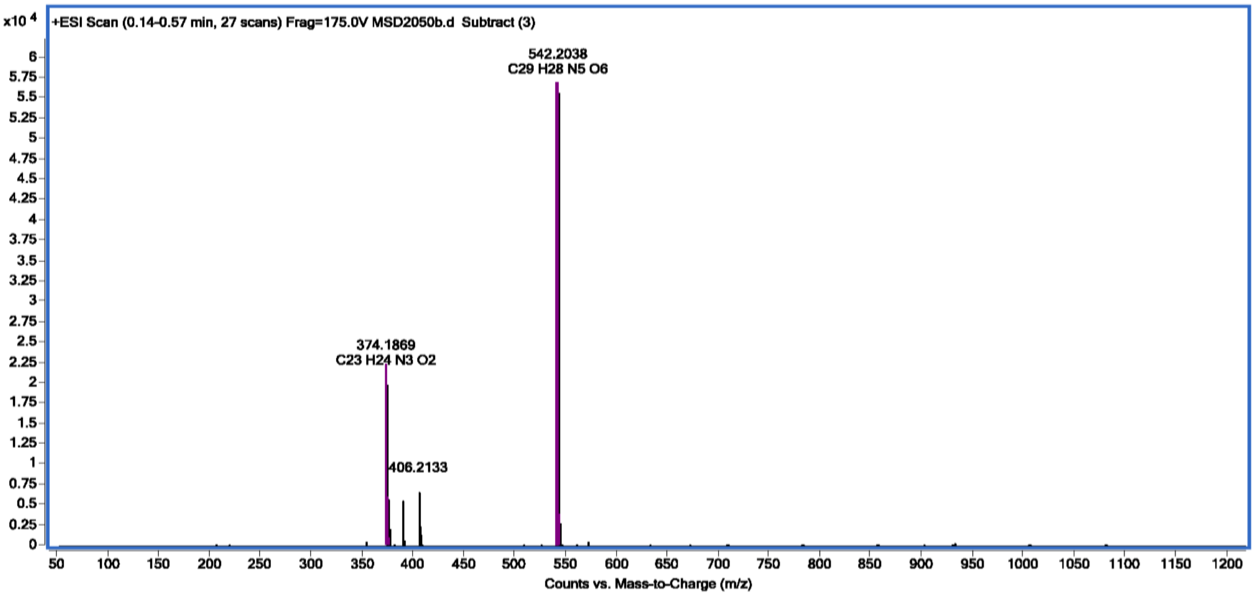

Figure S59. HR ESI-MS spectrum of compound **7.**

Compound 8

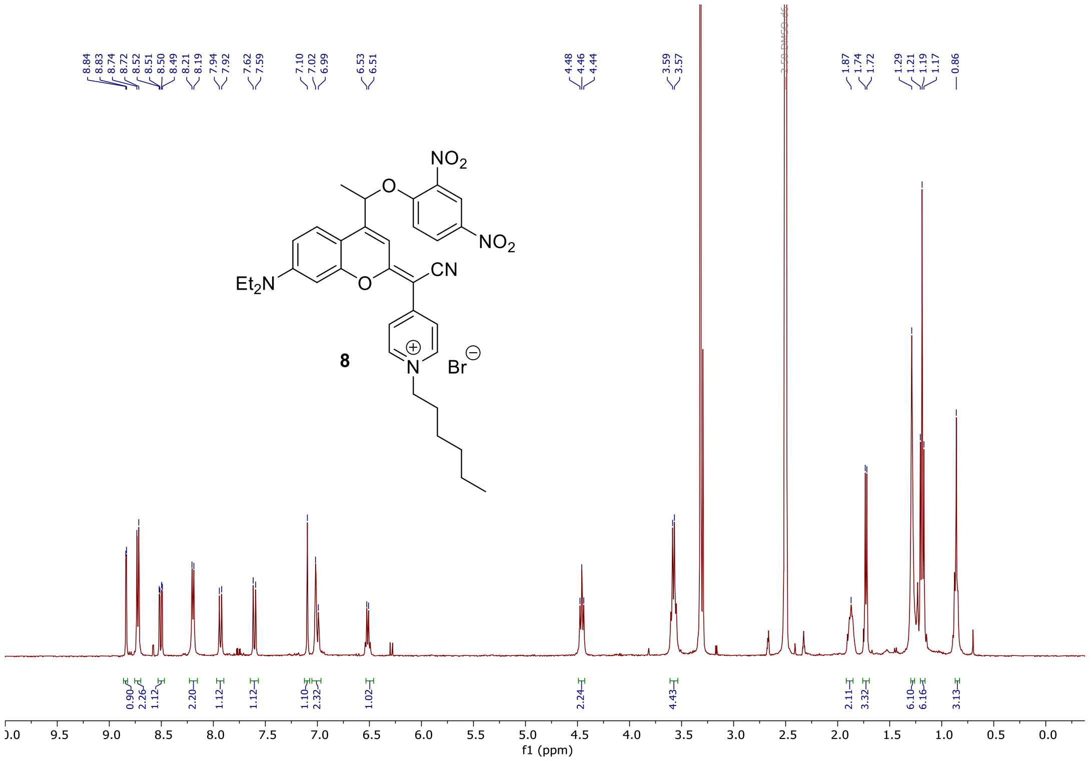

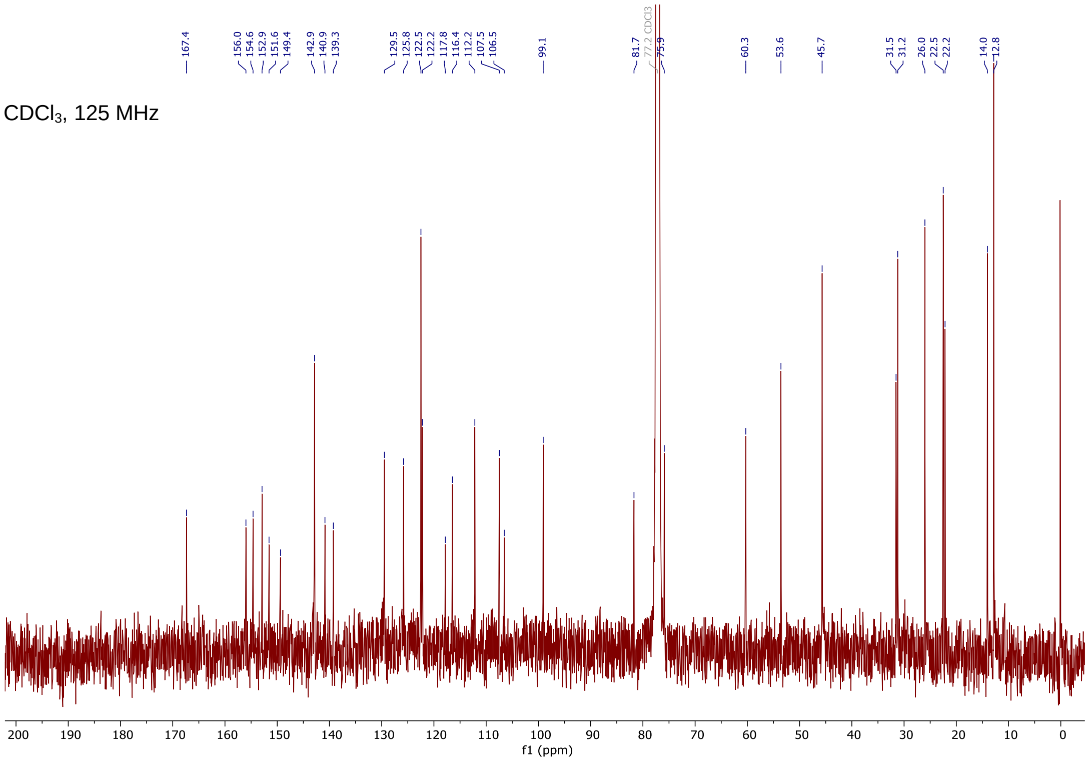

Figure S60. ^{1}H and ^{13}C NMR spectra of compound **8** in DMSO-d_6 and $CDCl_3$, respectively.

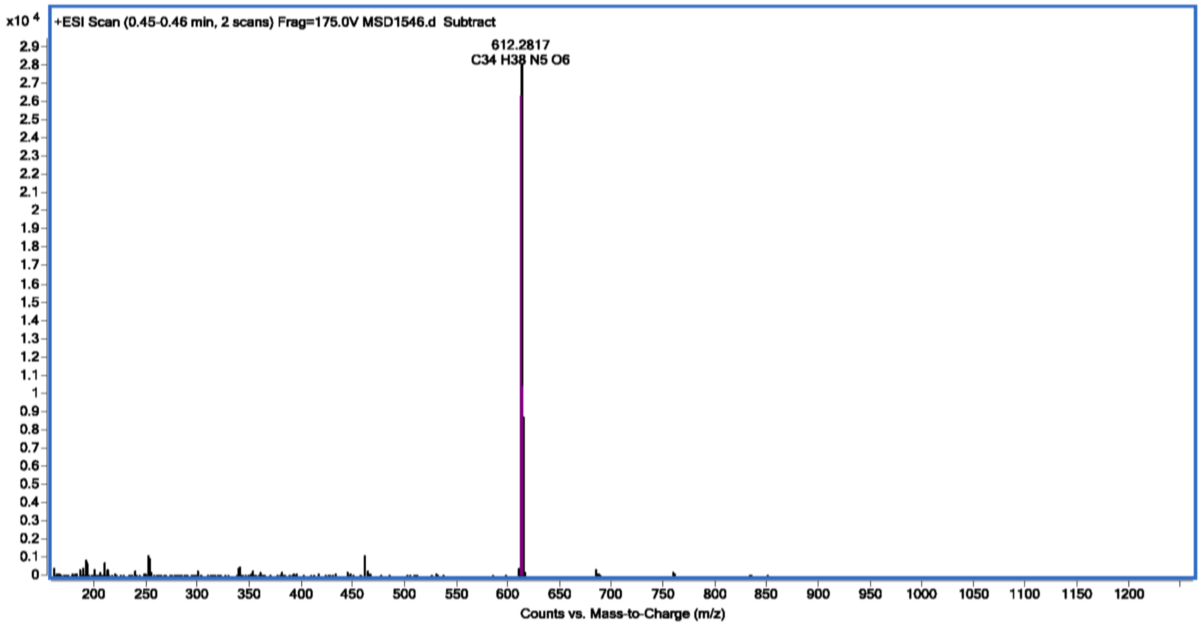

Figure S61. HR ESI-MS spectrum of compound **8**.
